# Large-scale phylogenomic analysis resolves a backbone phylogeny in ferns

**DOI:** 10.1093/gigascience/gix116

**Published:** 2017-11-24

**Authors:** Hui Shen, Dongmei Jin, Jiang-Ping Shu, Xi-Le Zhou, Ming Lei, Ran Wei, Hui Shang, Hong-Jin Wei, Rui Zhang, Li Liu, Yu-Feng Gu, Xian-Chun Zhang, Yue-Hong Yan

**Affiliations:** 1 Shanghai Chenshan Plant Science Research Center, Chinese Academy of Sciences, 3888 Chenhua Road, Songjiang, Shanghai 201602, China; 2 Shanghai Key Laboratory of Plant Functional Genomics and Resources, Shanghai Chenshan Botanical Garden, 3888 Chenhua Road, Songjiang, Shanghai 201602, China; 3 Majorbio Bioinformatics Research Institute, Building 3, Lane 3399, Kangxin Road, International Medical Park, Pudong, Shanghai 201320, China; 4 State Key Laboratory of Systematic and Evolutionary Botany, Institute of Botany, Chinese Academy of Sciences, No. 20 Nanxincun, Xiangshan, Beijing 100093, China

**Keywords:** phylogenomic, monilophytes, evolution, sporangium, transcriptome

## Abstract

**Background:**

Ferns, originated about 360 million years ago, are the sister group of seed plants. Despite the remarkable progress in our understanding of fern phylogeny, with conflicting molecular evidence and different morphological interpretations, relationships among major fern lineages remain controversial.

**Results:**

With the aim to obtain a robust fern phylogeny, we carried out a large-scale phylogenomic analysis using high-quality transcriptome sequencing data, which covered 69 fern species from 38 families and 11 orders. Both coalescent-based and concatenation-based methods were applied to both nucleotide and amino acid sequences in species tree estimation. The resulting topologies are largely congruent with each other, except for the placement of *Angiopteris fokiensis, Cheiropleuria bicuspis, Diplaziopsis brunoniana, Matteuccia struthiopteris, Elaphoglossum mcclurei*, and *Tectaria subpedata*.

**Conclusions:**

Our result confirmed that Equisetales is sister to the rest of ferns, and Dennstaedtiaceae is sister to eupolypods. Moreover, our result strongly supported some relationships different from the current view of fern phylogeny, including that Marattiaceae may be sister to the monophyletic clade of Psilotaceae and Ophioglossaceae; that Gleicheniaceae and Hymenophyllaceae form a monophyletic clade sister to Dipteridaceae; and that Aspleniaceae is sister to the rest of the groups in eupolypods II. These results were interpreted with morphological traits, especially sporangia characters, and a new evolutionary route of sporangial annulus in ferns was suggested. This backbone phylogeny in ferns sets a foundation for further studies in biology and evolution in ferns, and therefore in plants.

## Background

Phylogeny, which reflects natural history, is fundamental to understanding evolution and biodiversity. Ferns (monilophytes), originated about 360 million years (MY) ago, are the sister group of seed plants [1, 2]. With estimated 10 578 extant living species globally [3], they are the second most diverse group of vascular plants. Phylogenetic studies for ferns, especially based on molecular evidence, have been widely carried out in recent decades. These studies have revolutionized our understanding of the evolutionary history of ferns. Milestones included setting ferns as the sister group of seed plants [1, 2], placing Psilotaceae and Equisetaceae within ferns [2, 4, 5], and revealing a major polypods radiation following the rise of angiosperms [6, 7]. Resolutions at shallow phylogenetic depth among families or genera have also been improved remarkably [8–14].

**Figure 1:**
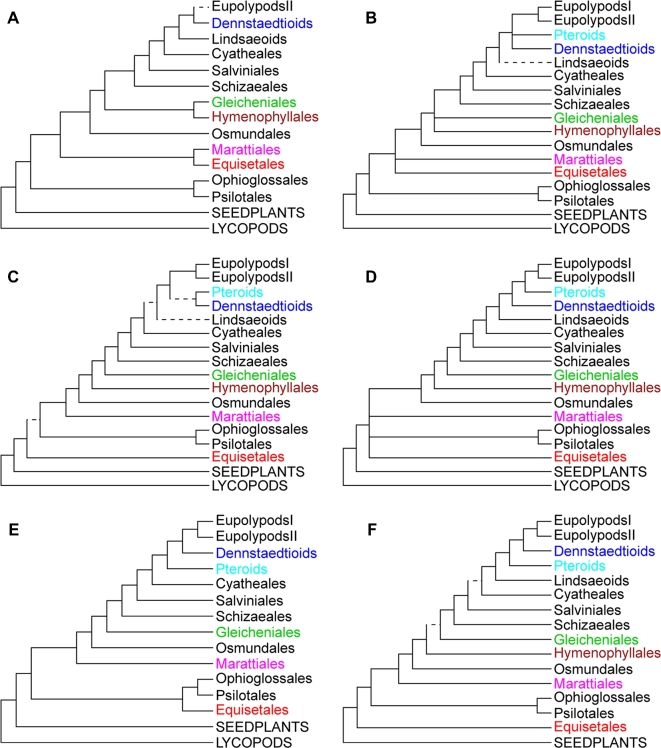
Topologies (a-f) adapted from published results [5, 12–14, 26, 34]. Branches with support <75% were shown using dotted lines, and taxa that differ in their phylogeny locations were shown in different colors.

However, previous research on fern phylogeny has mostly relied on plastid genes [10, 12, 13], some combined with a few nuclear genes [4, 5, 14] or morphological traits [5, 11]. Due to incomplete lineage sorting (ILS), genes from different resources often show conflicting evolutionary patterns, especially when based on a limited number of samples, and some deep relationships in fern phylogeny remain controversial (Fig. 1). In the latest PPG I system [3], which has derived from many recent phylogenetic studies, some important nodes remain uncertain, such as (i) what are the relationships among Marattiales, Ophioglossales, and Psilotales?; (ii) are Hymenophyllales and Gleicheniales sister groups?; and (iii) what are the relationships among families in eupolypods II?

Transcriptome sequencing (RNA-Seq) provides massive transcript information from the genome. Phylogenetic reconstructions based on RNA-Seq are more efficient and cost-effective than traditional polymerase chain reaction–based or expressed sequence tags (EST)-based methods when lacking whole-genome data [15]. Successful cases in recent years include mollusks [16], insects [17], the grape family [18], angiosperms [19], and land plants, including 6 ferns [20]. Here, with the aim to reconstruct the framework of fern phylogeny, we sampled abundant fern species representing all important linages and applied the latest phylogenomic analyses based on RNA-Seq.

To reconstruct a robust and well-resolved phylogeny in ferns, applying multiple methods of phylogenomic analysis is extremely important. Since concatenation-based estimations of species trees usually have good accuracy under a low level of ILS, while coalescent-based methods are developed to overcome the effect of ILS but are sensitive to gene tree estimation error [21], both concatenation-based and coalescent-based estimations are applied. Nucleotide sequence, with higher variability than amino acid sequence, usually brings more useful information in phylogeny reconstruction, especially for closely related taxa. However, the substitutional saturation and compositional bias in nucleotide sequence, especially in the third codon position, may lead to a deviation from the true phylogeny. Here, both nucleotide and amino acid sequences are used in phylogeny reconstruction.

Morphologically, the fern sporangium is an organ for enclosing and dispersing spores, most of which function like a unique catapult with the annulus [22]. During the last centuries, Bower's hypothesis on the evolution of sporangia with a focus on annulus [23] has been one of the most important cornerstones to fern phylogeny based on morphology [24, 25]. However, this hypothesis has been challenged by somewhat conflicting frameworks of fern phylogeny [4, 10, 12, 14, 26]. A robust framework in fern phylogeny that reflects the evolutionary history will improve our understanding of the evolution of fern sporangia as well as other characters.

## Data Description

### Taxa sampling and RNA-Seq

We chose 69 fern species from 38 families according to the PPG I system (48 fern families in total), covering all the 11 orders (Equisetales, Psilotales, Ophioglossales, Marattiales, Osmundales, Hymenophyllales, Gleicheniales, Schizaeales, Salviniales, Cyatheales, and Polypodiales). Information about the location and time for sampling is given in Table S1. All the sampled species were collected under the permissions of the natural reserves and Shanghai Chenshan Botanical Garden in China.

Sporophyll or/and trophophyll were collected and frozen in liquid nitrogen immediately, and preserved in an ultra-low-temperature refrigerator at –80°C before RNA extraction. Total RNA was extracted using TRIzol (Life Technologies Corp., Carlsbad, California, USA) according to the manufacturer's protocols. The RNA concentration was determined using a NanoDrop spectrophotometer, and RNA quality was assessed with an Agilent Bioanalyzer. Paired-end reads were generated by Majorbio Company (Shanghai, China) using the HiSeq 2500 system. Raw reads were deposited in NCBI [27].

### Transcriptomes assembly and orthology assignment

Transcriptomes data were generated from 69 fern species (Table 1). After filtering, about 2726.9 million paired-end DNA sequence reads (about 313 Gbp) were retained. We assembled these reads *de novo* and obtained a total of 5 449 842 contigs [28].

**Table 1:** Sequencing and assembly information of the transcriptome data

ID	Species	Clean data, G	Total reads (clean)	Q30 %	Number of contigs	N50, bp	Mean, bp	Genes in Matrix 1	Genes in Matrix 2
RS1	*Pronephrium simplex*	4.7	38 045 864	91.24	151 319	887	581.07	2168	1254
RS10	*Antrophyum callifolium*	4.0	32 745 384	91.76	64 107	1819	998.73	2226	1305
RS101	*Oleandra musifolia*	4.5	36 487 068	91.45	37 075	1493	919.3	2093	1248
RS103	*Woodsia polystichoides*	3.9	31 465 870	90.91	47 812	1348	811.3	2287	1310
RS107	*Equisetum diffusum*	4.4	35 693 238	90.21	88 932	1154	655.64	1811	1254
RS108	*Oreogrammitis dorsipila*	4.6	37 037 324	90.57	266 540	591	485.1	2141	1273
RS11	*Vandenboschia striata*	4.8	38 639 790	90.3	261 724	460	422.76	1959	1276
RS111	*Pleurosoriopsis makinoi*	4.8	38 983 796	90.13	98 187	1145	632.29	2182	1277
RS112	*Azolla pinnata subsp. asiatica*	4.4	35 735 206	90.57	78 295	1348	777.92	1418	839
RS114	*Taenitis blechnoides*	4.1	32 898 682	90.98	70 495	1262	711.3	2186	1278
RS115	*Gymnogrammitis dareiformis*	3.9	31 630 988	89.81	119 483	569	449.38	1996	1220
RS116	*Schizaea dichotoma*	4.5	36 668 734	89.6	67 422	1350	826.92	2035	1285
RS119	*Botrychium japonicum*	4.8	38 603 000	90.28	85 236	1477	846.97	1866	1283
RS122	*Goniophlebium niponicum*	4.8	38 786 214	90.82	54 152	1663	951.92	2279	1300
RS123	*Arthropteris palisotii*	4.4	35 646 740	91	50 700	1454	891.67	2286	1311
RS124	*Matteuccia struthiopteris*	4.2	34 080 998	90.44	57 514	1345	776.52	2290	1313
RS127	*Salvinia natans*	4.2	33 780 056	91.17	79 393	1379	767.14	1905	1173
RS128	*Woodwardia prolifera*	5.1	40 967 322	91.63	69 931	1557	859.72	2328	1328
RS14	*Diplazium viridescens*	4.0	32 320 416	90.46	88 236	1434	780.87	2269	1310
RS16	*Bolbitis appendiculata*	4.7	37 503 336	91.66	201 426	802	556.39	2226	1288
RS17	*Dryopteris pseudocaenopteris*	4.1	33 136 196	91.23	102 751	723	514.92	2236	1298
RS18	*Dicranopteris pedata*	4.2	33 942 120	92.04	74 011	1193	684.09	2031	1304
RS19	*Haplopteris amboinensis*	4.2	42 772 168	94.17	47 603	1713	1041.8	2249	1307
RS21	*Psilotum nudum*	8.5	85 199 034	93.6	66 212	1739	927.19	1741	1223
RS24	*Cyclopeltis crenata*	4.6	37 158 058	91.5	29 668	600	491.82	2146	1279
RS25	*Asplenium formosae*	4.6	46 629 754	93.5	73 318	1722	989.84	2273	1312
RS27	*Lomariopsis spectabilis*	4.1	33 233 594	91.77	98 030	1466	750.42	2225	1304
RS28	*Cheiropleuria bicuspis*	5.1	41 617 294	91.35	99 411	1435	832.82	2022	1295
RS31	*Plagiogyria japonica*	5.7	46 472 760	91.92	89 532	1258	733.9	2036	1222
RS34	*Alsophila podophylla*	4.9	48 768 608	93.43	66 254	1580	904.62	2195	1289
RS35	*Histiopteris incisa*	4.3	43 115 390	93.81	61 231	1749	985.03	2319	1316
RS36	*Pteris vittata*	4.1	41 212 858	94.37	76 666	1868	1021.13	2296	1312
RS37	*Cibotium barometz*	4.1	33 263 550	91.92	85 555	1612	891.87	1790	1099
RS38	*Osmunda japonica*	4.1	33 485 274	92.05	58 612	1730	901.28	1732	1159
RS39	*Loxogramme chinensis*	3.9	31 392 952	92.16	84 796	1065	651.88	2240	1305
RS4	*Microlepia hookeriana*	4.0	40 561 422	94.49	95 951	1610	874.06	2262	1301
RS41	*Pteridium aquilinum*	4.6	46 157 134	93.51	55 615	1742	960.37	2321	1316
RS42	*Hypolepis punctata*	4.4	43 828 154	93.56	59 717	1371	833.68	2277	1308
RS43	*Dicksonia antarctica*	3.9	31 210 608	91.69	56 494	1533	902.96	2045	1213
RS45	*Rhachidosorus mesosorus*	4.4	35 348 994	91.98	80 069	1541	835.92	2300	1315
RS46	*Drynaria bonii*	4.5	36 017 548	92.02	68 132	1077	643.93	2176	1279
RS47	*Platycerium bifurcatum*	4.1	33 209 740	91.62	40 456	1097	694.56	2148	1283
RS48	*Angiopteris fokiensis*	4.4	35 120 302	91.12	57 637	1629	932.57	1917	1306
RS5	*Diplaziopsis brunoniana*	4.3	34 698 846	91.35	70 184	822	541.31	2040	1234
RS50	*Dennstaedtia pilosella*	4.5	45 618 446	93.63	84 813	1582	831.56	2308	1313
RS51	*Monachosorum henryi*	4.1	41 658 504	93.42	87 832	1465	803.17	2255	1288
RS52	*Acystopteris japonica*	5.5	44 662 146	91.15	57 118	1507	873.59	1222	677
RS53	*Monachosorum maximowiczii*	4.8	48 497 004	93.58	101 448	1817	899.54	2257	1294
RS54	*Dennstaedtia scabra*	5.1	51 360 716	93.47	92 158	1565	845.44	1818	1056
RS56	*Arachniodes nigrospinosa*	5.1	50 929 362	94.47	57 168	1623	916.1	2332	1319
RS69	*Cheilanthes chusana*	5.2	51 851 066	94.18	49 449	1727	1012.63	2317	1324
RS7	*Elaphoglossum mcclurei*	4.1	32 800 248	92.31	57 330	1398	846.79	2267	1299
RS70	*Lomagramma matthewii*	4.4	35 218 876	91.21	65 170	1748	947.18	2258	1307
RS71	*Osmolindsaea odorata*	4.6	46 808 646	94.13	113 778	1521	845.96	2257	1312
RS72	*Aleuritopteris chrysophylla*	4.8	47 955 674	94.18	61 637	1669	929.63	2307	1322
RS77	*Marsilea quadrifolia*	4.3	34 724 432	91.76	65 227	1607	930.31	2188	1299
RS8	*Humata repens*	4.5	36 606 746	91.17	68 932	1267	690.35	2264	1315
RS81	*Tectaria subpedata*	4.2	42 539 482	94.43	57 384	1326	797.83	2128	1242
RS84	*Ophioglossum vulgatum*	4.4	35 637 330	91.77	71 821	1226	741.62	1631	1179
RS85	*Nephrolepis cordifolia*	5.0	40 063 236	90.81	55 207	1530	842.63	2302	1319
RS86	*Microlepia platyphylla*	4.6	46 324 294	94	74 956	1763	945.87	2267	1295
RS88	*Lygodium flexuosum*	4.2	34 098 316	91.44	66 751	1514	867.82	2064	1296
RS89	*Hypodematium crenatum*	4.1	32 711 798	91.58	52 813	1416	852.57	2298	1319
RS90	*Acrostichum aureum*	5.4	43 422 574	90.69	46 189	1729	1043.2	2303	1319
RS91	*Adiantum caudatum*	5.1	51 062 204	94.23	51 145	1575	950.49	2323	1327
RS92	*Parahemionitis cordata*	4.1	33 309 450	91.72	47 508	1456	894.42	2306	1317
RS93	*Microlepia speluncae*	4.4	44 124 842	94.55	94 980	1720	917.59	2292	1308
RS97	*Stenochlaena palustris*	4.7	37 887 642	91.81	58 416	1655	945.83	2300	1316
RS98	*Ceratopteris thalictroides*	3.9	31 741 082.0	91.4	74 728	1610	912.26	2231	1296

The number of ortholog genes used in Matrix 1 and Matrix 2 were shown.

In order to obtain a reliable phylogenetic relationship, we selected 4 species as the outgroup, representing the main lineages of land plants: *Amborella trichopoda* (representing angiosperms), *Picea abies* (representing gymnosperms), *Selaginella moellendorffii* (representing lycophytes), and *Physcomitrella patens* (representing bryophytes). The translated ORF (protein) sequences of these 4 species were downloaded from Phytozome [29] and used in the following analysis.

**Figure 2:**
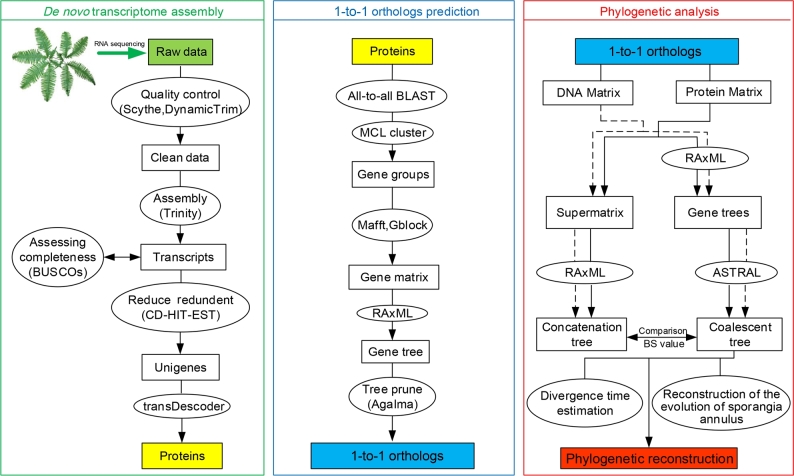
A working flow diagram showing the major processes of data production and analysis in this study. Three major processes are *de novo* transcriptome assembly, 1-to-1 orthologs prediction, and phylogenetic analysis. The rectangles represent the main results, and the ellipses represent the main methods and analysis.

To ensure the consistency of phylogenomic analysis, we used a phylogenetic-based ortholog selection method and obtained 2 subsets of 1-to-1 orthologous genes that differed in gene number and species occupancy rate, named “Matrix 1” and “Matrix 2” [30]. Matrix 1 consists of 2391 genes that are present in at least 52 taxa (that is 75% of the 69 taxa in total), resulting in 2 024 565 nucleotide and 674 855 amino acid positions; the gene and character occupancy were 88% and 85%, respectively. Matrix 2 consists of 1334 genes that are present in at least 62 taxa (that is 90% of the 69 taxa in total), resulting in 1 171 332 nucleotide and 390 444 amino acid positions; the gene and character occupancy reached 94% and 90%, respectively. For each orthologue gene set, coalescent-based and concatenation-based methods were applied separately to both nucleotide and amino acid sequences. A working flow diagram showing the major processes in this study is presented in Fig. 2.

## Results

### Species tree estimated in 69 ferns

**Figure 3:**
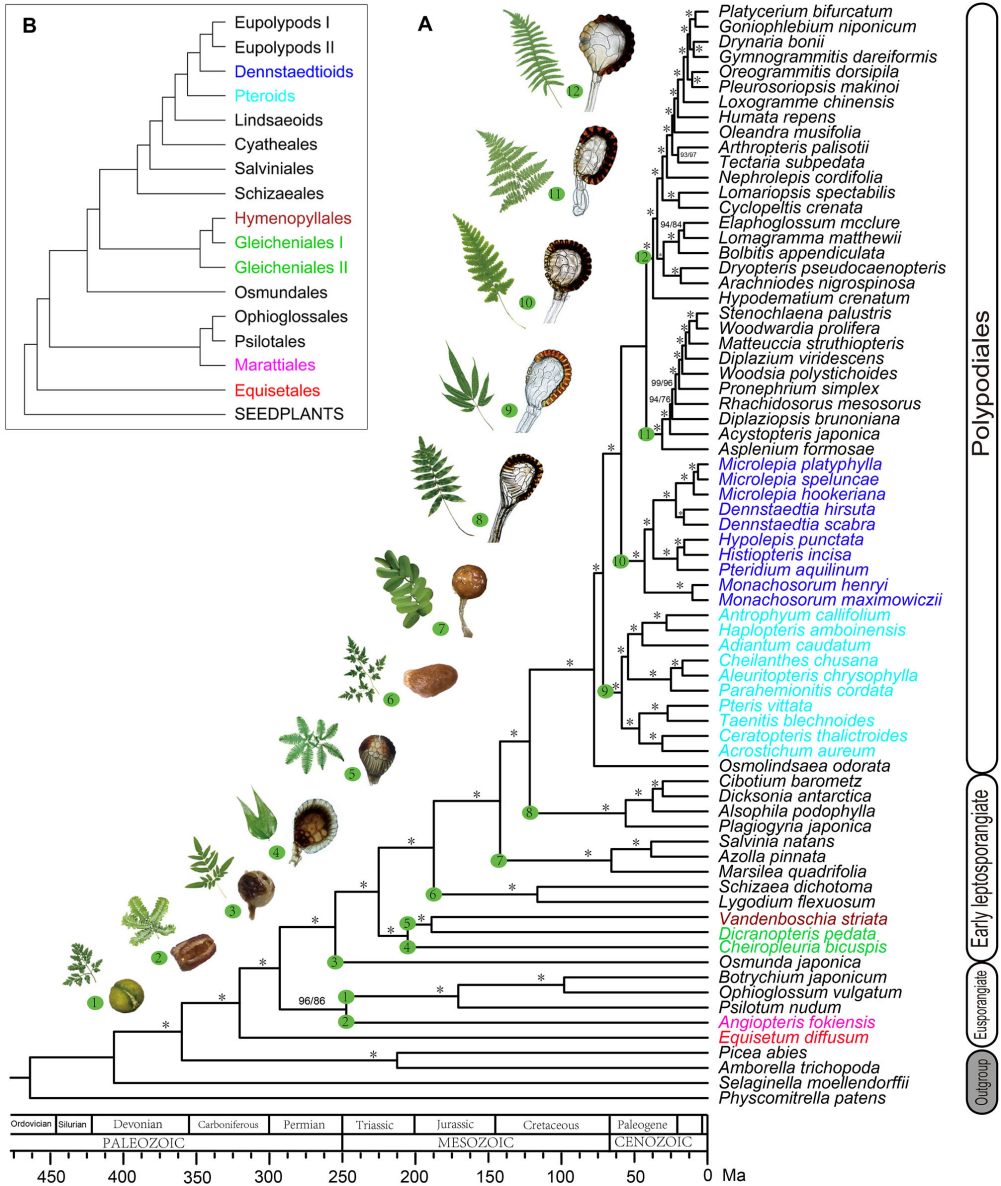
Phylogeny of ferns reconstructed by coalescent-based method using nucleotide sequence with divergence times calculated. Support values for the main phylogeny (A) calculated from Matrix 1/Matrix 2 are listed as percentages. ^*^Indicates 100%/100%. Representative leave(s), sporangium, and the corresponding lineage are labeled with a same number. Simplified topology (B) shows the main linages as in Fig. 1. Species in phylogeny (A) and the corresponding lineage in topology (B) are shown in the same color.

For each combination of reconstruction methods (coalescent-based or concatenation-based) and sequence types (nucleotide or amino acid), Matrix 1 and Matrix 2 [31, 32] always yielded the same topology. In general, the 4 topologies (Fig. 3, Figs S1, S2, S3) from a combination of methods and sequence types are consistent, except for 6 positions (Table 2). Among the topologies, the one estimated by applying a coalescent-based method to the nucleotide sequence (Fig. 3) and the one applying a concatenation-based method (Figure S2) are most congruent.

**Table 2:** Inconsistent topologies using different methods and sequences

	Coalescent-based method		Concatenation-based method	
Site	Nucleotide	Amino acid	Nucleotide	Amino acid
A	**(Anfo,(Pnu,(Ovu,Bja)))**	**(Anfo,(Pnu,(Ovu,Bja)))**	((Pnu,(Ovu,Bja)),(Anfo,^a^))	((Pnu,(Ovu,Bja)),(Anfo,^a^))
B	**(Cbi,(Dpe,Vst))**	**(Cbi,(Dpe,Vst))**	**(Cbi,(Dpe,Vst))**	((Dpe,Vst),(Cbi,^a^))
C	**(Asfo,(Aja,(Dbr,^a^)))**	**(Asfo,(Aja,(Dbr,^a^)))**	**(Asfo,(Aja,(Dbr,^a^)))**	(Asfo,((Aja,Dbr),^a^))
D	**(Dvi,(Mst,(Spa,Wpr)))**	((Dvi,Mst),(Spa,Wpr))	**(Dvi,(Mst,(Spa,Wpr)))**	**(Dvi,(Mst,(Spa,Wpr)))**
E	**(Bap,(Emc,Lma))**	(Emc,(Bap,Lma))	**(Bap,(Emc,Lma))**	(Emc,(Bap,Lma))
F	**(Nco,((Tsu,Apa),^a^))**	(Nco,(Tsu,(Apa,^a^)))	**(Nco,((Tsu,Apa),^a^))**	**(Nco,((Tsu,Apa),^a^))**

(A) Anfo: Angiopteris fokiensis, Pnu: Psilotum nudum, Ovu: Ophioglossum vulgatum, Bja: Botrychium japonicum; (B) Cbi: Cheiropleuria bicuspis, Dpe: Dicranopteris pedata, Vst: Vandenboschia striata; (C) Asfo: Asplenium formosae, Aja: Acystopteris japonica, Dbr: Diplaziopsis brunoniana; (D) Dvi: Diplazium viridescens, Mst: Matteuccia struthiopteris, Spa: Stenochlaena palustris, Wpr: Woodwardia prolifera; (E) Bap: Bolbitis appendiculata, Emc: Elaphoglossum mcclurei, Lma: Lomagramma matthewii; (F) Nco: Nephrolepis cordifolia, Tsu: Tectaria subpedata, Apa: Arthropteris palisotii.

^a^Indicates other sampled species within this lineage. Topologies consistent with the one yielded from coalescent-based methods and nucleotide sequences are shown in bold.

### Reconstruction of the evolutionary history of sporangial annulus

**Figure 4:**
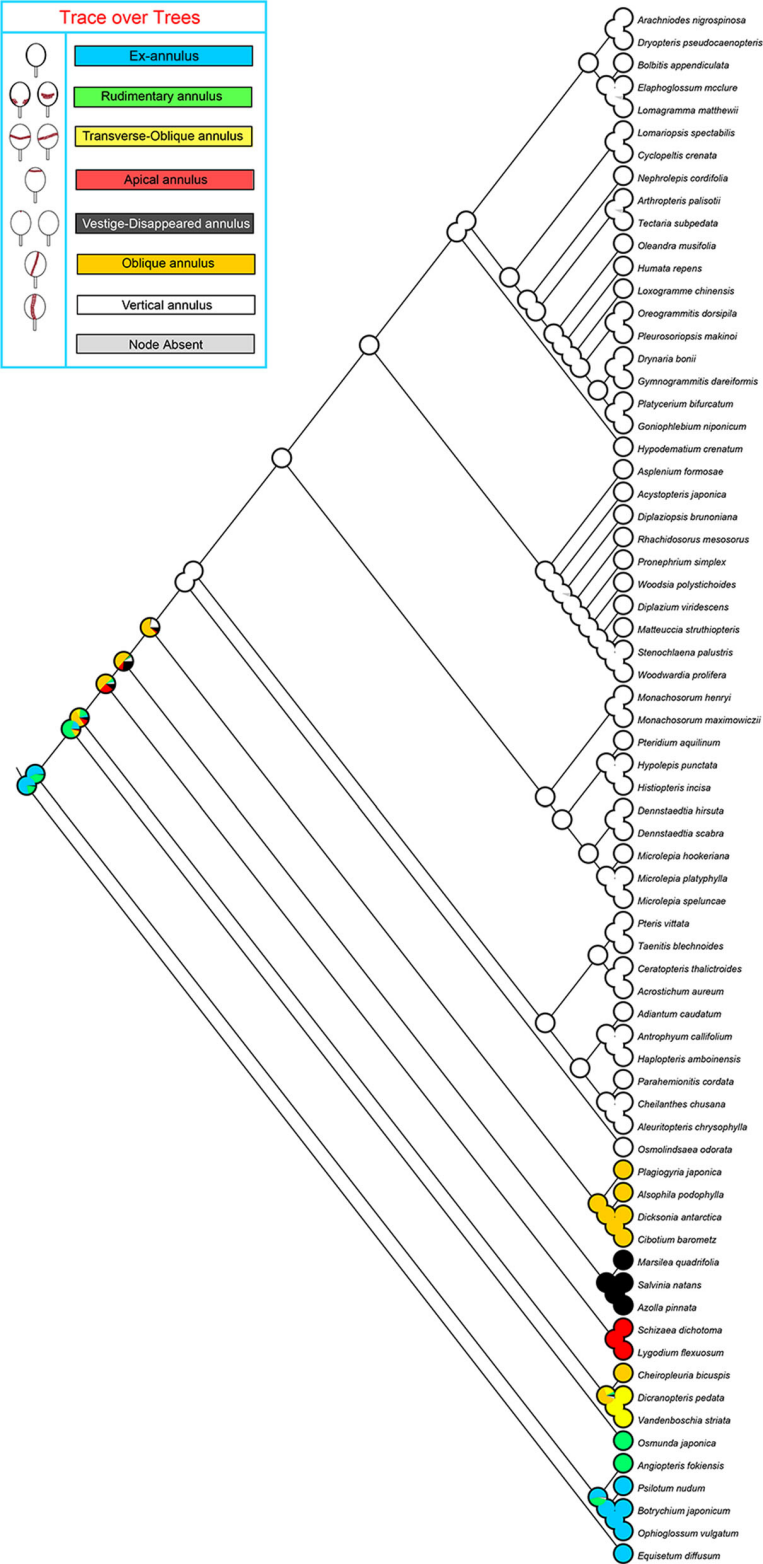
Reconstruction of the evolutionary history of sporangial annulus in ferns. Sampled species with 7 types of sporangial annulus are shown in different colours. For each ancient node, percentage of character state of sporangial annulus is shown.

Our reconstruction of the evolution of sporangial annulus (Fig. 4) showed that ex-annulus sporangia are inferred to be the ancestral state (proportional likelihood [PL] = 1), and the rest of annulus states are likely derived from ex-annulus sporangia. Vertical annulus is suggested as synapomorphy for all polypod ferns (PL > 0.99). Both oblique annulus and rudimentary annulus have experienced parallel evolution.

## Discussion

### Comparison of topologies estimated by various methods

By comparing topologies estimated by coalescent-based and concatenation-based methods using both nucleotide and amino acid sequences (Table 2), we found that the topologies yielded from coalescent-based and concatenation-based methods using nucleotide sequences are mostly consistent, except for the position of *Angiopteris fokiensis.* Topologies yielded from coalescent-based methods using nucleotide sequences and amino acid sequences showed 3 positions of inconsistency, all of which belong to eupolypods. As eupolypods have experienced rapid evolutionary radiation in Cenozoic [7] and nucleotide sequences usually provide more information to reconstruct relationships at a shallow phylogenetic scale, we consider the topology yielded from nucleotide sequences to be more reliable. However, the inconsistent positions among topologies often show relatively lower supporting values, and are often the controversial nodes from past studies based on different genes; we suggest that such inconsistency might be caused partially by ILS and reticulate evolution.

### Relationships of eusporangiate ferns

Which clade is sister to the remaining taxa in ferns is a long-debated question (Fig. 1). Our results strongly supported that Equisetales (horsetails) are the sister group to all other monilophytes. This topology confirmed the results reported by Rai and Graham [12] and Kuo et al. [33] based on plastid genes, and it was accepted by the PPG I [3] in 2016. Distinct from most fern phylogeny based on molecular evidence (Fig. 1), our results based on a coalescent method revealed that Psilotales (whisk ferns), Ophioglossales (moonworts), and Marattiales (king ferns) form a monophyletic clade as ([Psilotales, Ophioglossales], Marattiales), which is sister to leptosporangiate ferns. The monophyletic origin of Psilotales, Ophioglossales, and Marattiales, which belong to eusporangiate ferns, is supported by the structure of sporangia. Being different from the leptosporangiate type, sporangia of eusporangiate ferns have no sporangiophore; they are thick in wall and large in volume, produce large amounts of spores, and have no sporangial annulus or only have a few enlarged parenchyma cells. The incongruence between the results based on coalescent and concatenation methods may be caused by strong ILS effect, which is a main pitfall when using the concatenation method [21].

### Relationship of early leptosporangiates

Within early leptosporangiates, our results revealed a new monophyletic clade in which Gleicheniaceae (forking ferns) is sister to Hymenophyllaceae (filmy ferns), which is different from the mainstream [3, 10, 12–14, 34]. Similar but still different from the topology ([Dipteridaceae, Matoniaceae], Gleicheniaceae], Hymenophyllaceae) reported by Pryer et al. in 2004 [5], in our results, *Cheiropleuria*, which belongs to Dipteridaceae and was formerly placed in Gleicheniales [2, 5, 12, 26, 35, 36], is sister to the monophyletic clade of (Gleicheniaceae, Hymenophyllaceae).

This new relationship is supported by sporangia character. Early leptosporangiates [36] are characterized by diverse sporangia and annulus. However, both Gleicheniaceae and Hymenophyllaceae have spherical sporangia with transverse-oblique annulus, as well as a short sporangial stalk connecting to a prominent receptacle [37]. On the other hand, flattened sporangia with slightly oblique annulus are found in *Cheiropleuria.* Moreover, long sporangial stalk and inapparent receptacle are common in *Cheiropleuria, Dipteris*, and *Matonia*. We suggest that Dipteridaceae, probably together with its sister lineage Matoniaceae [5, 12], may be sister to the clade of (Gleicheniaceae, Hymenophyllaceae). According to our results, Gleicheniales, which is comprised of Dipteridaceae, Matoniaceae, and Gleicheniaceae [26], is no longer a monophyletic lineage, but a paraphyletic one.

### Relationships within polypod ferns

Polypods include more than 80% of living ferns, and their phylogeny remains somewhat controversial and elusive [26, 35, 36]. Our results strongly supported that Dennstaedtiaceae instead of Pteridaceae is sister to eupolypods. This pattern confirmed the topology suggested recently by Rothfels et al. based on 25 low-copy nuclear genes [14] and Lu et al. based on plastid genes [13], as well as the PPG I system [3]. According to our results, the relationships of Pteridaceae [34, 36, 38] and Dennstaedtiaceae [36] are also well resolved. Notably, *Monachosorum* is sister to the rest of the members in Dennstaedtiaceae, rather than being sister to the lineage of Pteridium, Hypolepis, and Histiopteris [36].

Our results showed that eupolypods are divided into 2 major lineages, eupolypods I and eupolypods II, in agreement with the consensus opinion [3]. Within eupolypods II, our results supported that Aspleniaceae is the sister group to the rest of the members, which is different from the current viewpoint [26, 36, 39]. Within eupolypods I, our result strongly supported that Lomariopsidaceae and Nephrolepidaceae form a paraphyletic group, rather than a monophyletic clade based on plastid genes [10, 26, 36].

Our new topology confirmed the morphology-based hypothesis that Dennstaedtiaceae with 2 indusial, rather than Pteridaceae with 1 false indusium, is more closely related to eupolypod ferns [40]. In Pteridaceae, the unstable structure of spherical sporangia, including variable annulus and short sporangial stalk, indicates that these characters of sporangia are relatively original and are close to those with oblique annulus in early leptosporangiates [23]. We also noticed that the characters of spherical sporangia with slightly oblique annulus in *Monachosorum* should be more ancestral than the flattened sporangia with typical vertical annulus in other genera of Dennstaedtiaceae. For distinguishing eupolypods I and eupolypods II, the number and shape of the vascular bundles at the base of petiole have been demonstrated to be of a powerful diagnostic character [36, 39].

### The evolution of sporangial annulus in ferns

By observing the character of sporangial annulus of abundant samples in each fern group and combining these characters with our well-resolved backbone phylogeny (Fig. 3), we reconstructed the evolutionary history of sporangial annulus in ferns (Fig. 4). According to the results, we infer that ex-annulus sporangia, as in Equisetaceae, Psilotaceae, and Ophioglossaceae, is the ancestral state in ferns; rudimentary multiseriate annulus, which is inverse U-shaped in Marattiaceae and U-shaped in Osmundaceae; equatorial transverse-oblique uniseriate annulus, as in Gleicheniaceae and Hymenophyllaceae; oblique annulus as in Cyatheales (tree ferns); and vertical annulus as synapomorphy in polypods have been derived from the ex-annulus state. Both apical annulus, as in Lygodium and Schizaea, and vestige or disappeared annulus, as in Salviniales (aquatic ferns), are likely to be specialized in parallel from oblique annulus. Inconsistent with Bower's hypothesis [23], our results showed that sporangia with apical annulus as in Schizaeales are no longer the ancestral type in ferns but a specialized one. Correspondingly, the oldest fossils of Schizaeaceae are now believed to appear in the Jurassic period (201–145 MY BP) rather than formerly thought Carboniferous period (359–252 MY BP) [41].

## Conclusion

Our results confirmed that Equisetales is sister to all the other monilophytes and that Dennstaedtiaceae is sister to eupolypods, which have been reported previously. Moreover, our results revealed some new relationships, such as that eusporangiate ferns, except Equisetales, may form a monophyletic clade as ([Psilotaceae, Ophioglossaceae], Marattiaceae), while Gleicheniaceae and Hymenophyllaceae form a monophyletic clade, which is sister to Dipteridaceae, and that Aspleniaceae is sister to the rest of the groups in eupolypods II. Most of these results are supported by sporangia characters, and a new evolutionary route of sporangial annulus in ferns is suggested.

### Potential implications

Here, we present a robust fern phylogeny yielded from a large-scale phylogenomic analysis based on a high-quality RNA-seq dataset covering 69 fern species. This backbone phylogeny in ferns sets a foundation for further studies in biology and evolution in ferns and therefore in plants, especially when fern genomes are not available.

## Methods

### *De novo* transcriptome assembly

For each paired-end library, we first removed the Illumina adapter of raw reads using Scythe (Scythe, RRID: SCR_011844) [42] and trimmed the poor-quality bases using DynamicTrim Perl script of the SolexQA package with default parameters [43]. Next, *de novo* transcriptome assembly of each species was conducted using the Trinity package, version trinityrnaseq_r20140413 (Trinity, RRID: SCR_013048) with default parameters [44]. To discard the duplicated sequences, the obtained contigs were clustered using CD-HIT-EST v4.6.1 (CD-HIT, RRID: SCR_007105) to generate nonredundant contigs. All contigs longer than 200 bp in length were used for downstream analysis. We used TransDescoder, a program in the Trinity package, to identify the candidate coding sequences (CDS) from the contigs with default criteria. Finally, the translated protein sequences of CDS were searched by BLASTP against the nonredundant protein database in NCBI with an e-value threshold of 1e-5. These BLASTP hit sequences were used for further analysis.

### Orthology assignment, alignment, and alignment masking

For orthology assignment for the 69 sample assemblies together with the 4 outgroup species, a phylogenetic-based clustering method described previously [16] was used. In short, an all-vs-all BLAST search of amino acid sequence was performed across different species; the BLAST results were clustered using MCL [45] software with the parameters ‘-I 2–tf ^΄^gq(20)^΄^.’ Optimization of the inflation parameter (I) was conducted as described previously [46], and the default value 2.0 was selected ultimately. As the *de novo* assembly by Trinity produces many sequences with high similarity, which contain both paralogs and isoforms [47], when a clustered gene family contains too many sequences (e.g., more than 10), the risk of contamination of isoforms rises, along with the computational infeasibility. Hence, when a species had more than 10 sequences in a gene family, we removed all sequences in this gene family of this species. Then, groups with at least 35 (50%) fern species were aligned using the einsi command, implemented in MAFFT (MAFFT, RRID: SCR_011811) [48], and trimmed by Gblocks with default parameters [49]. Next, for each group, a homologous gene tree was built with RAxML software, version 8.0.20 (RAxML, RRID: SCR_006086), by implementing the maximum likelihood method (ML) [50]. To infer orthologous genes, we used treeprune in the Agalma package [51] to mask the monophyletic sequences. We pruned the paralogous subtrees from the homologous gene trees until only 1 monophyletic subtree was retained. Next, the resulting orthologous gene trees were further filtered by the criteria that each species should be represented by only 1 sequence, and the resulting subset genes were referred to “1-to-1 orthologs,” which were largely free of gene duplication. Then, we extracted both the CDS (nucleotide sequence) and translated amino acid sequence from each orthologous gene group, followed by aligning with MAFFT and trimming with Gblocks. The alignment with coding and corresponding translated sequences longer than 150 bp (or 50 amino acids) in length were kept for further analysis.

### Basic Universal Single Copy Orthologs analysis

The Basic Universal Single Copy Orthologs (BUSCO, RRID: SCR_015008), which employs a core set of orthologs conservative in eukaryotic species to determine the gene coverage of each assembly [52], was employed to assess the completeness of the transcriptome assembly we obtained (Table S2) [53]. A total of 303 BUSCOs were employed to blast against by translated amino acid of the assemblies using BLASTP. Then the numbers of complete and partially matched genes from each assembly were counted. Out of the 69 samples in total, the gene coverage of 65 samples (94.2%) exceeded 82%, with at least 251 complete genes identified. Unexpectedly, among our total assemblies, 1 sample (*Aleuritopteris chrysophylla*, named RS_72) presented an extremely low gene coverage degree, in which only 72 (23.8%) complete housekeeping genes were found (Supplementary Table S2). However, when the sample was deleted from the matrix used to construct the backbone of the phylogenetic tree, the topology remained unchanged, indicating that the lower completeness in this sample did not affect our results (data not shown).

### Phylogenetic analysis

The coalescent-based species trees were reconstructed by ASTRAL v4.10.4 [54], carried out by 100 replicates of multilocus bootstrapping [55]. Each gene tree was constructed with the PROTCATJTTF model by RAxML v8.2.4 (RAxML, RRID: SCR_006086) [50], performed using 100 random replicates to calculate bootstrap value. For the concatenation analysis, we preformed the ML for each matrix using RAxML software (version 8.0.20). Branch support was evaluated using 100 bootstrap replicates. We used the “GTR + Γ4 + I” model for DNA matrices, and the JTTF model for the corresponding protein matrices, selected by “ProtienModelselection.pl” [56]. To estimate the divergence times, we used the concatenated alignment of orthologs, calibrated with the ages of 2 fossils (*Archaeocalamites Senftenbergia*: 354 MY, *Grammatopteris*: 280 MY) [6, 57] as the minimum ages of monilophytes and leptosporangiate ferns, respectively, and a maximum age constraint of 500 MY for land plants in a Bayesian relaxed clock method using MCMCTREE [58] on the coalescent-based species tree.

### Reconstruction of the evolution of sporangial annulus

Characters of sporangial annulus of the sampled species were observed using a polarized light microscope (Axio Scope.A1, ZEISS) after the fresh and mature sporangia were treated with sodium hypochlorite (NaClO) solution. The evolution of sporangial annulus was reconstructed with the likelihood method, implemented in Mesquite v2.7.5 [59]. All character states (i.e., vertical annulus, oblique annulus, rudimentary annulus, ex-annulus, apical annulus, transverse annulus, and vestigial annulus) were treated as unordered and equally weighted. To reconstruct character evolution, a maximum likelihood approach using Markov k-state 1 parameter model [60] was applied. To account for phylogenetic uncertainty, the “Trace-characters-over-trees” command was used to calculate the ancestral states at each node, including probabilities in the context of likelihood reconstructions. To carry out these analyses, characters were plotted onto 100 trees that were sampled in the ML analyses of the combined dataset using RAxML v7. The results were finally summarized as percentage of changes of character states on a given branch among all 100 trees utilizing the option of “Average-frequencies-across-trees.”

## 

### Availability of data and materials

Raw reads of RNA-Seq for 69 fern species were deposited in GenBank under Bioproject accession number PRJNA281136. Transcriptome datasets, alignments, phylogenetic trees, BUSCO results and other supporting data are available via the *GigaScience* repository, *Giga*DB [61].

### Abbreviations

BUSCOs: Basic Universal Single-Copy Orthologs; ILS: incomplete lineage sorting; ML: maximum likelihood; MY: million years; PPG: Pteridophyte Phylogeny Group; RNA-Seq: transcriptome sequencing.

### Additional files

Additional file 1: Tables S1 and S2 and Figures S1–S3.

### Competing interests

The authors declare that they have no competing interests.

### Funding

This work was funded by Shanghai Landscaping and City Appearance Administrative Bureau of China, Scientific Research Grants (G142433, G152420, and F112422), and the National Natural Science Foundation of China (31370234).

### Author contributions

Y.H.Y., H. Shen, and D.M.J. conceived and designed the study. M.L., J.P.S., D.M.J., R.W., and L.L. implemented the data analyses. Y.H.Y., H. Shen, H.J.W., X.L.Z., H. Shang, and Y.F.G. collected the specimens. H. Shen, R.Z., and Y.F.G. prepared the specimens for sequencing. X.L.Z. provided the anatomical data. D.M.J., H. Shen, Y.H.Y., J.P.S., M.L., R.W., H. Shang, X.L.Z., and X.C.Z. interpreted the results and wrote the manuscript.

## Supplementary Material

GIGA-D-17-00169_Original-Submission.pdf

GIGA-D-17-00169_Revision-1.pdf

GIGA-D-17-00169_Revision-2.pdf

Response-to-Reviewer-Comments_Original-Submission.pdf

Response-to-Reviewer-Comments_Revision-1.pdf

Reviewer-1-Report-(Original-Submission) -- Fay-Wei Li 31 Jul 2017 Reviewed

Reviewer-1-Report-(Revision-1) -- Fay-Wei Li 06 Sep 2017 Reviewed

Reviewer-2-Report-(Original-Submission) -- Siavash Mirarab 01 Aug 2017 Reviewed

Reviewer-3-Report-(Original-Submission) -- Naim Matasci 03 Aug 2017 Reviewed

Supplement Files
