# Supplementary material for: Large-scale phylogenomic analysis resolves a backbone phylogeny in ferns
Source: Gigascience. 2017 Nov 24;7(2):gix116. doi: 10.1093/gigascience/gix116 (PMC5795342; doi:10.1093/gigascience/gix116)
Supplement: GIGA-D-17-00169_Original-Submission.pdf [file gix116_giga-d-17-00169_original-submission.pdf]

GigaScience
Large scale phylogenomic analysis resolves a backbone phylogeny in ferns
--Manuscript Draft--

**Manuscript Number:**	GIGA-D-17-00169
**Full Title:**	Large scale phylogenomic analysis resolves a backbone phylogeny in ferns
**Article Type:**	Research
**Funding Information:**	Shanghai Landscaping & City Appearance Administrative Bureau of China (F132421) — Proffesor Yue-Hong Yan<br>Shanghai Landscaping & City Appearance Administrative Bureau of China (F112422) — Proffesor Yue-Hong Yan<br>National Natural Science Foundation of China (31370234) — Proffesor Yue-Hong Yan
**Abstract:**	Background: Ferns originated about 360 million years ago is the sister group of seed plants. Despite remarkable progress in our understanding of fern phylogeny, with conflicting molecular evidences and different morphological interpretations, relationships among major fern lineages remain controversial.<br>Results: With the aim to obtain a robust fern phylogeny, we carried large scale phylogenomic analysis using high-quality transcriptome sequencing data which covered 69 fern species from 38 families and 11 orders. Both coalescent-based and concatenation-based methods were applied to both nucleotides and amino acids sequences in species tree estimation. Among the mainly consistent and strongly supported cladograms, coalescent-based method using nucleotides sequence yielded the most robust cladogram.<br>Conclusions: Our result confirmed that Equisetales is sister to the rest of ferns, and Dennstaedtiaceae is sister to eupolypods. Moreover, our result strongly supported some relationships new to the current view of fern phylogeny, including that Psilotaceae and Ophioglossaceae form a monophyletic clade which is sister to Marattiaceae; Gleicheniaceae and Hymenophyllaceae form a monophyletic clade which is sister to Dipteridaceae; and that Aspleniaceae is sister to the rest groups in eupolypods II. These results were interpreted with morphological traits, especially sporangia characters, and a new evolutionary route of sporangia annulus in ferns was suggested. This backbone phylogeny in ferns sets a foundation for further studies in biology and evolution in ferns, and therefore in plants.
**Corresponding Author:**	Yue-Hong Yan, Ph.D<br>Shanghai Chenshan Botanical Garden<br>Shanghai, CHINA
**Corresponding Author Secondary Information:**	
**Corresponding Author's Institution:**	Shanghai Chenshan Botanical Garden
**Corresponding Author's Secondary Institution:**	
**First Author:**	Hui Shen, PhD
**First Author Secondary Information:**	
**Order of Authors:**	Hui Shen, PhD
	Dongmei Jin, PhD
	Jiang-Ping Shu
	Xi-Le Zhou
	Ming Lei, PhD
	Ran Wei, PhD

Powered by Editorial Manager® and ProduXion Manager® from Aries Systems Corporation

	Hui Shang
	Hong-Jin Wei
	Rui Zhang, PhD
	Li Liu
	Yu_feng Gu
	Xian-Chun Zhang, PhD
	Yue-Hong Yan, Ph.D
**Order of Authors Secondary Information:**	
**Opposed Reviewers:**	Hong Ma, PhD<br>Huck Distinguished Research Professor of Plant Molecular Biology, Pennsylvania State University<br>hxm16@psu.edu<br>Potential competitor
	Fay-Wei Li, PhD<br>Professor (Assistant), Cornell University<br>fl329@cornell.edu<br>Potential competitor
**Additional Information:**	
**Question**	**Response**
Are you submitting this manuscript to a special series or article collection?	No
**Experimental design and statistics**<br><br>Full details of the experimental design and statistical methods used should be given in the Methods section, as detailed in our Minimum Standards Reporting Checklist. Information essential to interpreting the data presented should be made available in the figure legends.<br><br>Have you included all the information requested in your manuscript?	Yes
**Resources**<br><br>A description of all resources used, including antibodies, cell lines, animals and software tools, with enough information to allow them to be uniquely identified, should be included in the Methods section. Authors are strongly encouraged to cite Research Resource Identifiers (RRIDs) for antibodies, model organisms and tools, where possible.<br><br>Have you included the information requested as detailed in our Minimum Standards Reporting Checklist?	Yes

**Availability of data and materials**<br><br>All datasets and code on which the conclusions of the paper rely must be either included in your submission or deposited in publicly available repositories (where available and ethically appropriate), referencing such data using a unique identifier in the references and in the “Availability of Data and Materials” section of your manuscript.<br><br>Have you have met the above requirement as detailed in our Minimum Standards Reporting Checklist?	Yes

Powered by Editorial Manager® and ProduXion Manager® from Aries Systems Corporation

Large scale phylogenomic analysis resolves a backbone phylogeny in ferns

Hui Shen[1,2#], Dongmei Jin[1,2#], Jiang-Ping Shu[1,2], Xi-Le Zhou[1,2], Ming Lei[3], Ran Wei[4], Hui Shang[1,2], Hong-Jin Wei[1,2], Rui Zhang[1,2], Li Liu[1,2], Yu-Feng Gu[1,2], Xian-Chun Zhang[3], Yue-Hong Yan[1,2*]

[#] Equal contributors

*Corresponding author: yhyan@sibs.ac.cn

[1]Shanghai Chenshan Plant Science Research Center, Chinese Academy of Sciences, Shanghai 201602, China; [2]Shanghai Key Laboratory of Plant Functional Genomics and Resources, Shanghai Chenshan Botanical Garden, Shanghai 201602, China; [3]Majorbio Bioinformatics Research Institute, Shanghai 201320, China. [4]State Key Laboratory of Systematic and Evolutionary Botany, Institute of Botany, Chinese Academy of Sciences, Beijing 100093, China.

Abstract

Background: Ferns, originated about 360 million years ago, are the sister group of seed plants. Despite the remarkable progress in our understanding of fern phylogeny, with conflicting molecular evidences and different morphological interpretations, relationships among major fern lineages remain controversial.

Results: With the aim to obtain a robust fern phylogeny, we carried a large scale phylogenomic analysis using high-quality transcriptome sequencing data which covered 69 fern species from 38 families and 11 orders. Both coalescent-based and

concatenation-based methods were applied to both nucleotides and amino acids sequences in species tree estimation. Among the mainly consistent and strongly supported cladograms, coalescent-based method using nucleotides sequence yielded the most robust cladogram.

Conclusions: Our result confirmed that Equisetales is sister to the rest of ferns, and Dennstaedtiaceae is sister to eupolypods. Moreover, our result strongly supported some relationships new to the current view of fern phylogeny, including that Psilotaceae and Ophioglossaceae form a monophyletic clade which is sister to Marattiaceae; Gleicheniaceae and Hymenophyllaceae form a monophyletic clade which is sister to Dipteridaceae; and that Aspleniaceae is sister to the rest groups in eupolypods II. These results were interpreted with morphological traits, especially sporangia characters, and a new evolutionary route of sporangia annulus in ferns was suggested. This backbone phylogeny in ferns sets a foundation for further studies in biology and evolution in ferns, and therefore in plants.

Key Words: phylogenomic, monilophytes, evolution, sporangium, transcriptome

Background

Phylogeny, which reflects natural history, is fundamental to understanding evolution and biodiversity. Ferns (monilophytes), originated about 360 million years (MY) ago, are the sister group of seed plants [1, 2]. With estimated 10,578 extant living species globally [3], they are the second most diverse group in vascular plants. Phylogenetic studies for ferns, especially based on molecular evidences, have been widely carried in recent decades. These studies have revolutionized our understanding of the

evolution in ferns, among which the milestones being setting ferns as the sister group of seed plants [1, 2], placing Psilotaceae and Equisetaceae within ferns [2, 4, 5], and revealing a major polypods radiation following the rise of angiosperms [6, 7]. Resolution at shallow phylogenetic depth among families or genera have also been improved remarkably [8-14].

However, previous researches on fern phylogeny have mostly relied on plastid genes [10, 12, 13], some combined with a few nuclear genes [4, 5, 14] or morphological traits [5, 11]. Due to incomplete lineage sorting (ILS), genes from different resources often show conflicting evolutionary patterns, especially when based on a limited number of samples, some deep relationships in fern phylogeny remain controversial (Figure 1). In the latest PPG I system [3], which has derived from many recent phylogenetic studies, some important nodes remain uncertain, such as (i) what are the relationships among Marattiales, Ophiglossales and Psilotales? (ii) are Hymenophyllales and Gleicheniales sister groups? and (iii) what are the relationships among families in eupolypods II?

Transcriptome sequencing (RNA-Seq) represents massive transcript information from the genome. Phylogenetic reconstructions basing on RNA-Seq are more efficient and cost-effective than traditional PCR-based or EST-based methods when lacking whole-genome data [15]. Successful cases in recent years include mollusks [16], insects [17], the grape family [18], angiosperms [19], and land plants including six ferns [20]. Here, with the aim to reconstruct the framework of fern phylogeny, we

sampled abundant fern species representing all important linages and applied latest phylogenomic analysis basing on RNA-Seq.

To reconstruct a robust and well-resolved phylogeny in ferns, applying multiple methods of phylogenomic analysis is extremely important. Since concatenation-based estimations of species tree usually have good accuracy under low level of ILS, while coalescent-based methods are developed to overcome the effect of ILS, but are sensitive to gene tree estimation error [21], so both concatenation-based and coalescent-based estimations are applied. Moreover, due to the fact that amino-acid sequence is more conserved than nucleotide sequence, it may be more suited to estimate relationships among distant taxa. While for close related taxa, the higher variability of nucleotide sequence brings useful information to reconstruct relationships that might not be differentiated using amino-acid sequence. Therefore, both nucleotide and amino-acid sequences are used in phylogeny reconstruction.

In the aspect of morphology, fern sporangium is an organ for enclosing and dispersing spores, most of which functions like a unique catapult with annulus [22]. During the last centuries, Bower's hypothesis on the evolution of sporangia with a focus on annulus [23] had been one of the most important cornerstones to fern phylogeny based on morphology [24, 25]. However, this hypothesis has been challenged by somewhat conflicting frameworks of fern phylogeny [4, 10, 12, 14, 26]. A robust framework in fern phylogeny which reflects the evolutionary history will improve our understanding for the evolution of fern sporangia as well as other characters.

Data description

Taxa sampling and RNA-Seq

We chose 69 fern species from 38 families according to PPG I system (totally 48 fern families), covering all the 11 orders (Equisetales, Psilotales, Ophioglossales, Marattiales, Osmundales, Hymenophyllales, Gleicheniales, Schezaeles, Salviniales, Cyatheales, and Polypodiales). Information about the location and time for sampling is given in Table S1. All the sampled species were collected under the permissions of the natural reserves and Shanghai Chenshan Botanical Garden in China.

Sporophyll or/and trophophyll were collected and frozen in liquid nitrogen immediately, and preserved in Ultra-low temperature refrigerator at -80°C before RNA extraction. Total RNA was extracted using TRIzol (Life Technologies Corp.) according to the manufacturer's protocols. The RNA concentration was determined using a NanoDrop spectrophotometer, and RNA quality was assessed with an Agilent Bioanalyzer. Paired-end reads were generated by Majorbio Company (Shanghai, China) using the HiSeq 2500 system. Raw reads were deposited in GenBank [27] .

Transcriptomes assembly and orthology assignment

Transcriptomes data were generated from 69 fern species (Table 1). After filtration, about 2,726.9 million pair-end DNA sequence reads (about 313 Gbp) were retained. We assembled these reads *de novo* and obtained a total of 5,449,842 contigs [28].

In order to obtain a reliable phylogenetic relationship, we selected four species as the outgroup, representing the main lineage of land plants: *Amborella trichopoda* (representing angiosperms), *Picea abies* (representing gymnosperms), *Selaginella moellendorffii* (representing lycophytes), *Physcomitrella patens* (representing bryophytes). The translated ORF (protein) sequences of these four species were

downloaded from Phytozone [29] and used in the following analysis.

To ensure the consistency of phylogenomic analysis, we used a phylogenetic-based ortholog selection method, and obtained two subsets of " one to one" orthologous genes that differed in gene number and species occupancy rate, named "Matrix 1" and "Matrix 2" [30]. Matrix 1 consists of 2391 genes that are present in at least 52 taxa (that is 75% of the 69 taxa in total), resulted in 2,024,565 nucleotide and 674,855 amino acid positions, the gene and character occupancy were 88% and 85% respectively. Matrix 2 consists of 1334 genes that are present in at least 62 taxa (that is 90% of the 69 taxa in total), resulted in 1,171,332 nucleotide and 390,444 amino acid positions, the gene and character occupancy reached 94% and 90% in each. For each orthologues gene set, coalescent-based and concatenation-based methods were applied separately to both nucleotides and amino acids sequences. A working flow diagram showing the major processes in this study is given in Figure 2.

Results

Species tree estimated in 69 ferns

For each combination of estimation method (coalescent-based or concatenation-based) and sequence type (nucleotides or amino acids), the cladograms were identical between two results using Matrix 1 and Matrix 2 [31, 32]. In general, the four cladograms (Figure 3, Figure S1, S2, S3) yielded from combinations of method and sequence type are consistent except six sites (Table 2). Among the cladograms, the one estimated by applying coalescent-based method to nucleotide sequences (Figure 3) is the most agreed.

Reconstruction of the evolution history of sporangia annulus

Our reconstruction of the evolution of sporangia annulus showed that ex-annulus sporangia are inferred to be the ancestral state (proportional likelihood [PL]: 1), and the rest of annulus states are likely derived from ex-annulus sporangia. Vertical annulus is suggested as synapomorphy for all polypod ferns (PL > 0.99). Both oblique annulus and rudimentary annulus have experienced parallel evolution.

Discussion

Comparison of cladograms estimated by various methods

By comparing cladograms estimated by coalescent-based and concatenation-based method using both nucleotide and amino-acid sequences (Table 2), we find that the cladograms yielded from coalescent-based and concatenation-based methods using nucleotide sequence are mostly consistent, except the location of *Angiopteris fokiensis.* Cladograms yielded from coalescent-based method using nucleotide sequence and amino-acid sequence showed three sites of inconsistency, all of which belong to eupolypods. Since eupolypods have experienced rapid evolutionary radiation in Cenozoic (Figure 3), and nucleotide sequences usually provide more information to reconstruct relationships among close related taxa, we consider the cladogram yielded from coalescent-based method using nucleotide sequence maybe more reliable. However, the inconsistent sites among cladograms often show relatively lower supporting values, and they are often controversial nodes among different researches based on different genes, we suggest these different results may be aroused partially by LIS and reticulate evolution.

Relationships of eusporangiate ferns

Which clade is sister to the remaining taxa in ferns is a long-debated question (Figure 1). Our results strongly supported that Equisetales (horsetails) are the sister group to

all other monilophytes. This cladogram confirm the results reported for the first time by Rothfels *et al.* in 2015 basing on 25 low-copy nuclear genes [14], and accepted by the PPG I [3] in 2016. Distinct from most fern phylogeny based on molecular evidences (Figure 1), our results revealed that Psilotales (whisk ferns), Ophioglossales (moonworts), and Marattiales (king ferns) form a monophyletic clade as ((Psilotales, Ophioglossales), Marattiales), which is sister to Leptosporangiate ferns. The monophyletic origin of Psilotales, Ophioglossales, and Marattiales, which belong to eusporangiate ferns, is supported by the structure of sporangia. Being different from the Leptosporangiate type, sporangia of eusporangiate ferns have no sporangiophore, they are thick in wall and large in volume, produce a large amounts of spores, and have no sporangia annulus or only a few thickened cells.

Relationship of early leptosporangiates

Within early leptosporangiates, our results revealed a new monophyletic clade that Gleicheniaceae (forking ferns) is sister to Hymenophyllaceae (filmy ferns), which is different from the view of mainstream [3, 10, 12-14, 33]. Similar but still different from the topology (((Dipteridaceae, Matoniaceae), Gleicheniaceae), Hymenophyllaceae) reported by Pryer *et al.* in 2004 [5], in our results, *Cheiropleuria*, which belongs to Dipteridaceae and formerly placed in Gleicheniales [2, 5, 12, 26, 34, 35], is sister to the monophyletic clade of (Gleicheniaceae, Hymenophyllaceae).

This new relationship is supported by sporangia character. Early leptosporangiates [35] are characterized with diverse sporangia and annulus.

However, both Gleicheniaceae (forking ferns) and Hymenophyllaceae (filmy ferns) have spherical sporangia with transverse-oblique annulus, as well as short sporangial stalk connecting to prominent receptacle [36]. Differently, flattened sporangia with slightly oblique annulus are found in *Cheiropleuria.* Moreover, long sporangial stalk and inapparent receptacle are common in *Cheiropleuria*, *Dipteris* and *Matonia*. We suggest Dipteridaceae, probably together with its sister lineage Matoniaceae [5, 12], may form a sister lineage to the clade of (Gleicheniaceae, Hymenophyllaceae). According to our results, the Gleicheniales order, which is comprised of Dipteridaceae, Matoniaceae, and Gleicheniaceae [26], is no longer a monophyletic lineage, but a paraphyletic one.

Relationships within polypod ferns

Polypods include more than 80% of living ferns, and their phylogeny remains somewhat controversial and elusive [26, 34, 35]. Our results strongly supported that Dennstaedtiaceae instead of Pteridaceae, is sister to eupolypods. This pattern confirmed the topology suggested recently by Rothfels *et. al* basing on 25 low-copy nuclear genes [14] and Lu *et. al* basing on plastid genes [13], as well as PPG I system [3]. In our result, the disputation of inner relationships of Pteridaceae [33, 35, 37] and Dennstaedtiaceae [35] are also well resolved. Notably, *Monachosorum* is sister to the rest members in Dennstaedtiaceae, rather than being sister to the lineage of Peridium, Hypolepis and Histiopteris [35].

Our results showed that eupolypods are divided into two major lineages,

eupolypods I and eupolypods II in agree with the consensus opinion. Within eupolypods II, our results supported that Aspleniaceae is the sister group to the rest members, which is new to the current viewpoints [26, 35, 38]. Within eupolypods I, our result strongly supported that Lomariopsidaceae and Nephrolepidaceae form a paraphyletic group, rather than a monophyletic clade based on plastid genes [10, 26, 35].

Our new phylogram confirmed the morphology-based hypothesis that Dennstaedtiaceae with two indusial, rather than Pteridaceae with one false indusium, is more close to eupolypod ferns [39]. In Pteridaceae, the unstable structure of spherical sporangial, including variable annulus and short sporangial stalk, indicates these sporangial are relatively primitive and are close to the sporangial with oblique annulus in early leptosporangiate [23]. We also noticed that the spherical sporangia with slightly oblique annulus in *Monachosorum* should be more primitive than the flattened sporangia with typical vertical annulate in other genera of Dennstaedtaceae. For distinguishing eupolypods I and eupolypods II, the number and shape of the vascular bundles at the base of petiole have been demonstrated to be a powerful diagnostic character [35, 38].

The evolution of sporangia annulus in ferns

By observing the character of sporangia annulus of abundant samples in each fern group, and reconstructing these characters onto our well-resolved backbone phylogeny (Figure 3), here we reconstructed the evolutionary history of sporangia

annulus in ferns (Figure 4). First, exannulate sporangia, as in Equisetaceae, Psilotaceae, and Ophioglossaceae, is the original type in ferns; followed by rudimentary multiseriate annulus, which is inverse U-shaped in Marattiaceae (a), and U-shaped in Osmundaceae (b); and by equatorial transverse-oblique uniseriate annulus, as in Gleicheniaceae and Hymenophyllaceae. After that, the main route divides into two subroutes, one is towards apical annulus as in Lygodium and Schizaea, followed by vestige or disappeared annulus as in Salviniales (aquatic ferns); the other is towards oblique annulus as in Cyatheales (tree ferns), followed by vertical annulus as in polypods. Inconsistent with Bower's hypothesis [23], our results showed that sporangia with apical annulus as in Schizaeales are no longer the primitive type in ferns but a specialized one. Moreover, the oldest fossils of Schizaeaceae is now believed to appear in Jurassic (201-145 Ma BP) rather than formerly thought Carboniferous (359-252 Ma BP) [40].

Conclusion

Our results confirmed that Equisetales is sister to all the other monilophytes, and Dennstaedtiaceae is sister to eupolypods which have been reported previously. Moreover, our results revealed some new relationships, such as eusporangiate ferns except Equisetales form a monophyletic clade as ((Psilotaceae, Ophioglossaceae), Marattiaceae); while Gleicheniaceae and Hymenophyllaceae form a monophyletic clade which is sister to Dipteridaceae; and Aspleniaceae is sister to the rest groups in eupolypods II. Most of these results are supported by sporangia characters, and a new evolutionary route of sporangia annulus in ferns is suggested.

Potential implications

Here, we present a robust fern phylogeny yielded from a largescale phylogenomic analysis based on a high-quality RNA-seq dataset set covering 69 fern specie. This backbone phylogeny in ferns sets a foundation for further studies in biology and evolution in ferns and therefore in plants, especially when fern genomes are not available.

Methods

De novo transcriptome assembly

For each paired-end library, we first removed the Illumina adapter of raw reads using Scythe (32) and trimmed the poor quality bases using DynamicTrim Perl script of the SolexQA package with default parameters [41]. Next, *de novo* transcriptome assembly of each species was conducted using the Trinity package (version: trinityrnaseq_r20140413) with default parameters [42]. To discard the duplicated sequences, the obtained contigs were clustered using CD-HIT-EST (v4.6.1) to generated a non-redundant contigs. All contigs with lengths greater than 200 bp were used for downstream analysis. We used the transDescoder, a program in the Trinity package, to identify the candidate coding sequences (CDSs) from the contigs with default criteria. Finally, the translated protein sequences of CDSs were searched by BLASTP against the NCBI nr protein database with an e-value threshold of 1E-5. These BLASTP hit sequences were used for further analysis.

Orthology assignment, alignment, and alignment masking

The orthology assignment for the 69 sample assemblies together with the four outgroup species employed a phylogenetic based clustering method described previously [16]. In short, all-vs-all BLAST search of amino acid sequence was performed among every species, the BLAST results were clustered using MCL [43]

software with the parameters ‘-I 2–tf ′gq(20)″. Optimization of the inflation parameter (I) was conducted as described previously [44], the default value 2.0 was selected ultimately. To reduce the complexity of each group, we removed all sequences of the species that had more than 10 sequences in this group. Then, groups with at least 35 (50%) ferns species were aligned using einsi command, implemented in MAFFT [45], and trimmed by Gblocks with default parameters [46]. Next, for each group, homologous gene tree was built with RAxML software (version: 8.0.20) by implementing the maximum likelihood method (ML) [47]. To infer orthologous genes, we used treeprune.pyscript in the agalma [48] package to mask the monophyletic sequences. We pruned the paralogous subtrees from the homologous gene trees until only one monophyletic subtree retained. Next, the resulted orthologous gene trees were further filtered by the criteria that each species should be represented by only one sequence, this resulted subset genes were referred to “one to one orthologs”, which were largely free of gene duplication. Then, we extracted both the CDSs (nucleotide sequence) and translated amino acid sequence from the each orthologous gene group, followed by aligning with MAFFT and trimming with Gblocks. The alignment which with coding and corresponding translated sequences lengths greater than 150 bp (or 50 amino acids) were kept for the further analysis.

BUSCO analysis

The Basic Universal Single Copy Orthologs (BUSCOs), which employ a core set of orthologs conserved in all eukaryotic species to determine the gene coverage degree of each assembly [49], was employed to assess the completeness of the transcriptome assembly we obtained (Table S2) [50]. A total of 303 BUSCOs were employed to blast against by translated amino acid of the assemblies using BLASTP. Then the number of complete and parcially matched gene from each assembly was counted respectively. Out of 69 samples in total, 65 samples (that is 94.2% of total) were defined to have a relatively higher gene coverage degree. In these samples, at

least 251 complete genes (up to 295) could be identified, making the coverage rate exceeded 80%. Unexpectedly, among our total assemblies, 1 sample (*Aleuritopteris chrysophylla*, named RS_72) present extremely low gene coverage degree, in which only 72 (23.8%) complete housekeeping genes could be found (Supplementary Table 2). However, when the sample is deleted from the matrix used to construct the backbone of the phylogenetic tree, the cladogram remains unchanged, indicating that the lower completeness in this sample doesn't affect our results (data not shown).

Phylogenetic analysis

The coalescent-based species tree was reconstructed by ASTRAL v4.10.4 [51], carried out 100 replicates of multi-locus bootstrapping [52]. Statistically consistency was estimated from unrooted gene trees under the multi-species coalescent model. Each gene tree was constructed with the PROJTT model by RAxML v8.2.4 [47], performed 100 random replicates to calculate bootstrap value. For the concatenation analysis, we preformed the maximun likelihood analyses (ML) for each matrix using RAxML softwore (version: 8.0.20). The branch support was evaluated using 100 bootstrap replicates. We used the "GTR + Γ4 + I" model for DNA matrices, and the JTTF model for the corresponding protein matrices, selected by "ProtienModelselection.pl" [53]. To estimate the divergence times, we used the concatenated alignment of orthologs, calibrated with ages of two fossils (*Archaeocalamites Senftenbergia*: 354 MY, Grammatopteris: 280 MY [54] [6]) as the minimum ages of monilophytes and leptosporangiate ferns, respectively, and a maximum-age constraint of 500 MY for land plants, in a Bayesian relaxed clock method using MCMCTREE [55] on the coalescent species tree.

Reconstruction of the evolution of sporangia annulus

Characters of sporangia annulus of the sampled species were observed using a polarized light microscope (Axio Scope.A1, ZEISS) after the fresh and mature

sporangia were treated with sodium hypochlorite (NaClO) solution. The evolution of sporangia annulus was reconstructed with likelihood method implemented in Mesquite v2.7.5 [56]. All character states (i.e., vertical annulus, oblique annulus, rudimentary annulus, ex-annulus, apical annulus, transverse annulus, and vestigial annulus) were treated as unordered and equally weighted. To reconstruct character evolution, a maximum likelihood approach using Markov k-state 1 parameter model [57] was applied. To account for phylogenetic uncertainty, the “Trace-characters-over-trees” command was used to calculate ancestral states at each node including probabilities in the context of likelihood reconstructions. To carry out these analyses, characters were plotted onto 100 trees that were sampled in the ML analyses of the combined dataset using RAxML v7. The results were finally summarized as percentage of changes of character states on a given branch among all 100 trees utilizing the option of “Average-frequencies-across-trees”.

Declarations

List of abbreviations

BUSCOs, the basic universal single copy orthologs;

ILS, incomplete lineage sorting;

MY, million years;

PPG, the pteridophyte phylogeny group;

RNA-Seq, transcriptome sequencing.

Additional files

Additional file1: Tables S1 to S2 and Figures S1 to S3.

Availability of data and materials

Raw reads of RNA-Seq for 69 fern species were deposited in GenBank under Bioproject accession number PRJNA281136.

Transcriptome datasets for 69 fern species: https://figshare.com/s/0f773861b6813f97ff63; datasets of coalescent-based species tree: https://figshare.com/s/e5e70c2fd3990e5176d8; Datasets of concatenation based phylogenetic tree: https://figshare.com/s/8af236b660f61078e40b; Alignments: https://figshare.com/s/f835735cb66911ff1ffd; BUSCO results: https://figshare.com/s/bf999173d04b4c311d46; Scripts: https://figshare.com/s/b28085ee6a7b69f758e9.

Consent for publication

Not applicable

Competing interests

The authors declare that they have no competing interests

Funding

This work was funded by Shanghai Landscaping & City Appearance Administrative Bureau of China, Scientific Research Grants (F132421 and F112422) and the National Natural Science Foundation of China (31370234).

Authors' contributions

YHY and HShen conceived of and oversaw the study. YHY, HShen designed, ML, JPS, RW, DMJ and LL implemented the data analyses. YHY, HShen, HJW, XLZ, HShang and YFG collected the specimens. HShen, RZ and YFG prepared the specimens for sequencing. XLZ provides the anatomical data. DMJ, HShen, YHY, JPS, ML, RW, HShang, XLZ and XCZ wrote the manuscript.

Acknowledgements

We thank Dr. Lin-Bin Zhang, Prof. Yin-long Qiu and Paul G. Wolf from the USA and Dr. Jun Yang from Shanghai Chenshan Botanical Garden of China for providing important suggestions regarding the research methods. We appreciate Prof. Xiao-Ya Chen, Yong-Hong Hu, Jin-shuang Ma, and Zhao-qin Chu from Shanghai Chenshan Botanical Garden of China, as well as Prof. Fu-Wu Xing from South China Botanical Garden of CAS for helpful comments and suggestions. We also thank reviewers of the former manuscript Dr. Fay-Wei Li, Siavash Mirarab, and Naim Matasci for their helpful suggestions.

Ethics approval and consent to participate

Not applicable

References

1. Duff RJ, Nickrent DL. Phylogenetic relationships of land plants using mitochondrial small-subunit rDNA sequences. Am J Bot, 1999;86:372-86.

2. Pryer KM, Schneider H, Smith AR, Cranfill R, Wolf PG, Hunt JS, et al. Horsetails and ferns are a monophyletic group and the closest living relatives to seed plants. Nature. 2001;409:618-22.

3. The Pteridophyte Phylogeny Group. A community-derived classification for extant lycophytes and ferns. J Syst Evol. 2016. doi:10.1111/jse.12229

4. Qiu Y-L, Li L, Wang B, Chen Z, Knoop V, Groth-Malonek M, et al. The deepest divergences in land plants inferred from phylogenomic evidence. Proc Natl Acad Sci USA. 2006;103:15511-6.

5. Pryer KM, Schuettpelz E, Wolf PG, Schneider H, Smith AR, Cranfill R. Phylogeny and evolution of ferns (monilophytes) with a focus on the early leptosporangiate divergences. Am J Bot. 2004;91:1582-98.

6. Schneider H, Schuettpelz E, Pryer KM, Cranfill R, Magallon S, Lupia R. Ferns diversified in the shadow of angiosperms. Nature. 2004;428:553-7.

7. Schuettpelz E, Pryer KM. Evidence for a Cenozoic radiation of ferns in an angiosperm-dominated canopy. Proc Natl Acad Sci USA. 2009;106:11200-5.

8. Zhang LB, Zhang L, Dong SY, Sessa EB, Gao XF, Ebihara A. Molecular circumscription and major evolutionary lineages of the fern genus Dryopteris (Dryopteridaceae). BMC Evol Biol. 2012. doi:10.1186/1471-2148-12-180.

9. Liu H-M, Zhang X-C, Wang W, Qiu Y-L, Chen Z-D. Molecular phylogeny of the fern family dryopteridaceae inferred from chloroplast rbcL and atpB genes. Int J Plant Sci. 2007;168:1311-23.

10. Liu H-M. Embracing the pteridophyte classification of Ren-Chang Ching using a generic phylogeny of Chinese ferns and lycophytes. J Syst Evol. 2016;54:307-35.

11. Schneider H, Smith AR, Pryer KM. Is morphology really at odds with molecules in estimating fern phylogeny? Syst Bot. 2009;34:455-75.

12. Rai HS, Graham SW. Utility of a large, multigene plastid data set in inferring higher-order relationships in ferns and relatives (monilophytes). Am J Bot. 2010;97:1444-56.

13. Lu J-M, Zhang N, Du X-Y, Wen J, Li D-Z. Chloroplast phylogenomics resolves key relationships in ferns. J Syst Evol. 2015;53:448-57.

14. Rothfels CJ, Li F-W, Sigel EM, Huiet L, Larsson A, Burge DO, et al. The evolutionary history of ferns inferred from 25 low-copy nuclear genes. Am J Bot. 2015;102:1089-107.

15. Hittinger CT, Johnston M, Tossberg JT, Rokas A. Leveraging skewed transcript abundance by RNA-Seq to increase the genomic depth of the tree of life. Proc Natl Acad Sci USA. 2010;107:1476-81.

16. Smith S, Wilson N, Goetz F, Feehery C, Andrade S, Rouse G, et al. Resolving the evolutionary relationships of molluscs with phylogenomic tools. Nature. 2011;480:364-7.

17. Misof B, Liu S, Meusemann K, Peters RS, Donath A, Mayer C, et al. Phylogenomics resolves the timing and pattern of insect evolution. Science. 2014;346:763-7.

18. Wen J, Xiong Z, Nie Z-L, Mao L, Zhu Y, Kan X-Z, et al. Transcriptome sequences resolve deep relationships of the grape family. Plos One. 2013;8:e74394.

19. Zeng L, Zhang Q, Sun R, Kong H, Zhang N, Ma H. Resolution of deep angiosperm phylogeny using conserved nuclear genes and estimates of early divergence times. Nature Communications. 2014. doi:10.1038/ncomms5956

20. Wickett NJ, Mirarab S, Nam N, Warnow T, Carpenter E, Matasci N, et al. Phylotranscriptomic analysis of the origin and early diversification of land plants. Proc Natl Acad Sci USA. 2014;111:E4859-68.

21. Mirarab S, Bayzid MS, Boussau B, Warnow T. Statistical binning enables an accurate coalescent-based estimation of the avian tree. Science, 2014.doi:10.1126/science.1250463

22. Noblin X, Rojas N, Westbrook J, Llorens C, Argentina M, Dumais J. The fern sporangium: a unique catapult. Science. 2012;335:1322.

23. Bower FO. The Ferns (Filicales) treated comparatively with a view to their natural classification. Vol 1-3. London: Cambridge University Press. 1923-1928.

24. Pichi-Sermolli REG. Historical review of the higher classification of the Filicopsida. In: Jermy AC, Crabb JA, Thomas BA, editors. Phylogeny and classification of the ferns. London: Bot J Linn Soc. 1973. p.11-40.

25. Smith AR. Non-molecular phylogenetic hypotheses for ferns. Am Fern J. 1995;85(4):104-22.

26. Smith AR, Pryer KM, Schuettpelz E, Korall P, Schneider H, Wolf PG. A classification for extant ferns. Taxon. 2006;55:705-31.

27. Raw reads. https://www.ncbi.nlm.nih.gov/bioproject/?term=PRJNA281136. Accessed 5 July 2017.

28. Transcriptome datasets. https://figshare.com/s/0f773861b6813f97ff63. Acessed 5 July 2017.

29. Phytozone. http://phytozome.jgi.doe.gov/. Accessed 5 July 2017.

30. Alignments. Available from: https://figshare.com/s/f835735cb66911ff1ffd. Accessed 5 July 2017.

31. Datasets of coalescent-based species tree. Available from: https://figshare.com/s/e5e70c2fd3990e5176d8. Accessed 5 July 2017.

32. Datasets of concatenation based phylogenetic tree. Available from: https://figshare.com/s/8af236b660f61078e40b. Accessed 5 July 2017.

33. Schneider H. Evolutionary morphology of ferns (monilophytes). Annu Plant Rev. 2013;45:115-40.

34. Christenhusz MJM, Chase M. Trends and concepts in fern classification. Ann Bot. 2014;113:571-94.

35. Schuettpelz E, Pryer KM. Fern phylogeny inferred from 400 leptosporangiate species and three plastid genes. Taxon. 2007;56:1037-50.

36. Bierhorst DW. Morphology of vascular plants. New York: Macmillan; 1971.

37. Schuettpelz E, Schneider H, Huiet L, Windham MD, Pryer KM. A molecular phylogeny of the fern family Pteridaceae: Assessing overall relationships and the affinities of previously unsampled genera. Mol Phylogenet Evol. 2007;44:1172-85.

38. Rothfels CJ, Sundue MA, Kuo L-Y, Larsson A, Kato M, Schuettpelz E, et al. A revised family-level classification for eupolypod II ferns (Polypodiidae: Polypodiales). Taxon. 2012;61:515-33.

39. Mickel JT. The classification and phylogenetic position of the Dennstadtieaceae. In Jeremy AC, Crabbe JA, Thomas BA, editors. The phylogeny and classification of the ferns. London: Academic Press for The Linnean Society of London. 1973. p. 135-44.

40. Taylor TN, Taylor EL, Krings M. Paleobotany: The biology and evolution of fossil plants. 2nd ed. San Diego: Academic Press. 2009. p. 1141

41. Cox MP, Peterson DA, Biggs PJ. SolexaQA: At-a-glance quality assessment of Illumina second-generation sequencing data. BMC Bioinformatics. 2010. doi:10.1186/1471-2105-11-485.

42. Grabherr MG, Haas BJ, Yassour M, Levin JZ, Thompson DA, Amit I, et al. Full-length transcriptome assembly from RNA-Seq data without a reference genome. Nat Biotechnol. 2011;29:644-U130.

43. van Dongen S. A cluster algorithm for graphs. Technical Report INS-R0010, National Research Institute for Mathematics and Computer Science in the Netherlands. 2000. http://micans.org/mcl/index.html?sec_thesisetc. Accessed 5 July 2017.

44. Hejnol A, Obst M, Stamatakis A, Ott M, Rouse GW, Edgecombe GD, et al. Assessing the root of bilaterian animals with scalable phylogenomic methods. P Roy Soc B-Biol Sci. 2009;276:4261-70.

45. Katoh K, Standley DM. MAFFT Multiple Sequence Alignment Software Version 7: Improvements in Performance and Usability. Mol Biol Evol. 2013;30:772-80.

46. Talavera G, Castresana J. Improvement of phylogenies after removing divergent and ambiguously aligned blocks from protein sequence alignments. Syst Biol. 2007;56:564-77.

47. Stamatakis A. RAxML: a tool for phylogenetic analysis and post-analysis of large phylogenies. Bioinformatics. 2014;30:1312-3.

48. Dunn CW, Howison M, Zapata F. Agalma: an automated phylogenomics workflow. BMC Bioinformatics. 2013;14:330.

49. Simao FA, Waterhouse RM, Ioannidis P, Kriventseva EV, Zdobnov EM. BUSCO: assessing genome assembly and annotation completeness with single-copy orthologs. Bioinformatics. 2015;31:3210-2.

50. BUSCO results. https://figshare.com/s/bf999173d04b4c311d46. Accessed 5 July 2017

51. Mirarab S, Reaz R, Bayzid MS, Zimmermann T, Swenson MS, Warnow T. ASTRAL: genome-scale coalescent-based species tree estimation. Bioinformatics. 2014;30:I541-8.

52. Seo TK. Calculating bootstrap probabilities of phylogeny using multilocus sequence data. Mol Biol Evol. 2008;25:960-71.

53. ProtienModelselection.pl. https://github.com/stamatak/standard-RAxML/. Accessed 5 July 2017.

54. Rößler R, Galtier J. First Grammatopteris tree ferns from the Southern Hemisphere – new insights in the evolution of the Osmundaceae from the Permian of Brazil. Rev Palaeobot Palynol. 2002;121:205-30.

55. dos Reis M, Yang Z. Approximate Likelihood Calculation on a Phylogeny for Bayesian Estimation of Divergence Times. Mol Biol Evol. 2011;28:2161-72.

56. Maddison WP, Maddison DR. Mesquite: a modular system for evolutionary analysis. 2011. http://mesquiteproject.org. Accessed 5 July 2017.

57. Lewis PO, Olmstead R. A Likelihood Approach to Estimating Phylogeny from Discrete Morphological Character Data. Syst Biol. 2001;50:913-925.

Figure legends

Figure 1. Cladograms (a-f) adapted from published results [5, 12-14, 26, 33]. Branches with support < 75% were shown using dotted lines; and taxa which differ in their phylogeny locations were shown in different colors.

Figure 2. A working flow diagram showing the major processes of data production and analysis in this study. Three major processes are De novo transcriptome assembly, one-to-one orthologs prediction, and phylogenetic analysis. The rectangles represent the main results and the ellipses represent the main methods and analysis.

Figure 3. Phylogeny of ferns reconstructed by coalescent-based method using nucleotide sequence with divergence times calculated. Support values for the main phylogeny (a) calculated from Matrix 1/Matrix 2 are listed as percentages; * indicates 100%/100%. Representative leave(s), sporangium and the corresponding lineage are labeled with a same number. Simplified cladogram (b) shows the main linages as in Figure 1. Species in phylogeny (a) and the corresponding lineage in cladogram (b) are shown in a same color.

Figure 4. Reconstructed evolutionary history of sporangia annulus in ferns. Sampled species with seven types of sporangia annulus are shown in different colours. For each ancient node, percentage of character state of sporangia annulus is shown.

Table 1. Sequencing and assembly information of the transcriptome data. The number of ortholog genes used in Matrix 1 and Matrix 2 were shown.

ID	Species	Clean data (G)	Total reads (clean)	Q30%	Number of contigs	N50 (bp)	Mean (bp)	Genes in Matrix 1	Genes in Matrix 2
RS1	*Pronephrium simplex*	4.7	38045864	91.24	151319	887	581.07	2,168	1,254
RS10	*Antrophyum callifolium*	4.0	32745384	91.76	64107	1819	998.73	2,226	1,305
RS101	*Oleandra musifolia*	4.5	36487068	91.45	37075	1493	919.3	2,093	1,248
RS103	*Woodsia polystichoides*	3.9	31465870	90.91	47812	1348	811.3	2,287	1,310
RS107	*Equisetum diffusum*	4.4	35693238	90.21	88932	1154	655.64	1,811	1,254
RS108	*Oreogrammitis dorsipila*	4.6	37037324	90.57	266540	591	485.1	2,141	1,273
RS11	*Vandenboschia striata*	4.8	38639790	90.3	261724	460	422.76	1,959	1,276
RS111	*Pleurosoriopsis makinoi*	4.8	38983796	90.13	98187	1145	632.29	2,182	1,277
RS112	*Azolla pinnata subsp. asiatica*	4.4	35735206	90.57	78295	1348	777.92	1,418	839
RS114	*Taenitis blechnoides*	4.1	32898682	90.98	70495	1262	711.3	2,186	1,278
RS115	*Gymnogrammitis dareiformis*	3.9	31630988	89.81	119483	569	449.38	1,996	1,220
RS116	*Schizaea dichotoma*	4.5	36668734	89.6	67422	1350	826.92	2,035	1,285
RS119	*Botrychium japonicum*	4.8	38603000	90.28	85236	1477	846.97	1,866	1,283
RS122	*Goniophlebium niponicum*	4.8	38786214	90.82	54152	1663	951.92	2,279	1,300
RS123	*Arthropteris palisotii*	4.4	35646740	91	50700	1454	891.67	2,286	1,311
RS124	*Matteuccia struthiopteris*	4.2	34080998	90.44	57514	1345	776.52	2,290	1,313
RS127	*Salvinia natans*	4.2	33780056	91.17	79393	1379	767.14	1,905	1,173
RS128	*Woodwardia prolifera*	5.1	40967322	91.63	69931	1557	859.72	2,328	1,328
RS14	*Diplazium viridescens*	4.0	32320416	90.46	88236	1434	780.87	2,269	1,310
RS16	*Bolbitis appendiculata*	4.7	37503336	91.66	201426	802	556.39	2,226	1,288
RS17	*Dryopteris pseudocaenopteris*	4.1	33136196	91.23	102751	723	514.92	2,236	1,298
RS18	*Dicranopteris pedata*	4.2	33942120	92.04	74011	1193	684.09	2,031	1,304
RS19	*Haplopteris amboinensis*	4.2	42772168	94.17	47603	1713	1041.8	2,249	1,307
RS21	*Psilotum nudum*	8.5	85199034	93.6	66212	1739	927.19	1,741	1,223
RS24	*Cyclopeltis crenata*	4.6	37158058	91.5	29668	600	491.82	2,146	1,279
RS25	*Asplenium formosae*	4.6	46629754	93.5	73318	1722	989.84	2,273	1,312
RS27	*Lomariopsis spectabilis*	4.1	33233594	91.77	98030	1466	750.42	2,225	1,304
RS28	*Cheiropleuria bicuspis*	5.1	41617294	91.35	99411	1435	832.82	2,022	1,295
RS31	*Plagiogyria japonica*	5.7	46472760	91.92	89532	1258	733.9	2,036	1,222
RS34	*Alsophila podophylla*	4.9	48768608	93.43	66254	1580	904.62	2,195	1,289
RS35	*Histiopteris incisa*	4.3	43115390	93.81	61231	1749	985.03	2,319	1,316
RS36	*Pteris vittata*	4.1	41212858	94.37	76666	1868	1021.13	2,296	1,312
RS37	*Cibotium barometz*	4.1	33263550	91.92	85555	1612	891.87	1,790	1,099
RS38	*Osmunda japonica*	4.1	33485274	92.05	58612	1730	901.28	1,732	1,159
RS39	*Loxogramme chinensis*	3.9	31392952	92.16	84796	1065	651.88	2,240	1,305
RS4	*Microlepia hookeriana*	4.0	40561422	94.49	95951	1610	874.06	2,262	1,301
RS41	*Pteridium aquilinum*	4.6	46157134	93.51	55615	1742	960.37	2,321	1,316

RS42	*Hypolepis punctata*	4.4	43828154	93.56	59717	1371	833.68	2,277	1,308
RS43	*Dicksonia antarctica*	3.9	31210608	91.69	56494	1533	902.96	2,045	1,213
RS45	*Rhachidosorus mesosorus*	4.4	35348994	91.98	80069	1541	835.92	2,300	1,315
RS46	*Drynaria bonii*	4.5	36017548	92.02	68132	1077	643.93	2,176	1,279
RS47	*Platycerium bifurcatum*	4.1	33209740	91.62	40456	1097	694.56	2,148	1,283
RS48	*Angiopteris fokiensis*	4.4	35120302	91.12	57637	1629	932.57	1,917	1,306
RS5	*Diplaziopsis brunoniana*	4.3	34698846	91.35	70184	822	541.31	2,040	1,234
RS50	*Dennstaedtia pilosella*	4.5	45618446	93.63	84813	1582	831.56	2,308	1,313
RS51	*Monachosorum henryi*	4.1	41658504	93.42	87832	1465	803.17	2,255	1,288
RS52	*Acystopteris japonica*	5.5	44662146	91.15	57118	1507	873.59	1,222	677
RS53	*Monachosorum maximowiczii*	4.8	48497004	93.58	101448	1817	899.54	2,257	1,294
RS54	*Dennstaedtia scabra*	5.1	51360716	93.47	92158	1565	845.44	1,818	1,056
RS56	*Arachniodes nigrospinosa*	5.1	50929362	94.47	57168	1623	916.1	2,332	1,319
RS69	*Cheilanthes chusana*	5.2	51851066	94.18	49449	1727	1012.63	2,317	1,324
RS7	*Elaphoglossum mcclurei*	4.1	32800248	92.31	57330	1398	846.79	2,267	1,299
RS70	*Lomagramma matthewii*	4.4	35218876	91.21	65170	1748	947.18	2,258	1,307
RS71	*Osmolindsaea odorata*	4.6	46808646	94.13	113778	1521	845.96	2,257	1,312
RS72	*Aleuritopteris chrysophylla*	4.8	47955674	94.18	61637	1669	929.63	2,307	1,322
RS77	*Marsilea quadrifolia*	4.3	34724432	91.76	65227	1607	930.31	2,188	1,299
RS8	*Humata repens*	4.5	36606746	91.17	68932	1267	690.35	2,264	1,315
RS81	*Tectaria subpedata*	4.2	42539482	94.43	57384	1326	797.83	2,128	1,242
RS84	*Ophioglossum vulgatum*	4.4	35637330	91.77	71821	1226	741.62	1,631	1,179
RS85	*Nephrolepis cordifolia*	5.0	40063236	90.81	55207	1530	842.63	2,302	1,319
RS86	*Microlepia platyphylla*	4.6	46324294	94	74956	1763	945.87	2,267	1,295
RS88	*Lygodium flexuosum*	4.2	34098316	91.44	66751	1514	867.82	2,064	1,296
RS89	*Hypodematium crenatum*	4.1	32711798	91.58	52813	1416	852.57	2,298	1,319
RS90	*Acrostichum aureum*	5.4	43422574	90.69	46189	1729	1043.2	2,303	1,319
RS91	*Adiantum caudatum*	5.1	51062204	94.23	51145	1575	950.49	2,323	1,327
RS92	*Parahemionitis cordata*	4.1	33309450	91.72	47508	1456	894.42	2,306	1,317
RS93	*Microlepia speluncae*	4.4	44124842	94.55	94980	1720	917.59	2,292	1,308
RS97	*Stenochlaena palustris*	4.7	37887642	91.81	58416	1655	945.83	2,300	1,316
RS98	*Ceratopteris thalictroides*	3.9	31741082.0	91.4	74728	1610	912.26	2,231	1,296

Table 2. Inconsistent topologies using different methods and sequences.

Site	Coalescent-based method		Concatenation-based method	
	nucleotide	amino-acid	nucleotide	amino-acid
A	**(Anfo,(Pnu,(Ovu,Bja)))**	**(Anfo,(Pnu,(Ovu,Bja)))**	((Pnu,(Ovu,Bja)),(Anfo,#))	((Pnu,(Ovu,Bja)),(Anfo,#))
B	**(Cbi,(Dpe,Vst))**	**(Cbi,(Dpe,Vst))**	**(Cbi,(Dpe,Vst))**	((Dpe,Vst),(Cbi,#))
C	**(Asfo,(Aja,(Dbr,#)))**	**(Asfo,(Aja,(Dbr,#)))**	**(Asfo,(Aja,(Dbr,#)))**	(Asfo,((Aja,Dbr),#))
D	**(Dvi,(Mst,(Spa,Wpr)))**	((Dvi,Mst),(Spa,Wpr))	**(Dvi,(Mst,(Spa,Wpr)))**	**(Dvi,(Mst,(Spa,Wpr)))**
E	**(Bap,(Emc,Lma))**	(Emc,(Bap,Lma))	**(Bap,(Emc,Lma))**	(Emc,(Bap,Lma))
F	**(Nco,((Tsu,Apa),#))**	(Nco,(Tsu,(Apa,#)))	**(Nco,((Tsu,Apa),#))**	**(Nco,((Tsu,Apa),#))**

(A) Anfo: *Angiopteris fokiensis*, Pnu: *Psilotum nudum*, Ovu: *Ophioglossum vulgatum*, Bja: *Botrychium japonicum*; (B) Cbi: Cheiropleuria bicuspis, Dpe: *Dicranopteris pedata*, Vst: *Vandenboschia striata*; (C) Asfo: Asplenium formosae, Aja: *Acystopteris japonica*, Dbr: *Diplaziopsis brunoniana*; (D) Dvi: *Diplazium viridescens*, Mst: *Matteuccia struthiopteris*, Spa: *Stenochlaena palustris*, Wpr: *Woodwardia prolifera*; (E) Bap: *Bolbitis appendiculata*, Emc: Elaphoglossum mcclurei, Lma: *Lomagramma matthewii*; (F) Nco: *Nephrolepis cordifolia*, Tsu: *Tectaria subpedata*, Apa: *Arthropteris palisotii*. # indicates other sampled species within this lineage. Topologies consistent with the one yielded from coalescent-based method and nucleotide sequences are shown in bold.

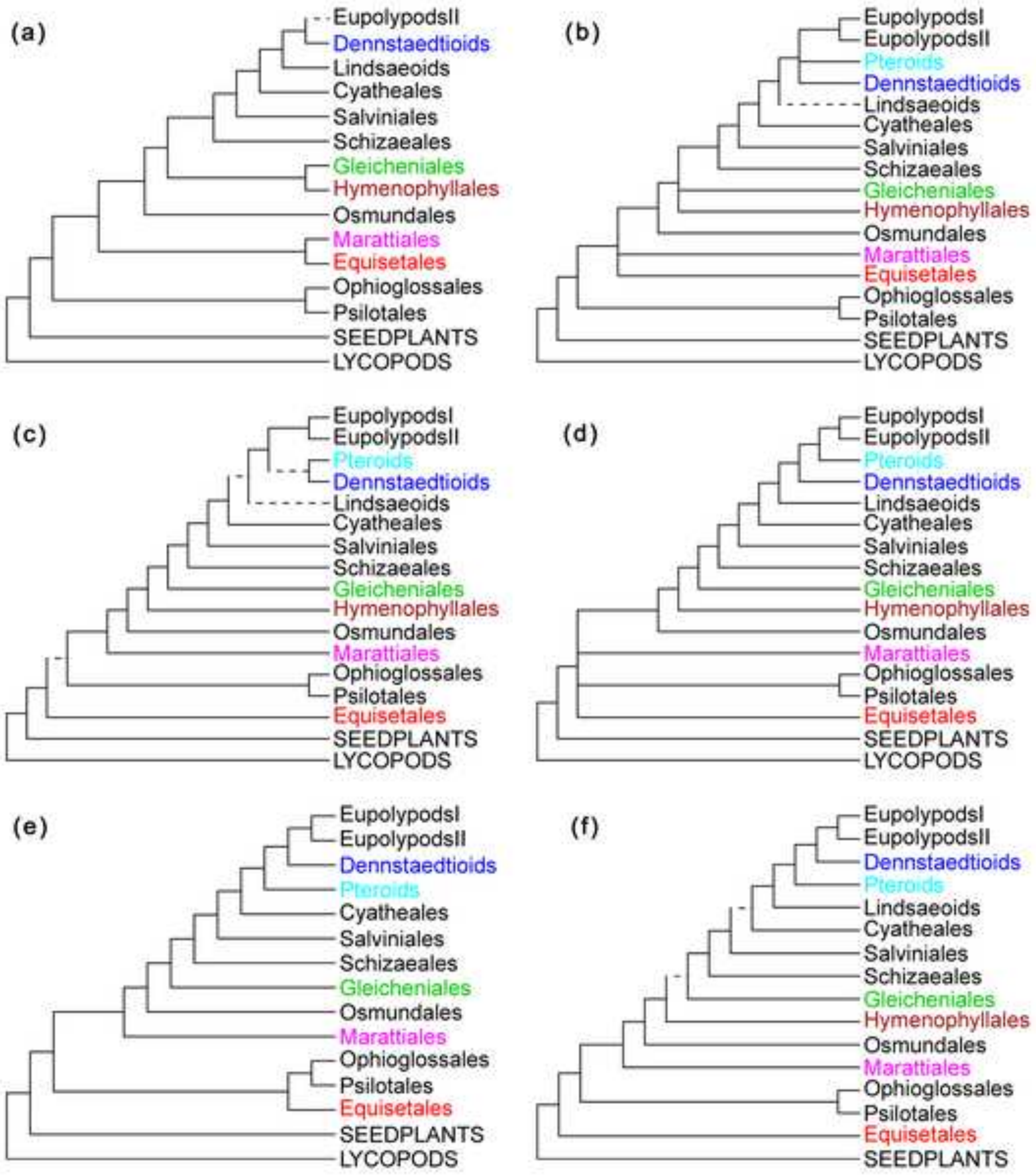
(a)
EupolypodsII
Dennstaedtioids
Lindsaeoids
Cyatheales
Salviniales
Schizaeales
Gleicheniales
Hymenophyllales
Osmundales
Marattiales
Equisetales
Ophioglossales
Psilotales
SEEDPLANTS
LYCOPODS
(b)
EupolypodsI
EupolypodsII
Pteroids
Dennstaedtioids
Lindsaeoids
Cyatheales
Salviniales
Schizaeales
Gleicheniales
Hymenophyllales
Osmundales
Marattiales
Equisetales
Ophioglossales
Psilotales
SEEDPLANTS
LYCOPODS
(c)
EupolypodsI
EupolypodsII
Pteroids
Dennstaedtioids
Lindsaeoids
Cyatheales
Salviniales
Schizaeales
Gleicheniales
Hymenophyllales
Osmundales
Marattiales
Ophioglossales
Psilotales
Equisetales
SEEDPLANTS
LYCOPODS
(d)
EupolypodsI
EupolypodsII
Pteroids
Dennstaedtioids
Lindsaeoids
Cyatheales
Salviniales
Schizaeales
Gleicheniales
Hymenophyllales
Osmundales
Marattiales
Ophioglossales
Psilotales
Equisetales
SEEDPLANTS
LYCOPODS
(e)
EupolypodsI
EupolypodsII
Dennstaedtioids
Pteroids
Cyatheales
Salviniales
Schizaeales
Gleicheniales
Osmundales
Marattiales
Ophioglossales
Psilotales
Equisetales
SEEDPLANTS
LYCOPODS
(f)
EupolypodsI
EupolypodsII
Dennstaedtioids
Pteroids
Lindsaeoids
Cyatheales
Salviniales
Schizaeales
Gleicheniales
Hymenophyllales
Osmundales
Marattiales
Ophioglossales
Psilotales
Equisetales
SEEDPLANTS

 Click here to download Figure Figure 2.bmp

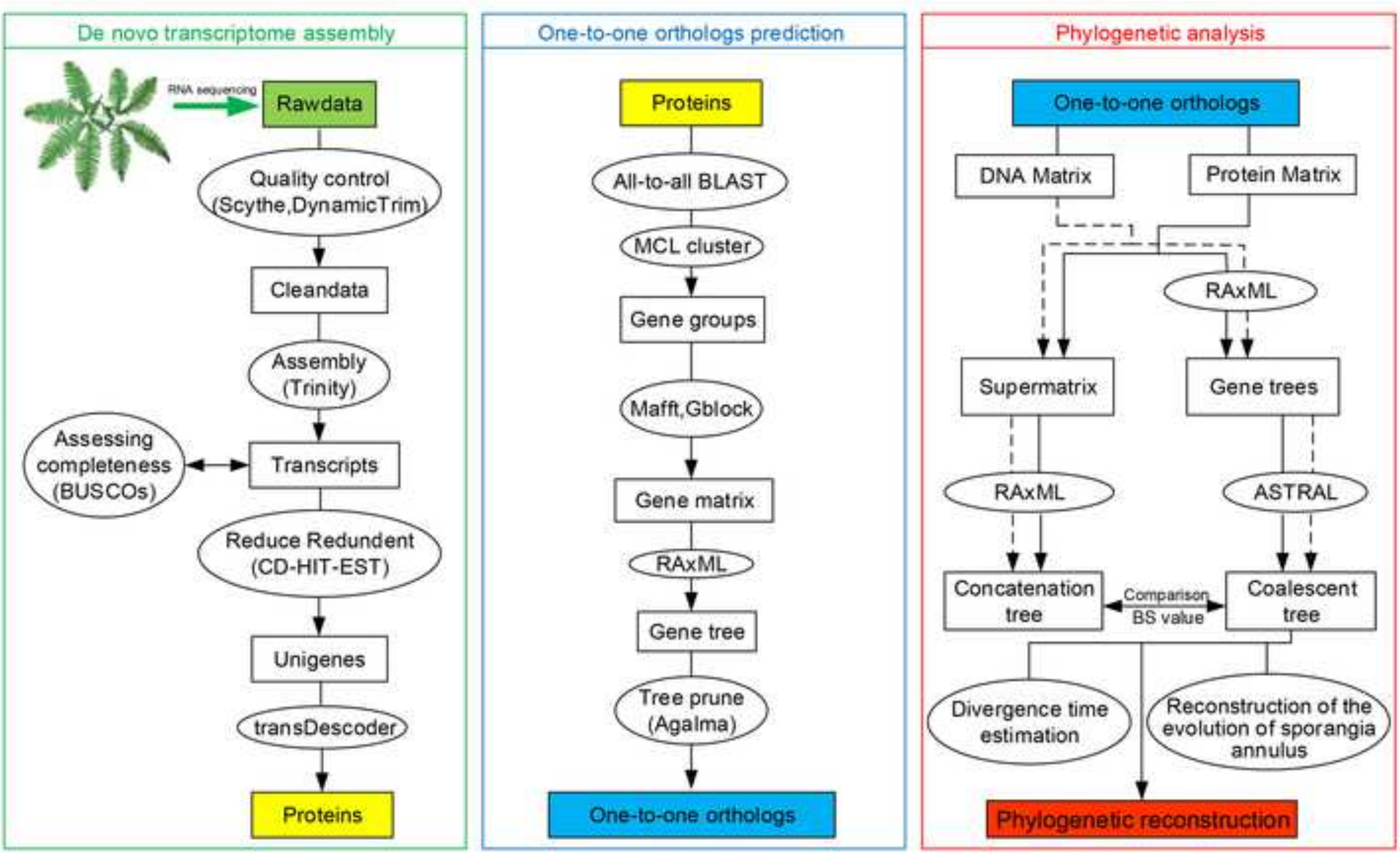

Figure 3

Click here to download Figure Figure 3.bmp

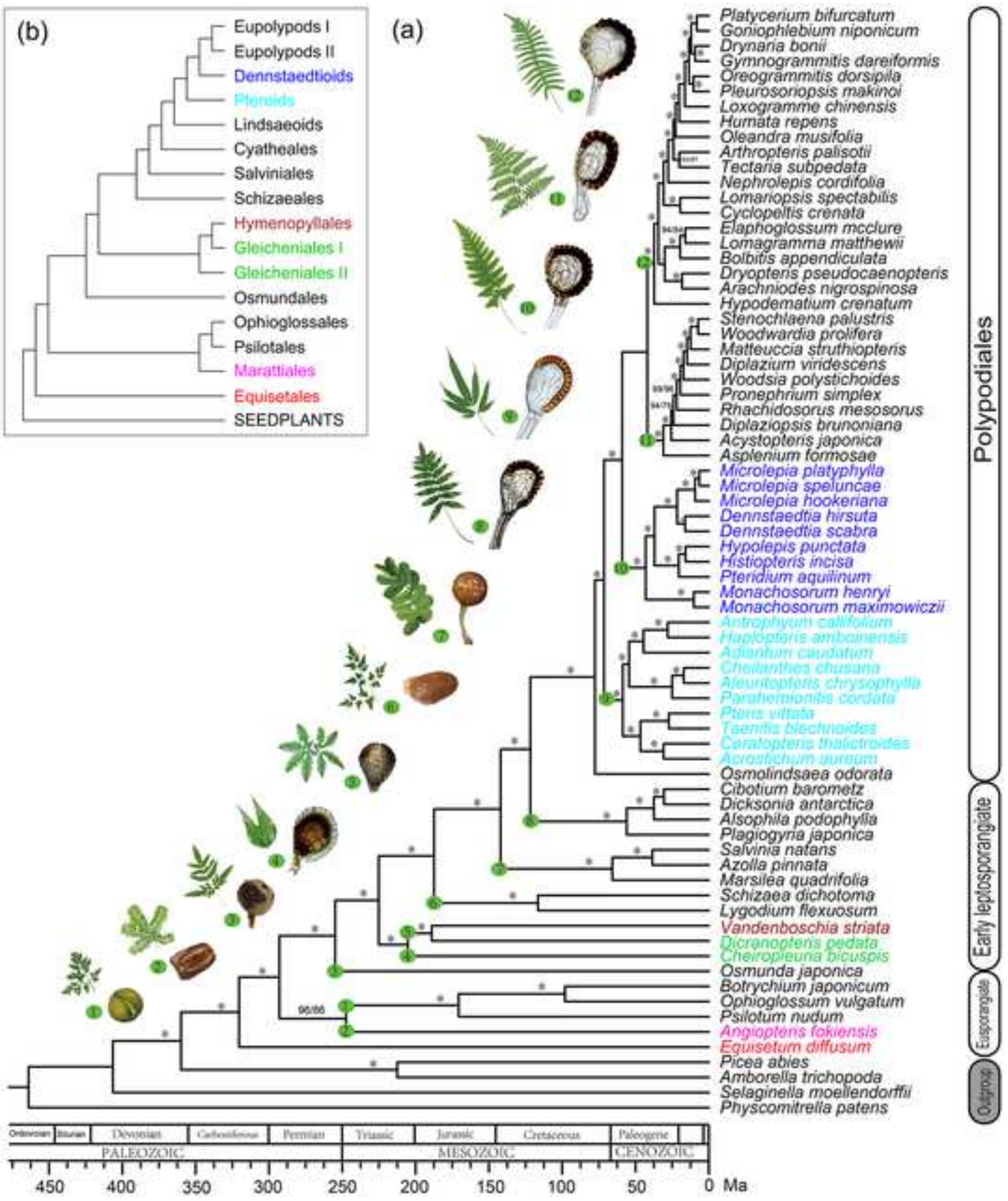

Figure 4

Click here to download Figure Figure 4.bmp

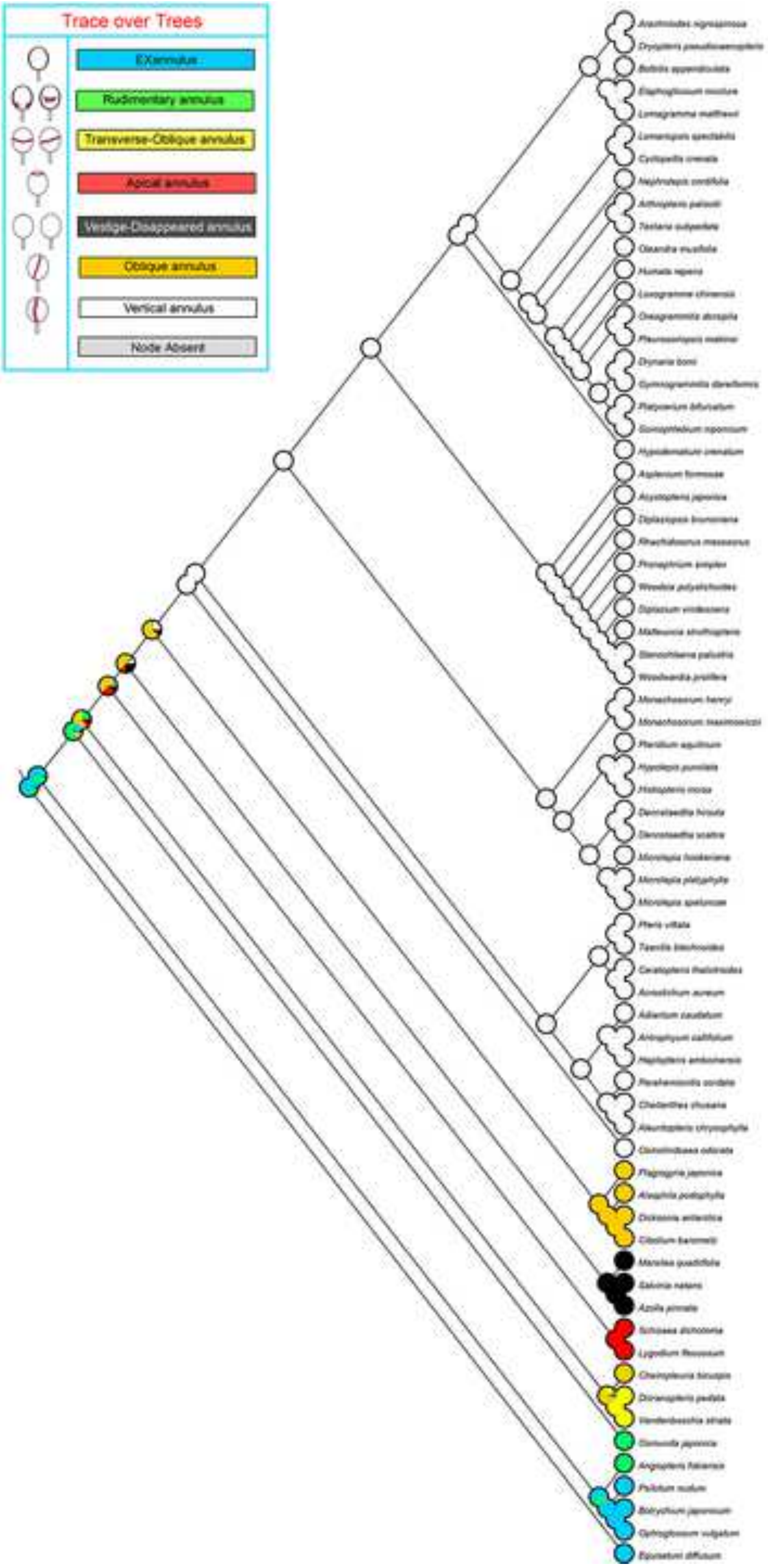

Click here to access/download
Supplementary Material
Supplementary information.docx

Yue-Hong Yan, Professor
Shanghai Chenshan Plant Science Research Center,
Chinese Academy of Sciences & Shanghai Chenshan Botanical Garden
3888 Chenhua Road, Shanghai 201602, China
Tel: +86-21-37792288-903; Fax: +86-21-67657811; email: yhyan@sibs.ac.cn
July 7, 2017

Dear Editor for GigaScience:

We have revised a manuscript entitled "**Large scale phylogenomic analysis resolves a backbone phylogeny in ferns**" (formerly manuscript number: GIGA-D-17-00009) for your consideration to be published in **GigaScience**. The materials in the manuscript have not been published, nor are under consideration for publication elsewhere.

Ferns are the sister group of seed plants. However, the relationships among major fern lineages remain controversial. Here, we carried a large scale phylogenomic analysis using high-quality transcriptome sequencing data which covered 69 fern species from all the 11 orders. By comparing the cladograms yielded from various methods of species tree estimation, we obtained a robust fern phylogeny. Our results are interpreted with sporangia characters, and a new evolutionary route of sporangia in ferns are suggested. This backbone phylogeny in ferns sets a foundation for further studies in biology and evolution in ferns, and therefore in plants, especially when fern genomes are not available.

We have adopted all the suggestions in our revised manuscript. The major revisions include: 1 during species tree estimation, we applied both coalescent-based and concatenation-based methods to both nucleotides and amino acids sequences, and compared the results; 2 we used fossil records to estimate the divergence times; 3 before discussing the evolution of sporangia in ferns, we reconstruct the ancestral state of sporangium annulus; 4 we added a working flow diagram (Figure 2) to show the major processes of data production and analysis; 5 we deposited the datasets, trees, and scripts in open repositories, including GenBank, figshare, and github; 6 we improved our "tree thinking" and writing.

Thank you very much for handling our manuscript. I am looking forward to hearing your decision soon.

Sincerely yours,
Yue-Hong Yan

Response to the review comments:

Reviewer #1: Shen et al presented an impressive dataset on fern transcriptomes, and attempted to build a better backbone phylogeny of ferns. Despite that I believe the transcriptome data will be invaluable for the community, authors' phylogenetic analyses, and the interpretation of the results, are inadequate for publication. My major concerns are:

(1) The authors concatenated all the loci for phylogenetic reconstruction, a practice that has been shown prone to give high supports on wrong relationships. There are numerous simulation and empirical studies on the danger of data concatenation in phylogenomics. Just name a few: http://currents.plos.org/treeoflife/article/concatenation-analyses-in-the-presence-of-incomplete-lineage-sorting/ and http://www.nature.com/nature/journal/v497/n7449/abs/nature12130.html. Concatenation is particularly inappropriate when there are incomplete lineage sorting, and I believe some of the "novel" relationships the authors found may not be true, but due to the pitfalls of their phylogenetic methodology. The better approach would be to use multi-species coalescent method, like ASTRAL (Mirarab et al 2014 Bioinformatics 30: i541-i548).

R: We thank the reviewer for that these suggestions are very helpful. We have applied both coalescent-based and concatenation-based methods in species tree estimation in the revised manuscript (Line 303-312). The coalescent-based species tree was reconstructed by ASTRAL (v4.10.4) (Line 303-304). When nucleotide sequence is used, the cladograms yielded from coalescent-based and concatenation-based methods are highly consistent, except the location of *Angiopteris fokiensis* (Table 2, Line 531).

(2) The authors did not address how they deal with transcript isoforms from the Trinity output. When there are multiple isoforms, which one did you include in the alignment?

R: We thank the reviewer for the suggestion. In our pipeline, we used CD-HIT-EST (v4.6.1) to cluster contigs, followed by discard the duplicated sequences (Line 255-257). The modification has been incorporated in the revised version of the manuscript.

(3) I don't have the access to the alignments to assess the quality. And the alignment and tree files should be deposited in Dryad, TreeBase, or other open repositories.

R: We thank the reviewer for the suggestion. We have deposited the datasets in the open repository “Figshare” , including:

- The 4 alignment sets of single orthologous gene (including 2 matrices both in DNA and Protein sequences)，available at https://figshare.com/s/f835735cb66911ff1ffd

The datasets for concatenation based phylogenetic tree, including:

- The 4 concatenation matrices (including 2 matrices both in DNA and Protein sequences);

- The results of model selection for Protein concatenation tree (We did not adopt partition method here, the model for each gene was the same; For DNA concatenation tree, the default model $\mathrm{GTR}+\Gamma_4+\mathrm{I}$ was used);
- The 4 resulting concatenation tree files (inferred by 2 matrices both in DNA and Protein sequences).

These data are available at https://figshare.com/s/8af236b660f61078e40b.

For coalescent-based species tree, the deposited data including:

- The 4 single orthologous gene matrices sets (including 2 matrices both in DNA and Protein sequences);
- The best gene trees selected from the 100 replicated tree inferred by each gene matrices, which were used to calculate the topology of the consensus coalescent-based species tree;
- The 100 random gene trees of each gene matrices, which were used to calculate the bootstrap of the consensus coalescent-based species tree;
- The 4 coalescent-based species trees in newick format (inferred by 2 matrices both in DNA and Protein sequences).

These data are available at https://figshare.com/s/e5e70c2fd3990e5176d8.

(4) The authors do not have the correct "tree-thinking". The extant Equisetum is not more primitive/basal/earlier than say Polypodium. Their interpretation on annulus evolution also assumed a "ladderized" progression from Equisetales, Ophioglossales, Marattiales, Gleicheniales, Schizaeales, to others. But remember the trees can be freely rotated! If you want to make claims on the evolutionary "route", use fossils and/or character state reconstruction. Please refer to Stacey Smith and David Baum's Tree Thinking book, and also this blog post http://for-the-love-of-trees.blogspot.com/2016/09/the-ancestors-are-not-among-us.html.

R: These suggestions are very helpful. We have referred to the literature which the reviewer suggested, and improved our “tree-thinking”. We have removed words like “basal”, ”primitive” when we describe phylogenetic relationships and have used “sister to” instead. In the revised manuscript, to analyze the evolutionary route of sporangia annulus, we have used fossil records (Line 311-316, Figure 3) to estimate the divergence times, and applied character state reconstruction (Line 317-332, Figure 4).

(5) The authors also made too dramatic a claim that "deep relationships in fern phylogeny remain weakly supported and controversial" (line 60-61). Rothfels et al (2015 AJB 102: 1-19) already showed high supports for the relationships among Equisetales, Psilotales, Ophioglossales, Marattiales, and leptosporangiates.

R: Researches (Pryer et al. 2004, Smith et al. 2006, Rai and Graham 2010, Schneider 2013, Lu et al. 2015, Rothfels et al. 2015) have yielded conflicting cladograms among major lineages in ferns (Figure 1), despite that Rothfels et al (2015 AJB 102: 1-19) showed high supports for some deep relationships. In agree with the reviewer’s opinion partially, we have changed the sentence as “some major relationships in fern phylogeny remain controversial” (Line 57-58).

(6) The writing needs to be tightened up a lot. There are many typos and awkward grammar.

R: We thank the reviewer for the suggestion. We have improved the writing and checked the grammar carefully.

In summary, I think this study by Shen et al lacks the rigor and to some extent the novelty, despite having generated this large amount of data. The authors should consider improving their phylogenetic methods, and rethink about what new insights can be generated from their dataset. Good luck :)

R: These suggestions are very helpful. In the revised manuscript, we have improved the phylogenetic methods greatly, such as to estimate species tree using both coalescent-based and concatenation-based methods; and to reconstruct character state of sporangium annulus; and improved our "tree thinking" also. We have found some new insights, such as that eusporangiate ferns except Equisetales form a monophyletic clade, and that Gleicheniaceae and Hymenophyllaceae form a monophyletic clade which is sister to Dipteridaceae.

Reviewer #2: I review the paper not as an expert in fern evolution but just to assess the appropriateness of the methodology and data reporting. The paper report on a concatenation analysis of transcriptomic data for 69 fern species. The problem addressed is interesting, and the dataset is large and promising. The language is mostly clear, although improvements are needed (examples of problematic sentences are shown in the attached file). The paper certainly has merit.

There are three issues with the current version of the manuscript. The first two issues have to be solved before the paper becomes suitable for publication at GigaScience. The third issue raised can be taken as a suggestion.

1- Reproducibility: A major premise of journals like GigaScience is that data and methods used should be made available. The authors have deposited transcriptomes to GenBank. But this is not nearly enough. They should also make available:

- Their ALIGNED orthologous gene sets
- Their filtered data (after GBlocks) in form of the concatenation matrix used.
- The results of model selection (I assume one model per gene, but this was not clear).
- Resulting trees, in newick or other machine-readable formats. Authors put some newick strings (I don't think for the main tree) in the supplement. This is not the most useful way to publish newick trees. Instead, they should be made available as files. Places like TreeBase and dryad can be used for depositing the trees.

R: We thank the reviewer for the suggestion. We have deposited the datasets in the open repository "Figshare" , including:

- The 4 alignment sets of single orthologous gene (including 2 matrices both in DNA and Protein sequences)，available at https://figshare.com/s/f835735cb66911ff1ffd;

The datasets for concatenation based phylogenetic tree, including:

- The 4 concatenation matrices (including 2 matrices both in DNA and Protein sequences);
- The results of model selection for Protein concatenation tree (We did not adopt partition method here, the model for each gene was the same; For DNA concatenation tree, the default model $\mathrm{GTR} + \Gamma_4 + \mathrm{I}$ was used);
- The 4 resulting concatenation tree files (inferred by 2 matrices in both DNA and Protein sequences).

These data are available at https://figshare.com/s/8af236b660f61078e40b.

For coalescent-based species tree, the deposited data include:

- The 4 single orthologous gene matrices sets (including 2 matrices in both DNA and Protein sequences);
- The best gene tree selected from 100 replicated trees which were inferred from each gene matrices. These best gene trees were used to calculate the topology of the consensus coalescent-based species tree;
- The 100 random gene trees from each gene matrices, which were used to calculate the bootstrap of the consensus coalescent-based species tree;
- The 4 coalescent-based species tree in newick format (inferred by 2 matrices in both DNA and Protein sequences).

These data are available at https://figshare.com/s/e5e70c2fd3990e5176d8.

In addition, the authors describe the methods but do not make any of their scripts available. Making those available would be important for reproducibility. At a minimum, the exact commands used for running various tools (e.g., MCL, Mafft, Trinity, RAxML, etc.) should be provided in the supplement. In absence of details I had to assume certain things. For example, it seemed like the analyses were partitioned, but details were not clear.

R: We thank the reviewer for the suggestion. We have deposit the scripts (including assembly, MCL, alignment, tree prune, matrix construction, RAxML, etc) in github (https://github.com/shenhui0713/Paper-2017-Ferns_69.git) and Figshare (https://figshare.com/s/b28085ee6a7b69f758e9) with a description of each script.

2- It was not clear to me how the dataset was divided into primitive and derived taxa. The criteria should be described clearly. The abstract should not use these two terms assuming their meaning is clear to the reader. In general, calling taxa derived and primitive tends to be controversial (to say the least).

R: We thank the reviewer for the suggestion. In the revised manuscript, we did not divide the sampled species into primitive and derived taxa; instead, we have grouped the species as Eusporangiate, Early leptosporangiate, and Polypodiales according to the

phylogeny. Moreover, since words like primitive and derived taxa are not correct “tree thinking”, we have avoided using them in the revised version.

3- The methods used are OK, but do not include the types of species tree analyses that are the norm in modern phylogenomics. The authors have >1000 genes. They can estimate individual gene trees and then combine them using a summary method (e.g., NJst/ASTRID, ASTRAL, MP-EST, etc). This approach would provide an alternative analysis that can be compared with concatenation. The authors should feel free to argue in favor of one type of analysis over the other, but not doing any species tree analysis makes this into a paper that one would have expected to see 5 years ago but not now. At a minimum, the authors should discuss why they don't think a species tree method is needed or relevant.

R: We thank the reviewer for the suggestion. In this revised manuscript, we have applied both coalescent-based and concatenation-based methods in species tree estimation (Line 303-312). The coalescent-based species tree was reconstructed by ASTRAL (v4.10.4) (Line 303). We also compared the cladograms estimated by coalescent-based and concatenation-based methods (Table 2, Line 531).

Reviewer #3: In "Large scale phylogenomic analysis resolves a backbone phylogeny in ferns" Hui Shen and colleague collect RNA-seq data and perform phylogenetic analyses of a large group of ferns to address existing phylogenetic uncertainties.

As agreed by the editor, I am not qualified to assess the biological significance of the findings and accordingly my review will be limited to the technical aspects and the presentation.

Through the application of RNA-Seq, the authors estimate a comprehensive phylogenetic tree of 69 species of ferns covering the major groups, resolving some outstanding placement issues with sufficient confidence. Moreover, they utilize their newly obtained tree to revise the morphological evolution of sporangia, offering a novel hypothesis (this could be better reflected in the title). Overall, I found the study well design and conducted and a valuable addition. I offer some recommendations for some potential improvements below.

For their main result (the estimation of a robust phylogenetic backbone tree) the authors apply a multi-step process composed of sequencing QC, transcriptome assembly, orthology assignment and tree estimation by maximum likelihood from (translated) amino acid sequences. While these are pretty standard procedures, the description of the exact steps could be improved and their purpose more clearly stated: a flow diagram could greatly improve understanding. For example it is not clear which alignments are of nucleotides and which of proteins (eg. line 128 vs. 134). The code used for the various steps was not initially provided and was subsequently made available by the authors as a set of scripts. Some steps of the pipeline are however missing (removal of sequences of species with more than 10 sequences in a group, line 127; tree estimation within groups of

orthologues, line 130) and it is not obvious that the script 4_Runall_for_multi_alignment.pl is (probably) run also after

#2. Overall, these are documentation problems rather than methodological issues and the approach used in this study is sufficiently robust and appropriate. The strong bootstrap support and the topological consistency validate the chosen approach. As mentioned above, more detailed explanations (possibly along with a flow diagram) would help clarify the matter.

R: We thank the reviewer for the comments and suggestions.

- We have added a flow diagram as **Figure 2** to show the major processes and methods in this study.
- For the missing step of "removal of sequences of species with more than 10 sequences in a group" in the pipeline, we have added a new script named "3_Runall_for_mci_result_analysis.pl" which is run for the masking of the resulting homologous gene families obtained by MCL. Within this script, lines 49-53 are coded for "removal of sequences of species with more than 10 sequences in a group".
- For the missing step of "tree estimation within groups of orthologues", we have added a new script named "5_ Runall_for_raxml.pl", which is run for construction of Raxml tree of each homologous gene family using protein sequence.
- For the "script 4_Runall_for_multi_alignment.pl", we have renumbered this script as "script 7_Runall_for_multi_alignment.pl", it could run together with the newly provided script "3_Runall_for_mci_result_analysis.pl", "4_Runall_for_alignment.pl" and "pal2nal.v14 (Open Source Software)", all these scripts have been deposited in github (https://github.com/shenhui0713/Paper-2017-Ferns_69.git) and Figshare (https://figshare.com/s/b28085ee6a7b69f758e9)

Another aspect that needs some more clarification is the purpose and appropriateness of the approximate unbiased (AU) test.

R: We thank the reviewer for the comments. As we have applied both coalescent-based and concatenation-based methods to both nucleotides and amino acids sequences in species tree estimation, and have compared the four resulting cladograms (Table 2, Line 531); the AU test seems not necessary, and has been removed from the revised manuscript.

Below, I offer some recommendations that could make the paper more accessible to non-experts in the field of fern phylogeny and evolution.

Generally, the authors do a good job at introducing the uncertainties in fern phylogeny that they wish to address, however the usage of common names should be moved to the introduction, rather than being left to the Discussion. Similarly, for the non-expert, it is necessary to specify which species belong to which group (Family/Order), either as part of Table 1 and/or as part of Figure 2.

R: Thanks for the suggestions. Since only a few species have common names and they

were not referred to in the introduction, we have removed the common names from the revised manuscript. In order to help the readers find the Order/group to which a certain species belongs, we have color the species names in Figure 3(a) the same as their correspondence groups in Figure 3(b).

However, much of the focus of the study is devoted to addressing the evolution of sporangia given the newly obtained phylogeny. As a new evolutionary pathway is proposed as an important novel result, I suggest introducing the current view in the Introduction, possibly with a diagram (similar to Figure 3), rather than scattered in the Discussion section. I understand that the current phylogeny is (or historically has been) informed by the morphology of the sporangium, therefore being able to map the morphology on an independently obtained phylogeny is a major advancement.

It's surprising that one species (Cyclopeltis crenata) only had 23% coverage of its BUSCO set, compared with most of the others above 90%. This observation, its causes and its potential consequences are not addressed in the text.

R: We thank the reviewer for the comment. We have perform an analysis to assess the potential affection caused by the relative lower assembly quality of *Cyclopeltis crenata*. A RAxML tree was constructed using concatenation method, with the matrix that excluded all the gene sequence of *Cyclopeltis crenata*. We compared the obtained topology with our main tree results, and found no difference. This result implied that the low coverage of BUSCO in the sample Cyclopeltis crenata does not affect our main phylogeny conclusion. In addition, it seems that the orthologous gene presented in *Cyclopeltis crenata* is not deceased even the lower assembly quality: 2146 in the matrix 1, 2391 in total；1279 in the matrix 2, 1334 in total.

My major issue is with Figure 2, which is the centerpiece of the study. In its current form it is underwhelming and I strongly recommend improving the figure and especially the legend. The phylogram should definitely include the group membership of the various species (possibly currently indicated by the A-D and I-IV markings on the side, but not explained) and the legend should explain the changes in sporangium.

R: We thank the reviewer for the comments. We have revised Figure 3 (formerly Figure 2), such as marking the group names on the side of the species names, and adding the time scale. The change of sporangia and their annulus are explained in Figure 4 and the main text (Line 216-228) in the revised manuscript.

Finally, I would strongly urge the authors to deposit the tree and the underlying matrices to a domain specific repository like TreeBASE (https://treebase.org) and/or OpenTreeOfLife (https://tree.opentreeoflife.org/about/open-tree-of-life).

R: We thank the reviewer for the comments. We have deposited the trees and the underlying datasets in the open repository “Figshare” , including:

- The 4 alignment sets of single orthologous gene (including 2 matrices both in DNA and Protein sequences)， available at https://figshare.com/s/f835735cb66911ff1ffd

The datasets for concatenation based phylogenetic tree, including:

- The 4 concatenation matrices (including 2 matrices both in DNA and Protein sequences);
- The results of model selection for Protein concatenation tree (We did not adopt partition method here, the model for each gene was the same; For DNA concatenation tree, the default model $\mathrm{GTR} + \Gamma_4 + \mathrm{I}$ was used);
- The 4 resulting concatenation tree files (inferred by 2 matrices both in DNA and Protein sequences).

These data are available at https://figshare.com/s/8af236b660f61078e40b.

For coalescent-based species tree, the deposited data including:

- The 4 single orthologous gene matrices sets (including 2 matrices both in DNA and Protein sequences);
- The best gene trees selected from the 100 replicated tree inferred by each gene matrices, which were used to calculate the topology of the consensus coalescent-based species tree;
- The 100 random gene trees of each gene matrices, which were used to calculate the bootstrap of the consensus coalescent-based species tree;
- The 4 coalescent-based species trees in newick format (inferred by 2 matrices both in DNA and Protein sequences).

These data are available at https://figshare.com/s/e5e70c2fd3990e5176d8.

Moreover, we have deposit the scripts (including assembly, MCL, alignment, tree prune, matrix construction, RAxML, etc) in github (https://github.com/shenhui0713/Paper-2017-Ferns_69.git) and Figshare (https://figshare.com/s/b28085ee6a7b69f758e9) with a description of each script.

Other minor points

"basing on" should be replaced with "based on" throughout.

l. 51: "phylogenetic studies in ferns"

l. 52: "our understanding of fern evolution"

The sentence in lines 69-70 should be removed as it's factually incorrect.

l. 61: PPG is only spelled out in l. 200

l. 81: "cornerstone of fern phylogeny"

ll. 83-85: unclear what is meant by "A natural framework"

l. 148: a reference to "hypothesys_tree.doc file" is not clear.

ll. 159-160 ("together with..."): incomplete sentence.

l. 182-183: "which is adapted" seems to refer to the AU test, rather than the five topologies.

l. 235: "In agreement [...] Eupolypods consist"

R: We thank the reviewer for the comments. We have revised these inappropriate words/sentences mentioned above, and checked the grammar all over the revised manuscript carefully.
