# Supplementary material for: Large-scale phylogenomic analysis resolves a backbone phylogeny in ferns
Source: Gigascience. 2017 Nov 24;7(2):gix116. doi: 10.1093/gigascience/gix116 (PMC5795342; doi:10.1093/gigascience/gix116)
Supplement: GIGA-D-17-00169_Revision-2.pdf [file gix116_giga-d-17-00169_revision-2.pdf]

GigaScience

Large scale phylogenomic analysis resolves a backbone phylogeny in ferns

--Manuscript Draft--

**Manuscript Number:**	GIGA-D-17-00169R2
**Full Title:**	Large scale phylogenomic analysis resolves a backbone phylogeny in ferns
**Article Type:**	Research
**Funding Information:**	Shanghai Landscaping & City Appearance Administrative Bureau of China (F112422) — Proffesor Yue-Hong Yan<br>Shanghai Landscaping & City Appearance Administrative Bureau of China (G152420) — Proffesor Yue-Hong Yan<br>Shanghai Landscaping & City Appearance Administrative Bureau of China (G142433) — Proffesor Yue-Hong Yan<br>National Natural Science Foundation of China (31370234) — Proffesor Yue-Hong Yan
**Abstract:**	Background: Ferns, originated about 360 million years ago, are the sister group of seed plants. Despite the remarkable progress in our understanding of fern phylogeny, with conflicting molecular evidences and different morphological interpretations, relationships among major fern lineages remain controversial.<br>Results: With the aim to obtain a robust fern phylogeny, we carried a large scale phylogenomic analysis using high-quality transcriptome sequencing data which covered 69 fern species from 38 families and 11 orders. Both coalescent-based and concatenation-based methods were applied to both nucleotide and amino acid sequences in species tree estimation. The resulting topologies are largely congruent with each other, except the placement of Angiopteris fokiensis, Cheiropleuria bicuspis, Diplaziopsis brunoniana, Matteuccia struthiopteris, Elaphoglossum mcclurei and Tectaria subpedata.<br>Conclusions: Our result confirmed that Equisetales is sister to the rest of ferns, and Dennstaedtiaceae is sister to eupolypods. Moreover, our result strongly supported some relationships different from the current view of fern phylogeny, including that Marattiaceae may be sister to the monophyletic clade of Psilotaceae and Ophioglossaceae; Gleicheniaceae and Hymenophyllaceae form a monophyletic clade sister to Dipteridaceae; and that Aspleniaceae is sister to the rest groups in eupolypods II. These results were interpreted with morphological traits, especially sporangia characters, and a new evolutionary route of sporangial annulus in ferns was suggested. This backbone phylogeny in ferns sets a foundation for further studies in biology and evolution in ferns, and therefore in plants.
**Corresponding Author:**	Yue-Hong Yan, Ph.D<br>Shanghai Chenshan Botanical Garden<br>Shanghai, CHINA
**Corresponding Author Secondary Information:**	
**Corresponding Author's Institution:**	Shanghai Chenshan Botanical Garden
**Corresponding Author's Secondary Institution:**	
**First Author:**	Hui Shen, PhD
**First Author Secondary Information:**	
**Order of Authors:**	Hui Shen, PhD<br>Dongmei Jin, PhD<br>Jiang-Ping Shu<br>Xi-Le Zhou

Powered by Editorial Manager® and ProduXion Manager® from Aries Systems Corporation

	Ming Lei, PhD
	Ran Wei, PhD
	Hui Shang
	Hong-Jin Wei
	Rui Zhang, PhD
	Li Liu
	Yu-feng Gu
	Xian-Chun Zhang, PhD
	Yue-Hong Yan, Ph.D
**Order of Authors Secondary Information:**	
**Response to Reviewers:**	Reviewer #1:<br>The current manuscript is in no doubt much better than the first submission :) I have no major comment, but only a few minor editing suggestions:<br><br>page 1, line 5. Shouldn't the affiliation for Xian-Chun Zhang be "4" rather than "3"?<br>R: The affiliation for Xian-Chun Zhang should be "4", thank you very much! (page 1, line 5)<br><br>page 2, line 23-26. This sentence still needs to be clarified. Perhaps you could just say "The resulting topologies are largely congruent with each other, except the placement of [XXX]."<br>R: This sentence has been revised as “The resulting topologies are largely congruent with each other, except the placement of Angiopteris fokiensis, Cheiropleuria bicuspis, Diplaziopsis brunoniana, Matteuccia struthiopteris, Elaphoglossum mcclurei and Tectaria subpedata” (page 2, line 23-26).<br><br>page 2, line 32. "which is" can be dropped, i.e. "Gleicheniaceae and Hymenophyllaceae form a monophyletic clade sister to Dipteridaceae."<br>R: This sentence has been revised as “Gleicheniaceae and Hymenophyllaceae form a monophyletic clade sister to Dipteridaceae” (page 2, line 31-32).<br><br>page 3, line 57. Ophioglossales is misspelled<br>R: “Ophiglossales” has been changed as “Ophioglossales”, thank you. (page 3, line 57)<br><br>page 3, line 60. I'd suggest to use "provides" instead of "represents"<br>R: “Represents” has been changed as “provides”, thank you. (page 3, line 60)<br><br>page 4, line 72. The sentence was started with "Since"; therefore "so" here needs to be dropped.<br>R: This sentence has been revised as “Since concatenation-based estimations of species tree usually have good accuracy under low level of ILS, while coalescent-based methods are developed to overcome the effect of ILS, but are sensitive to gene tree estimation error [21], both concatenation-based and coalescent-based estimations are applied” (page 4, line 69-73). “So” in line 72 has been dropped. Thank you.<br><br>page 4, line 75. You could also mention here the saturation problem in nucleotide dataset.<br>R: This sentence has been revised as “However, the substitutional saturation and compositional bias in nucleotide sequence, especially in the third codon position, may lead to a deviation from the true phylogeny” (page 4, line 75-77).<br><br>page 6, line 112. Phytozome, not Phytozone<br>R: “Phytozone” has been changed to “Phytozome” (page 6, line 112).<br><br>page 7, line 149. The paper by Schuettpelz and Pryer (PNAS 2009) should be cited here to support "radiation in Cenozoic".<br>R: The paper by Schuettpelz and Pryer (PNAS 2009) has been cited to support "radiation in Cenozoic", this sentence has been changed as “Since eupolypods have

	experienced rapid evolutionary radiation in Cenozoic [7], and nucleotide sequences usually provide more information to reconstruct relationships at shallow phylogenetic scale, we consider the topology yielded from nucleotide sequence maybe more reliable" (page 7, line 148-151).<br><br>page 7, line 153. Add "the", i.e. "are often the controversial nodes"<br>R: "The" has been added here as "are often the controversial nodes"(page 7, line 153).<br><br>page 8, line 160. "evidence" is not countable.<br>R: This sentence has been changed as "based on molecular evidence" (page 8, line 160).<br><br>page 10, line 215. I think "ancestral" is a better word than "primitive". Primitive has a bad connotation.<br>R: "Primitive" has been changed as "ancestral" (page 10, line 215).<br><br>page 11, line 224. Change "ancient" to "ancestral".<br>R: "Ancient" has been changed to "ancestral" (page 11, line 224).<br><br>page 11, line 229. Change "Apical" to "apical".<br>R: "Apical" has been changed to "apical" (page 11, line 229).<br><br>page 11, line 233. Again I'd suggest using "ancestral" instead of "primitive".<br>R: "Primitive" has been changed as "ancestral" (page 11, line 233).<br><br>page 19, line 449. Phytozome, not Phytozone<br>R: "Phytozone" has been changed to "Phytozome" (page 19, line 448).
**Additional Information:**	
**Question**	**Response**
Are you submitting this manuscript to a special series or article collection?	No
**Experimental design and statistics**<br><br>Full details of the experimental design and statistical methods used should be given in the Methods section, as detailed in our Minimum Standards Reporting Checklist. Information essential to interpreting the data presented should be made available in the figure legends.<br><br>Have you included all the information requested in your manuscript?	Yes
**Resources**<br><br>A description of all resources used, including antibodies, cell lines, animals and software tools, with enough information to allow them to be uniquely identified, should be included in the Methods section. Authors are strongly encouraged to cite Research Resource Identifiers (RRIDs) for antibodies, model organisms and tools, where possible.	Yes

Powered by Editorial Manager® and ProduXion Manager® from Aries Systems Corporation

Have you included the information requested as detailed in our Minimum Standards Reporting Checklist?	
**Availability of data and materials**<br><br>All datasets and code on which the conclusions of the paper rely must be either included in your submission or deposited in publicly available repositories (where available and ethically appropriate), referencing such data using a unique identifier in the references and in the "Availability of Data and Materials" section of your manuscript.<br><br>Have you have met the above requirement as detailed in our Minimum Standards Reporting Checklist?	Yes

Powered by Editorial Manager® and ProduXion Manager® from Aries Systems Corporation

Manuscript

Click here to download Manuscript
FernPhylogenomicsManuscript_1711_ED.docx

Large scale phylogenomic analysis resolves a backbone phylogeny in ferns

Hui Shen[1,2#], Dongmei Jin[1,2#], Jiang-Ping Shu[1,2], Xi-Le Zhou[1,2], Ming Lei[3], Ran Wei[4], Hui Shang[1,2], Hong-Jin Wei[1,2], Rui Zhang[1,2], Li Liu[1,2], Yu-Feng Gu[1,2], Xian-Chun Zhang[4], Yue-Hong Yan[1,2*]

[#] Equal contributors

*Corresponding author: yhyan@sibs.ac.cn

[1]Shanghai Chenshan Plant Science Research Center, Chinese Academy of Sciences, Shanghai 201602, China; [2]Shanghai Key Laboratory of Plant Functional Genomics and Resources, Shanghai Chenshan Botanical Garden, Shanghai 201602, China; [3]Majorbio Bioinformatics Research Institute, Shanghai 201320, China. [4]State Key Laboratory of Systematic and Evolutionary Botany, Institute of Botany, Chinese Academy of Sciences, Beijing 100093, China.

Abstract

Background: Ferns, originated about 360 million years ago, are the sister group of seed plants. Despite the remarkable progress in our understanding of fern phylogeny, with conflicting molecular evidence and different morphological interpretations, relationships among major fern lineages remain controversial.

Results: With the aim to obtain a robust fern phylogeny, we carried out a large scale phylogenomic analysis using high-quality transcriptome sequencing data, which covered 69 fern species from 38 families and 11 orders. Both coalescent-based and concatenation-based methods were applied to both nucleotide and amino acid sequences in species tree estimation. The resulting topologies are largely congruent with each other, except the placement of *Angiopteris fokiensis*, *Cheiropleuria bicuspis*, *Diplaziopsis brunoniana*, *Matteuccia struthiopteris*, *Elaphoglossum mcclurei* and *Tectaria subpedata*.

Conclusions: Our result confirmed that Equisetales is sister to the rest of ferns, and Dennstaedtiaceae is sister to eupolypods. Moreover, our result strongly supported some relationships different from the current view of fern phylogeny, including that Marattiaceae may be sister to the monophyletic clade of Psilotaceae and Ophioglossaceae; Gleicheniaceae and Hymenophyllaceae form a monophyletic clade sister to Dipteridaceae; and that Aspleniaceae is sister to the rest groups in eupolypods II. These results were interpreted with morphological traits, especially sporangia characters, and a new evolutionary route of sporangial annulus in ferns was suggested. This backbone phylogeny in ferns sets a foundation for further studies in

biology and evolution in ferns, and therefore in plants.

Key Words: phylogenomic, monilophytes, evolution, sporangium, transcriptome

Background

Phylogeny, which reflects natural history, is fundamental to understanding evolution and biodiversity. Ferns (monilophytes), originated about 360 million years (MY) ago, are the sister group of seed plants [1, 2]. With estimated 10,578 extant living species globally [3], they are the second most diverse group of vascular plants. Phylogenetic studies for ferns, especially based on molecular evidence, have been widely carried out in recent decades. These studies have revolutionized our understanding of the evolutionary history of ferns. Milestones included setting ferns as the sister group of seed plants [1, 2], placing Psilotaceae and Equisetaceae within ferns [2, 4, 5], and revealing a major polypods radiation following the rise of angiosperms [6, 7]. Resolutions at shallow phylogenetic depth among families or genera have also been improved remarkably [8-14].

However, previous research on fern phylogeny has mostly relied on plastid genes [10, 12, 13], some combined with a few nuclear genes [4, 5, 14] or morphological traits [5, 11]. Due to incomplete lineage sorting (ILS), genes from different resources often show conflicting evolutionary patterns, especially when based on a limited number of samples, some deep relationships in fern phylogeny remain controversial (Figure 1). In the latest PPG I system [3], which has derived from many recent phylogenetic studies, some important nodes remain uncertain, such as (i) what are the relationships

among Marattiales, Ophioglossales and Psilotales? (ii) are Hymenophyllales and Gleicheniales sister groups? and (iii) what are the relationships among families in eupolypods II?

Transcriptome sequencing (RNA-Seq) provides massive transcript information from the genome. Phylogenetic reconstructions based on RNA-Seq are more efficient and cost-effective than traditional PCR-based or EST-based methods when lacking whole-genome data [15]. Successful cases in recent years include mollusks [16], insects [17], the grape family [18], angiosperms [19], and land plants including six ferns [20]. Here, with the aim to reconstruct the framework of fern phylogeny, we sampled abundant fern species representing all important linages and applied latest phylogenomic analyses based on RNA-Seq.

To reconstruct a robust and well-resolved phylogeny in ferns, applying multiple methods of phylogenomic analysis is extremely important. Since concatenation-based estimations of species trees usually have good accuracy under low level of ILS, while coalescent-based methods are developed to overcome the effect of ILS, but are sensitive to gene tree estimation error [21], both concatenation-based and coalescent-based estimations are applied. Nucleotide sequence, with higher variability than amino acid sequence, usually brings more useful information in phylogeny reconstruction, especially for closely related taxa. However, the substitutional saturation and compositional bias in nucleotide sequence, especially in the third codon position, may lead to a deviation from the true phylogeny. Here, both nucleotide

and amino acid sequences are used in phylogeny reconstruction.

Morphologically, the fern sporangium is an organ for enclosing and dispersing spores, most of which functions like a unique catapult with the annulus [22]. During the last centuries, Bower's hypothesis on the evolution of sporangia with a focus on annulus [23] had been one of the most important cornerstones to fern phylogeny based on morphology [24, 25]. However, this hypothesis has been challenged by somewhat conflicting frameworks of fern phylogeny [4, 10, 12, 14, 26]. A robust framework in fern phylogeny which reflects the evolutionary history will improve our understanding for the evolution of fern sporangia as well as other characters.

Data description

Taxa sampling and RNA-Seq

We chose 69 fern species from 38 families according to PPG I system (totally 48 fern families), covering all the 11 orders (Equisetales, Psilotales, Ophioglossales, Marattiales, Osmundales, Hymenophyllales, Gleicheniales, Schizaeales, Salviniales, Cyatheales, and Polypodiales). Information about the location and time for sampling is given in Table S1. All the sampled species were collected under the permissions of the natural reserves and Shanghai Chenshan Botanical Garden in China.

Sporophyll or/and trophophyll were collected and frozen in liquid nitrogen immediately, and preserved in Ultra-low temperature refrigerator at -80°C before RNA extraction. Total RNA was extracted using TRIzol (Life Technologies Corp.) according to the manufacturer's protocols. The RNA concentration was determined using a NanoDrop spectrophotometer, and RNA quality was assessed with an Agilent

Bioanalyzer. Paired-end reads were generated by Majorbio Company (Shanghai, China) using the HiSeq 2500 system. Raw reads were deposited in NCBI [27].

Transcriptomes assembly and orthology assignment

Transcriptomes data were generated from 69 fern species (Table 1). After filtering, about 2,726.9 million pair-end DNA sequence reads (about 313 Gbp) were retained. We assembled these reads *de novo* and obtained a total of 5,449,842 contigs [28].

In order to obtain a reliable phylogenetic relationship, we selected four species as the outgroup, representing the main lineages of land plants: *Amborella trichopoda* (representing angiosperms), *Picea abies* (representing gymnosperms), *Selaginella moellendorffii* (representing lycophytes) and *Physcomitrella patens* (representing bryophytes). The translated ORF (protein) sequences of these four species were downloaded from Phytozome [29] and used in the following analysis.

To ensure the consistency of phylogenomic analysis, we used a phylogenetic-based ortholog selection method, and obtained two subsets of one-to-one orthologous genes that differed in gene number and species occupancy rate, named “Matrix 1” and “Matrix 2” [30]. Matrix 1 consists of 2391 genes that are present in at least 52 taxa (that is 75% of the 69 taxa in total), resulted in 2,024,565 nucleotide and 674,855 amino acid positions, the gene and character occupancy were 88% and 85% respectively. Matrix 2 consists of 1334 genes that are present in at least 62 taxa (that is 90% of the 69 taxa in total), resulted in 1,171,332 nucleotide and 390,444 amino acid positions, the gene and character occupancy reached 94% and 90% respectively. For each orthologues gene set, coalescent-based and concatenation-based methods were applied separately to both nucleotide and amino acid sequences. A working flow diagram showing the major processes in this study is presented in Figure 2.

Results

Species tree estimated in 69 ferns

For each combination of reconstruction methods (coalescent-based or concatenation-based) and sequence types (nucleotide or amino acid), Matrix 1 and Matrix 2 [31, 32] always yielded the same topology. In general, the four topologies (Figure 3, Figure S1, S2, S3) from a combination of methods and sequence types are consistent except six positions (Table 2). Among the topologies, the one estimated by applying coalescent-based method to nucleotide sequence (Figure 3) and the one applying concatenation-based method (Figure S2) are most congruent.

Reconstruction of the evolutionary history of sporangial annulus

Our reconstruction of the evolution of sporangial annulus (Figure 4) showed that ex-annulus sporangia are inferred to be the ancestral state (proportional likelihood [PL]: 1), and the rest of annulus states are likely derived from ex-annulus sporangia. Vertical annulus is suggested as synapomorphy for all polypod ferns (PL > 0.99). Both oblique annulus and rudimentary annulus have experienced parallel evolution.

Discussion

Comparison of topologies estimated by various methods

By comparing topologies estimated by coalescent-based and concatenation-based method using both nucleotide and amino acid sequences (Table 2), we found that the topologies yielded from coalescent-based and concatenation-based methods using nucleotide sequence are mostly consistent, except the position of *Angiopteris fokiensis.* Topologies yielded from coalescent-based method using nucleotide sequence and amino acid sequence showed three positions of inconsistency, all of which belong to eupolypods. Since eupolypods have experienced rapid evolutionary

radiation in Cenozoic [7], and nucleotide sequences usually provide more information to reconstruct relationships at shallow phylogenetic scale, we consider the topology yielded from nucleotide sequence maybe more reliable. However, the inconsistent positions among topologies often show relatively lower supporting values, and are often the controversial nodes from past studies based on different genes, we suggest such inconsistency might be caused partially by LIS and reticulate evolution.

Relationships of eusporangiate ferns

Which clade is sister to the remaining taxa in ferns is a long-debated question (Figure 1). Our results strongly supported that Equisetales (horsetails) are the sister group to all other monilophytes. This topology confirmed the results reported by Rai & Graham [12], and Kuo *et al.* [33] based on plastid genes, and has been accepted by the PPG I [3] in 2016. Distinct from most fern phylogeny based on molecular evidence (Figure 1), our results based on coalescent method revealed that Psilotales (whisk ferns), Ophioglossales (moonworts), and Marattiales (king ferns) form a monophyletic clade as ((Psilotales, Ophioglossales), Marattiales), which is sister to leptosporangiate ferns. The monophyletic origin of Psilotales, Ophioglossales, and Marattiales, which belong to eusporangiate ferns, is supported by the structure of sporangia. Being different from the leptosporangiate type, sporangia of eusporangiate ferns have no sporangiophore, they are thick in wall and large in volume, produce a large amounts of spores, and have no sporangial annulus or only have a few enlarged parenchyma cells. The incongruence between the results based on coalescent and concatenation methods may be caused by strong ILS effect, which is a main pitfall when using the concatenation method [21].

Relationship of early leptosporangiates

Within early leptosporangiates, our results revealed a new monophyletic clade that Gleicheniaceae (forking ferns) is sister to Hymenophyllaceae (filmy ferns), which is different from the mainstream [3, 10, 12-14, 34]. Similar but still different from the topology (((Dipteridaceae, Matoniaceae), Gleicheniaceae), Hymenophyllaceae) reported by Pryer *et al.* in 2004 [5], in our results, *Cheiropleuria*, which belongs to Dipteridaceae and formerly placed in Gleicheniales [2, 5, 12, 26, 35, 36], is sister to the monophyletic clade of (Gleicheniaceae, Hymenophyllaceae).

This new relationship is supported by sporangia character. Early leptosporangiates [36] are characterized with diverse sporangia and annulus. However, both Gleicheniaceae and Hymenophyllaceae have spherical sporangia with transverse-oblique annulus, as well as a short sporangial stalk connecting to a prominent receptacle [37]. On the other hand, flattened sporangia with slightly oblique annulus are found in *Cheiropleuria.* Moreover, long sporangial stalk and inapparent receptacle are common in *Cheiropleuria*, *Dipteris* and *Matonia*. We suggest Dipteridaceae, probably together with its sister lineage Matoniaceae [5, 12], may be sister to the clade of (Gleicheniaceae, Hymenophyllaceae). According to our results, Gleicheniales, which is comprised of Dipteridaceae, Matoniaceae, and Gleicheniaceae [26], is no longer a monophyletic lineage, but a paraphyletic one.

Relationships within polypod ferns

Polypods include more than 80% of living ferns, and their phylogeny remains

somewhat controversial and elusive [26, 35, 36]. Our results strongly supported that Dennstaedtiaceae instead of Pteridaceae is sister to eupolypods. This pattern confirmed the topology suggested recently by Rothfels *et. al* basing on 25 low-copy nuclear genes [14] and Lu *et. al* based on plastid genes [13], as well as PPG I system [3]. According to our results, relationships of Pteridaceae [34, 36, 38] and Dennstaedtiaceae [36] are also well resolved. Notably, *Monachosorum* is sister to the rest members in Dennstaedtiaceae, rather than being sister to the lineage of Pteridium, Hypolepis and Histiopteris [36].

Our results showed that eupolypods are divided into two major lineages, eupolypods I and eupolypods II in agreement with the consensus opinion [3]. Within eupolypods II, our results supported that Aspleniaceae is the sister group to the rest members, which is different from the current viewpoints [26, 36, 39]. Within eupolypods I, our result strongly supported that Lomariopsidaceae and Nephrolepidaceae form a paraphyletic group, rather than a monophyletic clade based on plastid genes [10, 26, 36].

Our new topology confirmed the morphology-based hypothesis that Dennstaedtiaceae with two indusial, rather than Pteridaceae with one false indusium, is more closely related to eupolypod ferns [40]. In Pteridaceae, the unstable structure of spherical sporangia, including variable annulus and short sporangial stalk, indicates these characters of sporangia are relatively original and are close to those with oblique annulus in early leptosporangiates [23]. We also noticed that the characters of

spherical sporangia with slightly oblique annulus in *Monachosorum* should be more ancestral than the flattened sporangia with typical vertical annulus in other genera of Dennstaedtiaceae. For distinguishing eupolypods I and eupolypods II, the number and shape of the vascular bundles at the base of petiole have been demonstrated to be a powerful diagnostic character [36, 39].

The evolution of sporangial annulus in ferns

By observing the character of sporangial annulus of abundant samples in each fern group, and combining these characters with our well-resolved backbone phylogeny (Figure 3), we reconstructed the evolutionary history of sporangial annulus in ferns (Figure 4). According to the results, we infer that ex-annulus sporangia, as in Equisetaceae, Psilotaceae, and Ophioglossaceae, is the ancestral state in ferns; rudimentary multiseriate annulus, which is inverse U-shaped in Marattiaceae, and U-shaped in Osmundaceae; equatorial transverse-oblique uniseriate annulus, as in Gleicheniaceae and Hymenophyllaceae; oblique annulus as in Cyatheales (tree ferns), and vertical annulus as synapomorphy in polypods, have been derived from the ex-annulus state. Both apical annulus as in Lygodium and Schizaea, and vestige or disappeared annulus as in Salviniales (aquatic ferns) are likely to be specialized in parallel from oblique annulus. Inconsistent with Bower's hypothesis [23], our results showed that sporangia with apical annulus as in Schizaeales are no longer the ancestral type in ferns but a specialized one. Correspondingly, the oldest fossils of Schizaeaceae is now believed to appear in Jurassic (201-145 MY BP) rather than formerly thought Carboniferous (359-252 MY BP) [41].

Conclusion

Our results confirmed that Equisetales is sister to all the other monilophytes, and Dennstaedtiaceae is sister to eupolypods which have been reported previously. Moreover, our results revealed some new relationships, such as eusporangiate ferns except Equisetales may form a monophyletic clade as ((Psilotaceae, Ophioglossaceae), Marattiaceae); while Gleicheniaceae and Hymenophyllaceae form a monophyletic clade which is sister to Dipteridaceae; and Aspleniaceae is sister to the rest groups in eupolypods II. Most of these results are supported by sporangia characters, and a new evolutionary route of sporangial annulus in ferns is suggested.

Potential implications

Here, we present a robust fern phylogeny yielded from a largescale phylogenomic analysis based on a high-quality RNA-seq dataset covering 69 fern specie. This backbone phylogeny in ferns sets a foundation for further studies in biology and evolution in ferns and therefore in plants, especially when fern genomes are not available.

Methods

***De novo* transcriptome assembly**

For each paired-end library, we first removed the Illumina adapter of raw reads using Scythe (Scythe, RRID:SCR_011844) [42] and trimmed the poor quality bases using DynamicTrim Perl script of the SolexQA package with default parameters [43]. Next, *de novo* transcriptome assembly of each species was conducted using the Trinity package, version: trinityrnaseq_r20140413 (Trinity, RRID:SCR_013048) with default parameters [44]. To discard the duplicated sequences, the obtained contigs were

clustered using CD-HIT-EST v4.6.1 (CD-HIT, RRID:SCR_007105) to generate non-redundant contigs. All contigs longer than 200 bp in length were used for downstream analysis. We used TransDescoder, a program in the Trinity package, to identify the candidate coding sequences (CDSs) from the contigs with default criteria. Finally, the translated protein sequences of CDSs were searched by BLASTP against the non-redundant protein database in NCBI with an e-value threshold of 1e-5. These BLASTP hit sequences were used for further analysis.

Orthology assignment, alignment, and alignment masking

For orthology assignment for the 69 sample assemblies together with the four outgroup species a phylogenetic based clustering method described previously [16] was used. In short, all-vs-all BLAST search of amino acid sequence was performed across different species, the BLAST results were clustered using MCL [45] software with the parameters '-I 2–tf ′gq(20)″. Optimization of the inflation parameter (I) was conducted as described previously [46], the default value 2.0 was selected ultimately. As the *de novo* assembly by Trinity produces many sequences with high similarity, which contain both paralogs and isoforms [47], when a clustered gene family contains too many sequences (eg. more than 10), the risk of contamination of isoforms rises, along with the computational infeasibility. Hence, when a species had more than 10 sequences in a gene family, we removed all sequences in this gene family of this species. Then, groups with at least 35 (50%) fern species were aligned using einsi command, implemented in MAFFT (MAFFT, RRID:SCR_011811) [48], and trimmed by Gblocks with default parameters [49]. Next, for each group, homologous gene tree was built with RAxML software, version: 8.0.20 (RAxML, RRID:SCR_006086), by implementing the maximum likelihood method (ML) [50]. To infer orthologous genes, we used treeprune in the Agalma package [51] to mask the monophyletic sequences. We pruned the paralogous subtrees from the homologous gene trees until only one monophyletic subtree retained. Next, the resulted orthologous gene trees were further

filtered by the criteria that each species should be represented by only one sequence, this resulted subset genes were referred to “one-to-one orthologs”, which were largely free of gene duplication. Then, we extracted both the CDSs (nucleotide sequence) and translated amino acid sequence from each orthologous gene group, followed by aligning with MAFFT and trimming with Gblocks. The alignment with coding and corresponding translated sequences longer than 150 bp (or 50 amino acids) in length were kept for the further analysis.

BUSCO analysis

The Basic Universal Single Copy Orthologs (BUSCO, RRID:SCR_015008), which employs a core set of orthologs conservative in eukaryotic species to determine the gene coverage of each assembly [52], was employed to assess the completeness of the transcriptome assembly we obtained (Table S2) [53]. A total of 303 BUSCOs were employed to blast against by translated amino acid of the assemblies using BLASTP. Then the number of complete and partially matched gene from each assembly was counted respectively. Out of the 69 samples in total, the gene coverage of 65 samples (94.2%) exceeded 82%, with at least 251 complete genes identified. Unexpectedly, among our total assemblies, 1 sample (*Aleuritopteris chrysophylla*, named RS_72) presented extremely low gene coverage degree, in which only 72 (23.8%) complete housekeeping genes were found (Supplementary Table 2). However, when the sample was deleted from the matrix used to construct the backbone of the phylogenetic tree, the topology remained unchanged, indicating that the lower completeness in this sample did not affect our results (data not shown).

Phylogenetic analysis

The coalescent-based species trees were reconstructed by ASTRAL v4.10.4 [54], carried out 100 replicates of multi-locus bootstrapping [55]. Each gene tree was constructed with the PROTCATJTTF model by RAxML v8.2.4 (RAxML,

RRID:SCR_006086) [50], performed 100 random replicates to calculate bootstrap value. For the concatenation analysis, we preformed the maximum likelihood analyses (ML) for each matrix using RAxML software (version: 8.0.20). The branch support was evaluated using 100 bootstrap replicates. We used the “GTR + Γ4 + I” model for DNA matrices, and the JTTF model for the corresponding protein matrices, selected by “ProtienModelselection.pl” [56]. To estimate the divergence times, we used the concatenated alignment of orthologs, calibrated with ages of two fossils (*Archaeocalamites Senftenbergia*: 354 MY, *Grammatopteris*: 280 MY [6, 57]) as the minimum ages of monilophytes and leptosporangiate ferns, respectively, and a maximum age constraint of 500 MY for land plants, in a Bayesian relaxed clock method using MCMCTREE [58] on the coalescent-based species tree.

Reconstruction of the evolution of sporangial annulus

Characters of sporangial annulus of the sampled species were observed using a polarized light microscope (Axio Scope.A1, ZEISS) after the fresh and mature sporangia were treated with sodium hypochlorite (NaClO) solution. The evolution of sporangial annulus was reconstructed with likelihood method implemented in Mesquite v2.7.5 [59]. All character states (i.e., vertical annulus, oblique annulus, rudimentary annulus, ex-annulus, apical annulus, transverse annulus, and vestigial annulus) were treated as unordered and equally weighted. To reconstruct character evolution, a maximum likelihood approach using Markov k-state 1 parameter model [60] was applied. To account for phylogenetic uncertainty, the “Trace-characters-over-trees” command was used to calculate the ancestral states at each node including probabilities in the context of likelihood reconstructions. To carry out these analyses, characters were plotted onto 100 trees that were sampled in the ML analyses of the

combined dataset using RAxML v7. The results were finally summarized as percentage of changes of character states on a given branch among all 100 trees utilizing the option of “Average-frequencies-across-trees”.

Declarations

List of abbreviations

BUSCOs, the basic universal single copy orthologs;

ILS, incomplete lineage sorting;

MY, million years;

PPG, the pteridophyte phylogeny group;

RNA-Seq, transcriptome sequencing.

Additional files

Additional file1: Tables S1 to S2 and Figures S1 to S3.

Availability of data and materials

Raw reads of RNA-Seq for 69 fern species were deposited in GenBank under Bioproject accession number PRJNA281136. Transcriptome datasets, alignments, phylogenetic trees, BUSCO results and other supporting data are available via the *GigaScience* repository GigaDB [61].

Consent for publication

Not applicable.

Competing interests

The authors declare that they have no competing interests.

Funding

This work was funded by Shanghai Landscaping & City Appearance Administrative Bureau of China, Scientific Research Grants (G142433, G152420 and F112422) and the National Natural Science Foundation of China (31370234).

Authors' contributions

YHY, HShen and DMJ conceived and designed the study. ML, JPS, DMJ, RW and LL implemented the data analyses. YHY, HShen, HJW, XLZ, HShang and YFG collected the specimens. HShen, RZ and YFG prepared the specimens for sequencing. XLZ provides the anatomical data. DMJ, HShen, YHY, JPS, ML, RW, HShang, XLZ and XCZ interpreted the results and wrote the manuscript.

Acknowledgements

We thank Prof. Yong-Hong Hu, Prof. Jin-Shuang Ma, Prof. Zhao-Qing Chu and Dr. Jun Yang from Shanghai Chenshan Botanical Garden of China, as well as Prof. Fu-Wu Xing from South China Botanical Garden of CAS for helpful comments and suggestions. We thank Prof. Paul G. Wolf from Utah State University, Prof. Yin-Long Qiu from University of Michigan and Dr. Jin-Long Zhang from Kadoorie Farm and Botanic Garden for providing important suggestions regarding the research method. We appreciate helpful comments and suggestions from three reviewers of previous versions of this manuscript.

Ethics approval and consent to participate

Not applicable.

References

1. Duff RJ, Nickrent DL. Phylogenetic relationships of land plants using mitochondrial small-subunit rDNA sequences. Am J Bot, 1999;86:372-86.

2. Pryer KM, Schneider H, Smith AR, Cranfill R, Wolf PG, Hunt JS, et al. Horsetails and ferns are a monophyletic group and the closest living relatives to seed plants. Nature. 2001;409:618-22.

3. The Pteridophyte Phylogeny Group. A community-derived classification for extant lycophytes and ferns. J Syst Evol. 2016. doi:10.1111/jse.12229

4. Qiu Y-L, Li L, Wang B, Chen Z, Knoop V, Groth-Malonek M, et al. The deepest divergences in land plants inferred from phylogenomic evidence. Proc Natl Acad Sci USA. 2006;103:15511-6.

5. Pryer KM, Schuettpelz E, Wolf PG, Schneider H, Smith AR, Cranfill R. Phylogeny and evolution of ferns (monilophytes) with a focus on the early leptosporangiate divergences. Am J Bot. 2004;91:1582-98.

6. Schneider H, Schuettpelz E, Pryer KM, Cranfill R, Magallon S, Lupia R. Ferns diversified in the shadow of angiosperms. Nature. 2004;428:553-7.

7. Schuettpelz E, Pryer KM. Evidence for a Cenozoic radiation of ferns in an angiosperm-dominated canopy. Proc Natl Acad Sci USA. 2009;106:11200-5.

8. Zhang LB, Zhang L, Dong SY, Sessa EB, Gao XF, Ebihara A. Molecular circumscription and major evolutionary lineages of the fern genus Dryopteris (Dryopteridaceae). BMC Evol Biol. 2012;12:180-94

9. Liu H-M, Zhang X-C, Wang W, Qiu Y-L, Chen Z-D. Molecular phylogeny of the fern family dryopteridaceae inferred from chloroplast rbcL and atpB genes. Int J Plant Sci. 2007;168:1311-23.

10. Liu H-M. Embracing the pteridophyte classification of Ren-Chang Ching using a generic phylogeny of Chinese ferns and lycophytes. J Syst Evol. 2016;54:307-35.

11. Schneider H, Smith AR, Pryer KM. Is morphology really at odds with molecules in estimating fern phylogeny? Syst Bot. 2009;34:455-75.

12. Rai HS, Graham SW. Utility of a large, multigene plastid data set in inferring higher-order relationships in ferns and relatives (monilophytes). Am J Bot. 2010;97:1444-56.

13. Lu J-M, Zhang N, Du X-Y, Wen J, Li D-Z. Chloroplast phylogenomics resolves key relationships in ferns. J Syst Evol. 2015;53:448-57.

14. Rothfels CJ, Li F-W, Sigel EM, Huiet L, Larsson A, Burge DO, et al. The evolutionary history of ferns inferred from 25 low-copy nuclear genes. Am J Bot. 2015;102:1089-107.

15. Hittinger CT, Johnston M, Tossberg JT, Rokas A. Leveraging skewed transcript abundance by RNA-Seq to increase the genomic depth of the tree of life. Proc Natl Acad Sci USA. 2010;107:1476-81.

16. Smith S, Wilson N, Goetz F, Feehery C, Andrade S, Rouse G, et al. Resolving the evolutionary relationships of molluscs with phylogenomic tools. Nature. 2011;480:364-7.

17. Misof B, Liu S, Meusemann K, Peters RS, Donath A, Mayer C, et al. Phylogenomics resolves the timing and pattern of insect evolution. Science. 2014;346:763-7.

18. Wen J, Xiong Z, Nie Z-L, Mao L, Zhu Y, Kan X-Z, et al. Transcriptome sequences resolve deep relationships of the grape family. Plos One. 2013;8:e74394.

19. Zeng L, Zhang Q, Sun R, Kong H, Zhang N, Ma H. Resolution of deep angiosperm phylogeny using conserved nuclear genes and estimates of early divergence times. Nature Communications. 2014. doi:10.1038/ncomms5956

20. Wickett NJ, Mirarab S, Nam N, Warnow T, Carpenter E, Matasci N, et al. Phylotranscriptomic analysis of the origin and early diversification of land plants. Proc Natl Acad Sci USA. 2014;111:E4859-68.

21. Mirarab S, Bayzid MS, Boussau B, Warnow T. Statistical binning enables an accurate coalescent-based estimation of the avian tree. Science, 2014.doi:10.1126/science.1250463

22. Noblin X, Rojas N, Westbrook J, Llorens C, Argentina M, Dumais J. The fern sporangium: a unique catapult. Science. 2012;335:1322.

23. Bower FO. The Ferns (Filicales) treated comparatively with a view to their natural classification. Vol 1-3. London: Cambridge University Press. 1923-1928.

24. Pichi-Sermolli REG. Historical review of the higher classification of the Filicopsida. In: Jermy AC, Crabb JA, Thomas BA, editors. Phylogeny and classification of the ferns. London: Bot J Linn Soc. 1973. p.11-40.

25. Smith AR. Non-molecular phylogenetic hypotheses for ferns. Am Fern J. 1995;85(4):104-22.

26. Smith AR, Pryer KM, Schuettpelz E, Korall P, Schneider H, Wolf PG. A classification for extant ferns. Taxon. 2006;55:705-31.

27. Raw reads. https://www.ncbi.nlm.nih.gov/bioproject/?term=PRJNA281136. Accessed 5 July 2017.

28. Transcriptome datasets. https://figshare.com/s/0f773861b6813f97ff63. Acessed 5 July 2017.

29. Phytozome. http://phytozome.jgi.doe.gov/. Accessed 5 July 2017.

30. Alignments. Available from: https://figshare.com/s/f835735cb66911ff1ffd. Accessed 5 July 2017.

31. Datasets of coalescent-based species tree. Available from: https://figshare.com/s/e5e70c2fd3990e5176d8. Accessed 5 July 2017.

32. Datasets of concatenation based phylogenetic tree. Available from: https://figshare.com/s/8af236b660f61078e40b. Accessed 5 July 2017.

33 Kuo LY, Li FW, Chiu WL, Wang CN. First insights into fern matK phylogeny. Mol Phylogenet Evol. 2011; 59: 556-66.

34. Schneider H. Evolutionary morphology of ferns (monilophytes). Annu Plant Rev. 2013;45:115-40.

35. Christenhusz MJM, Chase M. Trends and concepts in fern classification. Ann Bot. 2014;113:571-94.

36. Schuettpelz E, Pryer KM. Fern phylogeny inferred from 400 leptosporangiate species and three plastid genes. Taxon. 2007;56:1037-50.

37. Bierhorst DW. Morphology of vascular plants. New York: Macmillan; 1971.

38. Schuettpelz E, Schneider H, Huiet L, Windham MD, Pryer KM. A molecular phylogeny of the fern family Pteridaceae: Assessing overall relationships and the affinities of previously unsampled genera. Mol Phylogenet Evol. 2007;44:1172-85.

39. Rothfels CJ, Sundue MA, Kuo L-Y, Larsson A, Kato M, Schuettpelz E, et al. A revised family-level classification for eupolypod II ferns (Polypodiidae: Polypodiales). Taxon. 2012;61:515-33.

40. Mickel JT. The classification and phylogenetic position of the Dennstadtieaceae. In Jeremy AC, Crabbe JA, Thomas BA, editors. The phylogeny and classification of the ferns. London: Academic Press for The Linnean Society of London. 1973. p. 135-44.

41. Taylor TN, Taylor EL, Krings M. Paleobotany: The biology and evolution of fossil plants. 2nd ed. San Diego: Academic Press. 2009. p. 1141

42 Scythe. https://github.com/ucdavis-bioinformatics/scythe. Accessed 5 July 2017.

43. Cox MP, Peterson DA, Biggs PJ. SolexaQA: At-a-glance quality assessment of Illumina second-generation sequencing data. BMC Bioinformatics. 2010. doi:10.1186/1471-2105-11-485.

44. Grabherr MG, Haas BJ, Yassour M, Levin JZ, Thompson DA, Amit I, et al. Full-length transcriptome assembly from RNA-Seq data without a reference genome. Nat Biotechnol. 2011;29:644-U130.

45. van Dongen S. A cluster algorithm for graphs. Technical Report INS-R0010, National Research Institute for Mathematics and Computer Science in the Netherlands. 2000. http://micans.org/mcl/index.html?sec_thesisetc. Accessed 5 July 2017.

46. Hejnol A, Obst M, Stamatakis A, Ott M, Rouse GW, Edgecombe GD, et al. Assessing the root of bilaterian animals with scalable phylogenomic methods. P Roy Soc B-Biol Sci. 2009;276:4261-70.

47. Haas BJ, Papanicolaou A, Yassour M, Grabherr M, Blood PD, Bowden J, et al. *De novo* transcript sequence reconstruction from RNA-Seq: reference generation and analysis with Trinity. Nat Protoc. 2013; doi:10.1038/nprot.2013.084.

48. Katoh K, Standley DM. MAFFT Multiple Sequence Alignment Software Version 7: Improvements in Performance and Usability. Mol Biol Evol. 2013;30:772-80.

49. Talavera G, Castresana J. Improvement of phylogenies after removing divergent and ambiguously aligned blocks from protein sequence alignments. Syst Biol. 2007;56:564-77.

50. Stamatakis A. RAxML: a tool for phylogenetic analysis and post-analysis of large phylogenies. Bioinformatics. 2014;30:1312-3.

51. Dunn CW, Howison M, Zapata F. Agalma: an automated phylogenomics workflow. BMC Bioinformatics. 2013;14:330.

52. Simao FA, Waterhouse RM, Ioannidis P, Kriventseva EV, Zdobnov EM. BUSCO: assessing genome assembly and annotation completeness with single-copy orthologs. Bioinformatics. 2015;31:3210-2.

53. BUSCO results. https://figshare.com/s/bf999173d04b4c311d46. Accessed 5 July 2017

54. Mirarab S, Reaz R, Bayzid MS, Zimmermann T, Swenson MS, Warnow T. ASTRAL: genome-scale coalescent-based species tree estimation. Bioinformatics. 2014;30:I541-8.

55. Seo TK. Calculating bootstrap probabilities of phylogeny using multilocus sequence data. Mol Biol Evol. 2008;25:960-71.

56. ProtienModelselection.pl. https://github.com/stamatak/standard-RAxML/. Accessed 5 July 2017.

57. Rößler R, Galtier J. First Grammatopteris tree ferns from the Southern Hemisphere – new insights in the evolution of the Osmundaceae from the Permian of Brazil. Rev Palaeobot Palynol. 2002;121:205-30.

58. dos Reis M, Yang Z. Approximate Likelihood Calculation on a Phylogeny for Bayesian Estimation of Divergence Times. Mol Biol Evol. 2011;28:2161-72.

59. Maddison WP, Maddison DR. Mesquite: a modular system for evolutionary analysis. 2011. http://mesquiteproject.org. Accessed 5 July 2017.

60. Lewis PO, Olmstead R. A Likelihood Approach to Estimating Phylogeny from Discrete Morphological Character Data. Syst Biol. 2001;50:913-925.

61. Shen H, Jin D, Shu J, Zhou XL, Lei M, Wei R, et al. Supporting data for "Large scale phylogenomic analysis resolves a backbone phylogeny in ferns" *GigaScience* Database. 2017. http://dx.doi.org/10.5524/100353

Figure legends

Figure 1. Topologies (a-f) adapted from published results [5, 12-14, 26, 34]. Branches with support < 75% were shown using dotted lines; and taxa which differ in their phylogeny locations were shown in different colors.

Figure 2. A working flow diagram showing the major processes of data production and analysis in this study. Three major processes are *de novo* transcriptome assembly, one-to-one orthologs prediction, and phylogenetic analysis. The rectangles represent the main results and the ellipses represent the main methods and analysis.

Figure 3. Phylogeny of ferns reconstructed by coalescent-based method using nucleotide sequence with divergence times calculated. Support values for the main phylogeny (a) calculated from Matrix 1/Matrix 2 are listed as percentages; * indicates 100%/100%. Representative leave(s), sporangium and the corresponding lineage are labeled with a same number. Simplified topology (b) shows the main linages as in Figure 1. Species in phylogeny (a) and the corresponding lineage in topology (b) are shown in a same color.

Figure 4. Reconstruction of the evolutionary history of sporangial annulus in ferns. Sampled species with seven types of sporangial annulus are shown in different colours. For each ancient node, percentage of character state of sporangial annulus is shown.

 Click here to download Table Tables.docx

Table 1. Sequencing and assembly information of the transcriptome data. The number of ortholog genes used in Matrix 1 and Matrix 2 were shown.

ID	Species	Clean data (G)	Total reads (clean)	Q30%	Number of contigs	N50 (bp)	Mean (bp)	Genes in Matrix 1	Genes in Matrix 2
RS1	*Pronephrium simplex*	4.7	38045864	91.24	151319	887	581.07	2,168	1,254
RS10	*Antrophyum callifolium*	4.0	32745384	91.76	64107	1819	998.73	2,226	1,305
RS101	*Oleandra musifolia*	4.5	36487068	91.45	37075	1493	919.3	2,093	1,248
RS103	*Woodsia polystichoides*	3.9	31465870	90.91	47812	1348	811.3	2,287	1,310
RS107	*Equisetum diffusum*	4.4	35693238	90.21	88932	1154	655.64	1,811	1,254
RS108	*Oreogrammitis dorsipila*	4.6	37037324	90.57	266540	591	485.1	2,141	1,273
RS11	*Vandenboschia striata*	4.8	38639790	90.3	261724	460	422.76	1,959	1,276
RS111	*Pleurosoriopsis makinoi*	4.8	38983796	90.13	98187	1145	632.29	2,182	1,277
RS112	*Azolla pinnata subsp. asiatica*	4.4	35735206	90.57	78295	1348	777.92	1,418	839
RS114	*Taenitis blechnoides*	4.1	32898682	90.98	70495	1262	711.3	2,186	1,278
RS115	*Gymnogrammitis dareiformis*	3.9	31630988	89.81	119483	569	449.38	1,996	1,220
RS116	*Schizaea dichotoma*	4.5	36668734	89.6	67422	1350	826.92	2,035	1,285
RS119	*Botrychium japonicum*	4.8	38603000	90.28	85236	1477	846.97	1,866	1,283
RS122	*Goniophlebium niponicum*	4.8	38786214	90.82	54152	1663	951.92	2,279	1,300
RS123	*Arthropteris palisotii*	4.4	35646740	91	50700	1454	891.67	2,286	1,311
RS124	*Matteuccia struthiopteris*	4.2	34080998	90.44	57514	1345	776.52	2,290	1,313
RS127	*Salvinia natans*	4.2	33780056	91.17	79393	1379	767.14	1,905	1,173
RS128	*Woodwardia prolifera*	5.1	40967322	91.63	69931	1557	859.72	2,328	1,328
RS14	*Diplazium viridescens*	4.0	32320416	90.46	88236	1434	780.87	2,269	1,310
RS16	*Bolbitis appendiculata*	4.7	37503336	91.66	201426	802	556.39	2,226	1,288
RS17	*Dryopteris pseudocaenopteris*	4.1	33136196	91.23	102751	723	514.92	2,236	1,298
RS18	*Dicranopteris pedata*	4.2	33942120	92.04	74011	1193	684.09	2,031	1,304
RS19	*Haplopteris amboinensis*	4.2	42772168	94.17	47603	1713	1041.8	2,249	1,307
RS21	*Psilotum nudum*	8.5	85199034	93.6	66212	1739	927.19	1,741	1,223
RS24	*Cyclopeltis crenata*	4.6	37158058	91.5	29668	600	491.82	2,146	1,279
RS25	*Asplenium formosae*	4.6	46629754	93.5	73318	1722	989.84	2,273	1,312
RS27	*Lomariopsis spectabilis*	4.1	33233594	91.77	98030	1466	750.42	2,225	1,304
RS28	*Cheiropleuria bicuspis*	5.1	41617294	91.35	99411	1435	832.82	2,022	1,295
RS31	*Plagiogyria japonica*	5.7	46472760	91.92	89532	1258	733.9	2,036	1,222
RS34	*Alsophila podophylla*	4.9	48768608	93.43	66254	1580	904.62	2,195	1,289
RS35	*Histiopteris incisa*	4.3	43115390	93.81	61231	1749	985.03	2,319	1,316
RS36	*Pteris vittata*	4.1	41212858	94.37	76666	1868	1021.13	2,296	1,312
RS37	*Cibotium barometz*	4.1	33263550	91.92	85555	1612	891.87	1,790	1,099
RS38	*Osmunda japonica*	4.1	33485274	92.05	58612	1730	901.28	1,732	1,159
RS39	*Loxogramme chinensis*	3.9	31392952	92.16	84796	1065	651.88	2,240	1,305
RS4	*Microlepia hookeriana*	4.0	40561422	94.49	95951	1610	874.06	2,262	1,301
RS41	*Pteridium aquilinum*	4.6	46157134	93.51	55615	1742	960.37	2,321	1,316

RS42	*Hypolepis punctata*	4.4	43828154	93.56	59717	1371	833.68	2,277	1,308
RS43	*Dicksonia antarctica*	3.9	31210608	91.69	56494	1533	902.96	2,045	1,213
RS45	*Rhachidosorus mesosorus*	4.4	35348994	91.98	80069	1541	835.92	2,300	1,315
RS46	*Drynaria bonii*	4.5	36017548	92.02	68132	1077	643.93	2,176	1,279
RS47	*Platycerium bifurcatum*	4.1	33209740	91.62	40456	1097	694.56	2,148	1,283
RS48	*Angiopteris fokiensis*	4.4	35120302	91.12	57637	1629	932.57	1,917	1,306
RS5	*Diplaziopsis brunoniana*	4.3	34698846	91.35	70184	822	541.31	2,040	1,234
RS50	*Dennstaedtia pilosella*	4.5	45618446	93.63	84813	1582	831.56	2,308	1,313
RS51	*Monachosorum henryi*	4.1	41658504	93.42	87832	1465	803.17	2,255	1,288
RS52	*Acystopteris japonica*	5.5	44662146	91.15	57118	1507	873.59	1,222	677
RS53	*Monachosorum maximowiczii*	4.8	48497004	93.58	101448	1817	899.54	2,257	1,294
RS54	*Dennstaedtia scabra*	5.1	51360716	93.47	92158	1565	845.44	1,818	1,056
RS56	*Arachniodes nigrospinosa*	5.1	50929362	94.47	57168	1623	916.1	2,332	1,319
RS69	*Cheilanthes chusana*	5.2	51851066	94.18	49449	1727	1012.63	2,317	1,324
RS7	*Elaphoglossum mcclurei*	4.1	32800248	92.31	57330	1398	846.79	2,267	1,299
RS70	*Lomagramma matthewii*	4.4	35218876	91.21	65170	1748	947.18	2,258	1,307
RS71	*Osmolindsaea odorata*	4.6	46808646	94.13	113778	1521	845.96	2,257	1,312
RS72	*Aleuritopteris chrysophylla*	4.8	47955674	94.18	61637	1669	929.63	2,307	1,322
RS77	*Marsilea quadrifolia*	4.3	34724432	91.76	65227	1607	930.31	2,188	1,299
RS8	*Humata repens*	4.5	36606746	91.17	68932	1267	690.35	2,264	1,315
RS81	*Tectaria subpedata*	4.2	42539482	94.43	57384	1326	797.83	2,128	1,242
RS84	*Ophioglossum vulgatum*	4.4	35637330	91.77	71821	1226	741.62	1,631	1,179
RS85	*Nephrolepis cordifolia*	5.0	40063236	90.81	55207	1530	842.63	2,302	1,319
RS86	*Microlepia platyphylla*	4.6	46324294	94	74956	1763	945.87	2,267	1,295
RS88	*Lygodium flexuosum*	4.2	34098316	91.44	66751	1514	867.82	2,064	1,296
RS89	*Hypodematium crenatum*	4.1	32711798	91.58	52813	1416	852.57	2,298	1,319
RS90	*Acrostichum aureum*	5.4	43422574	90.69	46189	1729	1043.2	2,303	1,319
RS91	*Adiantum caudatum*	5.1	51062204	94.23	51145	1575	950.49	2,323	1,327
RS92	*Parahemionitis cordata*	4.1	33309450	91.72	47508	1456	894.42	2,306	1,317
RS93	*Microlepia speluncae*	4.4	44124842	94.55	94980	1720	917.59	2,292	1,308
RS97	*Stenochlaena palustris*	4.7	37887642	91.81	58416	1655	945.83	2,300	1,316
RS98	*Ceratopteris thalictroides*	3.9	31741082.0	91.4	74728	1610	912.26	2,231	1,296

Table 2. Inconsistent topologies using different methods and sequences.

Site	Coalescent-based method		Concatenation-based method	
	nucleotide	amino-acid	nucleotide	amino-acid
**A**	**(Anfo,(Pnu,(Ovu,Bja)))**	**(Anfo,(Pnu,(Ovu,Bja)))**	((Pnu,(Ovu,Bja)),(Anfo,#))	((Pnu,(Ovu,Bja)),(Anfo,#))
**B**	**(Cbi,(Dpe,Vst))**	**(Cbi,(Dpe,Vst))**	**(Cbi,(Dpe,Vst))**	((Dpe,Vst),(Cbi,#))
**C**	**(Asfo,(Aja,(Dbr,#)))**	**(Asfo,(Aja,(Dbr,#)))**	**(Asfo,(Aja,(Dbr,#)))**	(Asfo,((Aja,Dbr),#))
**D**	**(Dvi,(Mst,(Spa,Wpr)))**	((Dvi,Mst),(Spa,Wpr))	**(Dvi,(Mst,(Spa,Wpr)))**	**(Dvi,(Mst,(Spa,Wpr)))**
**E**	**(Bap,(Emc,Lma))**	(Emc,(Bap,Lma))	**(Bap,(Emc,Lma))**	(Emc,(Bap,Lma))
**F**	**(Nco,((Tsu,Apa),#))**	(Nco,(Tsu,(Apa,#)))	**(Nco,((Tsu,Apa),#))**	**(Nco,((Tsu,Apa),#))**

(A) Anfo: *Angiopteris fokiensis*, Pnu: *Psilotum nudum*, Ovu: *Ophioglossum vulgatum*, Bja: *Botrychium japonicum*; (B) Cbi: Cheiropleuria bicuspis, Dpe: *Dicranopteris pedata*, Vst: *Vandenboschia striata*; (C) Asfo: Asplenium formosae, Aja: *Acystopteris japonica*, Dbr: *Diplaziopsis brunoniana*; (D) Dvi: *Diplazium viridescens*, Mst: *Matteuccia struthiopteris*, Spa: *Stenochlaena palustris*, Wpr: *Woodwardia prolifera*; (E) Bap: *Bolbitis appendiculata*, Emc: Elaphoglossum mcclurei, Lma: *Lomagramma matthewii*; (F) Nco: *Nephrolepis cordifolia*, Tsu: *Tectaria subpedata*, Apa: *Arthropteris palisotii*. # indicates other sampled species within this lineage. Topologies consistent with the one yielded from coalescent-based method and nucleotide sequences are shown in bold.

Figure 1 Click here to download Figure Figure 1.bmp

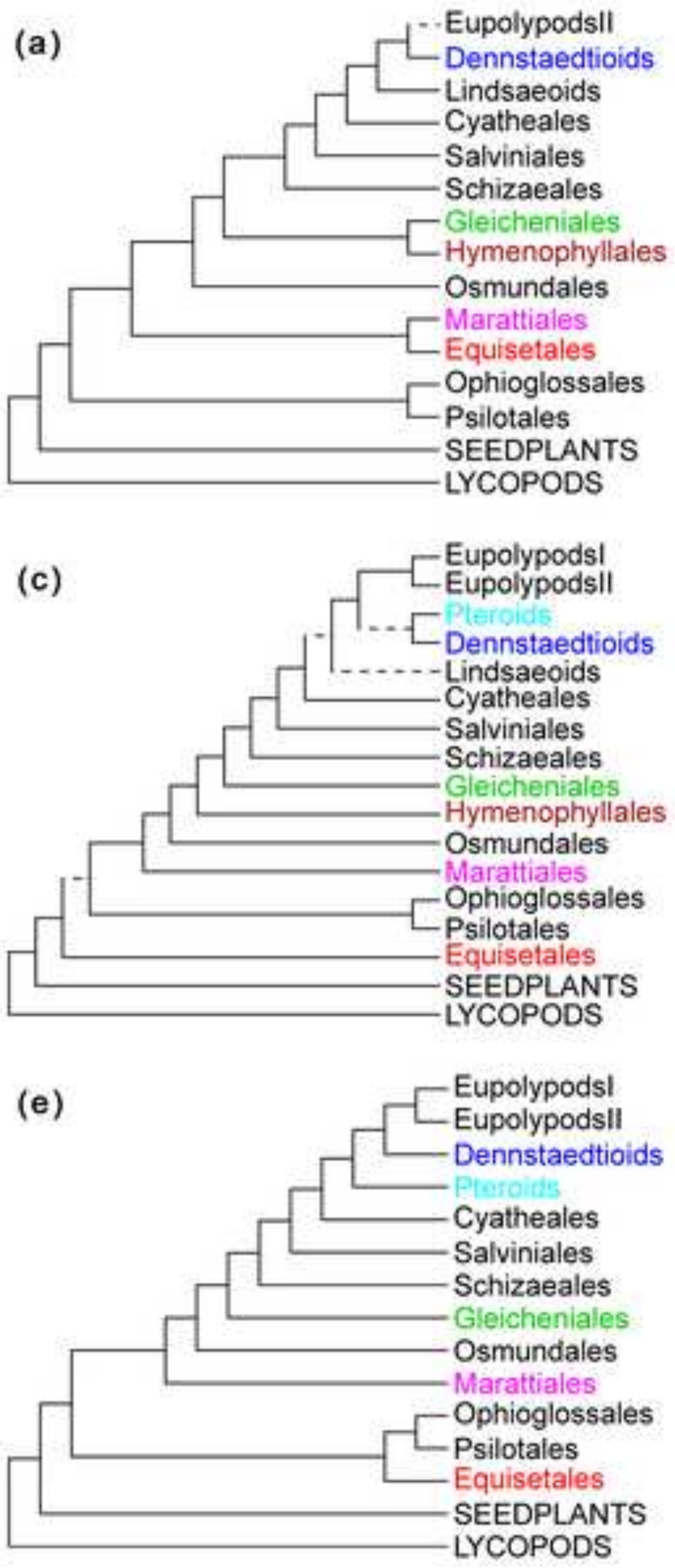

Figure 2

Click here to download Figure Figure 2.bmp

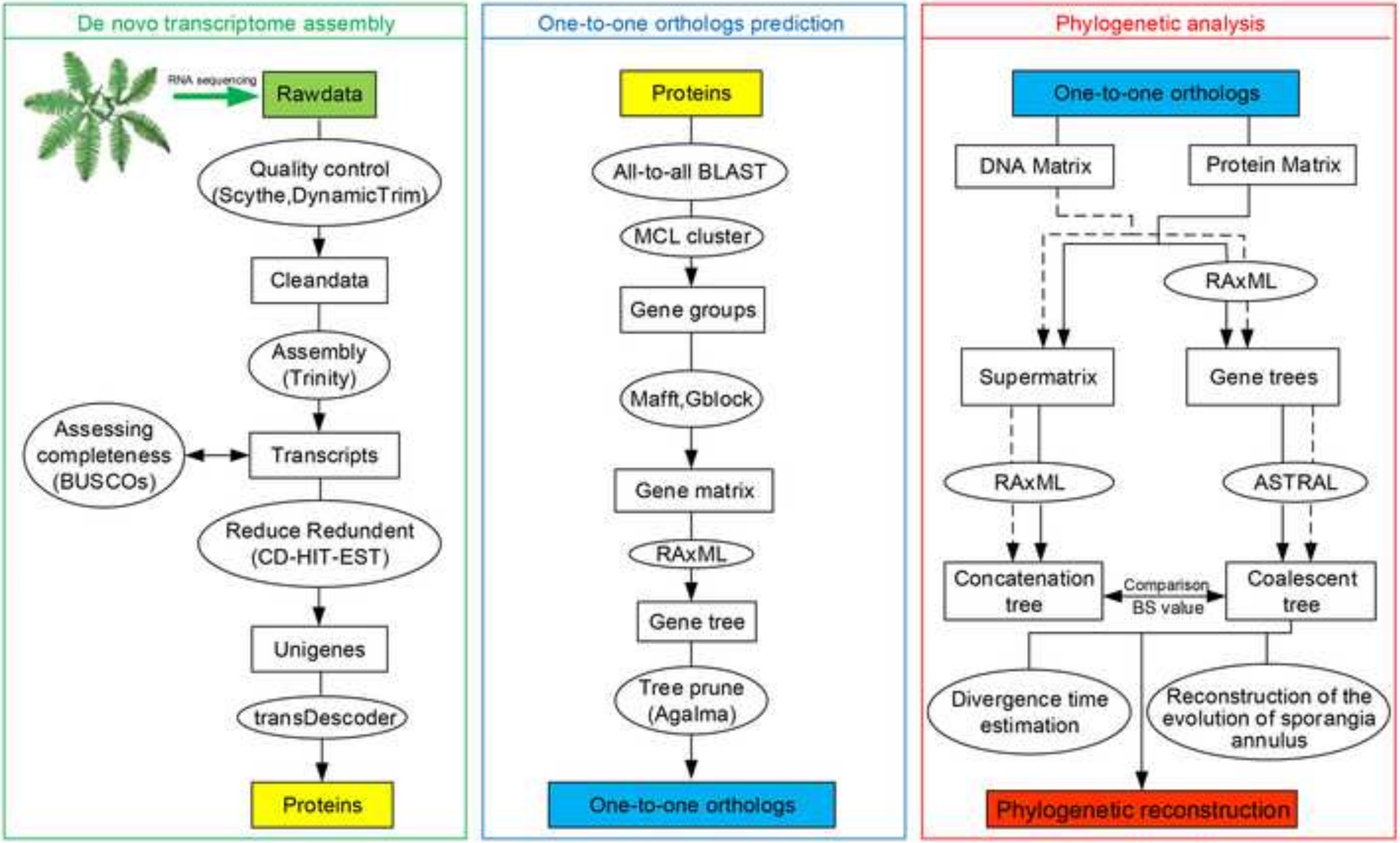

Figure 3

Click here to download Figure Figure 3.bmp

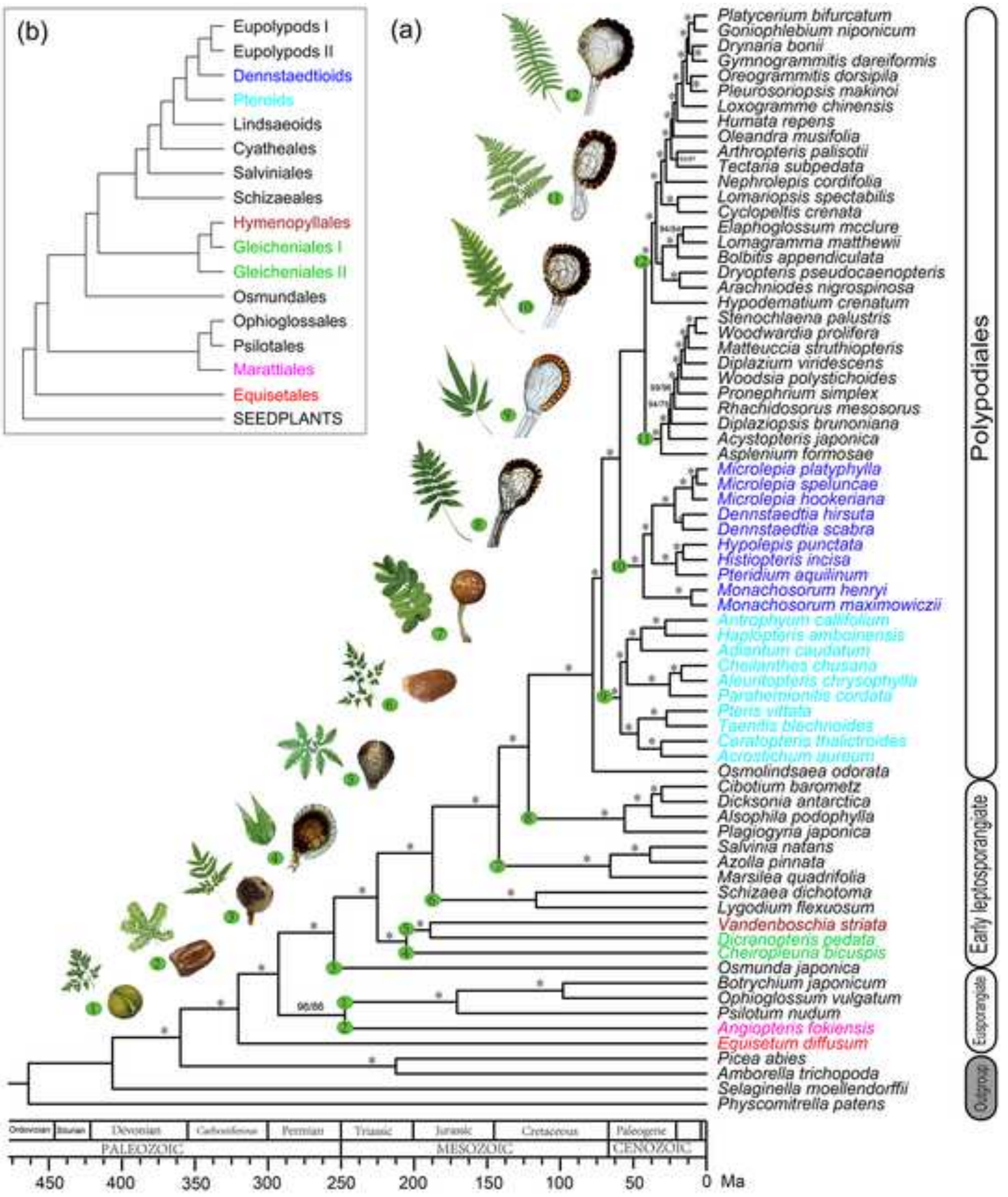

Figure 4

Click here to download Figure Figure 4_0915.bmp

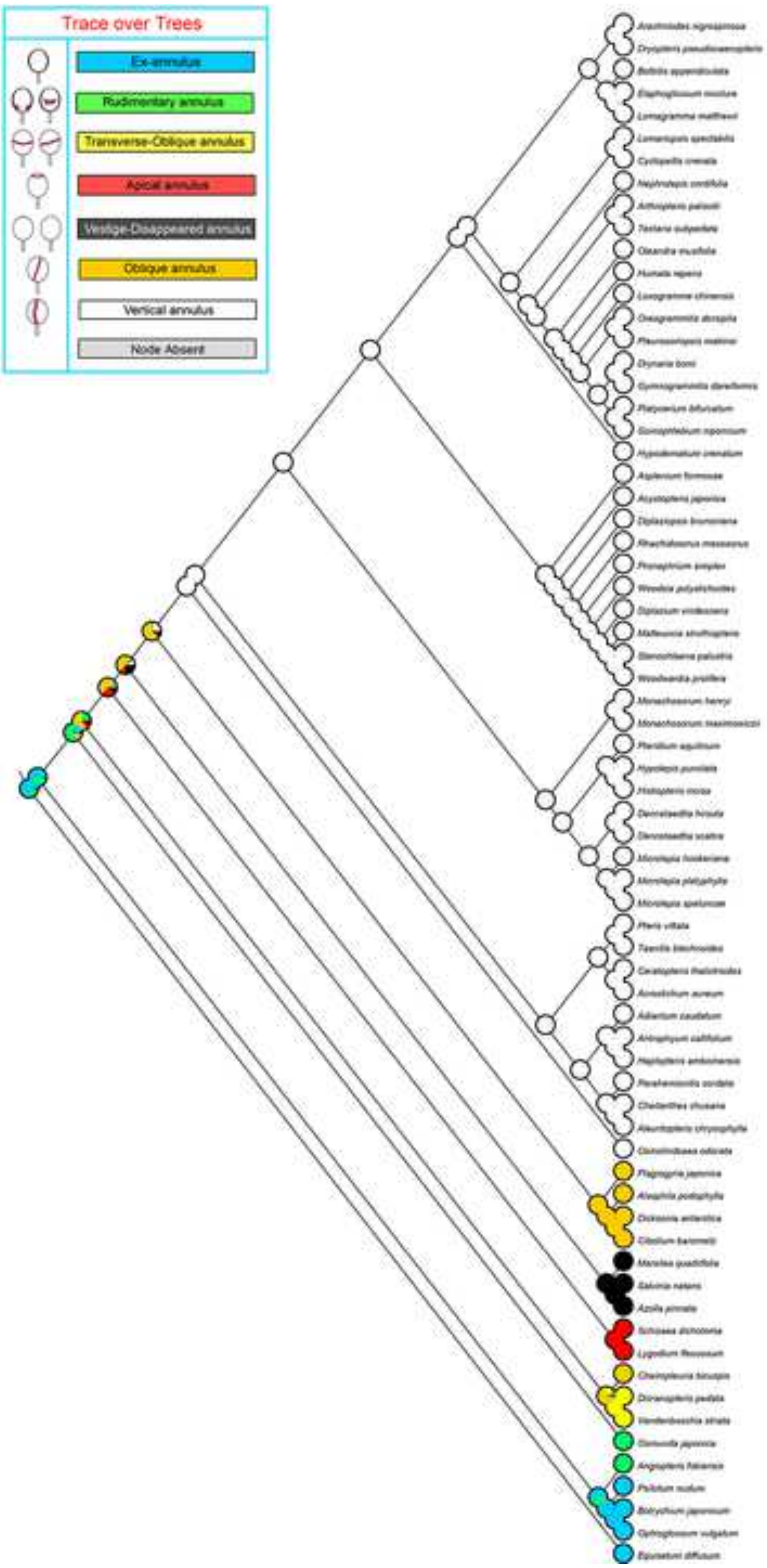

Click here to access/download
Supplementary Material
Supplementary information_0915.docx

 Click here to download Personal Cover Cover letter_915.docx

Yue-Hong Yan, Professor
Shanghai Chenshan Plant Science Research Center,
Chinese Academy of Sciences & Shanghai Chenshan Botanical Garden
3888 Chenhua Road, Shanghai 201602, China
Tel: +86-21-37792288-903
Fax: +86-21-67657811
Email: yhyan@sibs.ac.cn
September 15, 2017

Dear Editor for GigaScience:

We have revised a manuscript entitled "**Large scale phylogenomic analysis resolves a backbone phylogeny in ferns**" (formerly manuscript number: **GIGA-D-17-00009**) for your consideration to be published in GigaScience. The materials in the manuscript have not been published, nor are under consideration for publication elsewhere.

All the suggestions and comments by the reviewer have been adopted in our revised manuscript. We have revised the whole manuscript carefully to avoid language errors.

At present, we are waiting for the data curator to contact us. When the GigaDB entry is prepared, we will include a citation to the GigaDB dataset to the manuscript's reference list, in the data availability section, and elsewhere in the manuscript when appropriate.

Thank you very much for handling our manuscript. I am looking forward to hearing your decision soon.

Sincerely yours,
Yue-Hong Yan
