# Supplementary material for: Large-scale phylogenomic analysis resolves a backbone phylogeny in ferns
Source: Gigascience. 2017 Nov 24;7(2):gix116. doi: 10.1093/gigascience/gix116 (PMC5795342; doi:10.1093/gigascience/gix116)
Supplement: Supplement Files [file gix116_supp.docx]

**Supplementary information**

**Table S1. Sampling information including sample ID, species, specimen ID, sampling site, tissue, time, and the biosample ID.**

| **Sample ID** | **Species** | **Specimen ID** | **Sampling site** | **Tissue** | **Time** | **Biosample ID** |
| --- | --- | --- | --- | --- | --- | --- |
| RS-1 | *Pronephrium simplex* | RS-1 | Hainan,China | S, T | 2014.3.11 | SAMN03575942 |
| RS-4 | *Microlepia hookeriana* | RS-4 | Hainan,China | T | 2014.3.12 | SAMN03575887 |
| RS-5 | *Diplaziopsis brunoniana* | RS-5 | Hainan,China | S | 2014.3.12 | SAMN03575894 |
| RS-7 | *Elaphoglossum mcclurei* | RS-7 | Hainan,China | S | 2014.3.12 | SAMN03575900 |
| RS-8 | *Humata repens* | RS-8 | Hainan,China | T | 2014.3.12 | SAMN03575882 |
| RS-10 | *Antrophyum callifolium* | RS-10 | Hainan,China | S | 2014.3.12 | SAMN03575934 |
| RS-11 | *Vandenboschia striata* | RS-11 | Hainan,China | S | 2014.3.12 | SAMN03575903 |
| RS-14 | *Diplazium viridescens* | RS-14 | Hainan,China | S | 2014.3.12 | SAMN03575876 |
| RS-16 | *Bolbitis appendiculata* | RS-16 | Hainan,China | S, T | 2014.3.12 | SAMN03575898 |
| RS-17 | *Dryopteris pseudocaenopteris* | RS-17 | Hainan,China | S, T | 2014.3.12 | SAMN03575896 |
| RS-18 | *Dicranopteris pedata* | RS-18 | Hainan,China | S, T | 2014.3.12 | SAMN03575902 |
| RS-19 | *Haplopteris amboinensis* | RS-19 | Hainan,China | S, T | 2014.3.12 | SAMN03575935 |
| RS-21 | *Psilotum nudum* | RS-21 | Hainan,China | S, T | 2014.3.13 | SAMN03575925 |
| RS-24 | *Cyclopeltis crenata* | RS-24 | Hainan,China | T | 2014.3.13 | SAMN03575906 |
| RS-25 | *Asplenium formosae* | RS-25 | Hainan,China | S, T | 2014.3.13 | SAMN03575875 |
| RS-27 | *Lomariopsis spectabilis* | RS-27 | Hainan,China | T | 2014.3.11 | SAMN03575907 |
| RS-28 | *Cheiropleuria bicuspis* | 5214 | Guangdong,China | S, T | 2014.3.5 | SAMN03575895 |
| RS-31 | *Plagiogyria japonica* | 5220 | Guangdong,China | S, T | 2014.3.5 | SAMN03575917 |
| RS-34 | *Alsophila podophylla* | RS-34 | Green house | S, T | 2013.3.24 | SAMN03575880 |
| RS-35 | *Histiopteris incisa* | RS-35 | Green house | T | 2013.3.24 | SAMN03575892 |
| RS-36 | *Pteris vittata* | RS-36 | Green house | S, T | 2013.3.24 | SAMN03575926 |
| RS-37 | *Cibotium barometz* | RS-37 | Green house | S, T | 2013.3.24 | SAMN03575879 |
| RS-38 | *Osmunda japonica* | RS-38 | Shanghai,China | S | 2013.3.26 | SAMN03575916 |
| RS-39 | *Loxogramme chinensis* | SG063 | Fujian,China | S, T | 2014.4.11 | SAMN03575924 |
| RS-41 | *Pteridium aquilinum* | RS-41 | Shanghai,China | T | 2014.4.16 | SAMN03575891 |
| RS-42 | *Hypolepis punctata* | RS-42 | Green house | S, T | 2014.4.16 | SAMN03575890 |
| RS-43 | *Dicksonia antarctica* | RS-43 | Green house | T | 2014.4.16 | SAMN03575893 |
| RS-45 | *Rhachidosorus mesosorus* | RS-45 | Green house | S | 2014.4.16 | SAMN03575936 |
| RS-46 | *Drynaria bonii* | RS-46 | Green house | S, T | 2014.4.16 | SAMN03575922 |
| RS-47 | *Platycerium bifurcatum* | RS-47 | Green house | T | 2014.4.16 | SAMN03575923 |
| RS-48 | *Angiopteris fokiensis* | RS-48 | Green house | T | 2014.4.16 | SAMN03575909 |
| RS-50 | *Dennstaedtia hirsuta* | RS-50 | Hunan,China | S, T | 2014.4.16 | SAMN03575885 |
| RS-51 | *Monachosorum henryi* | RS-51 | Hunan,China | S, T | 2014.4.16 | SAMN03575884 |
| RS-52 | *Acystopteris japonica* | RS-52 | Hunan,China | S | 2014.4.16 | SAMN03575881 |
| RS-53 | *Monachosorum maximowiczii* | RS-53 | Hunan,China | S, T | 2014.4.16 | SAMN03575883 |
| RS-54 | *Dennstaedtia scabra* | RS-54 | Guangxi,China | S | 2014.4.17 | SAMN03575886 |
| RS-56 | *Arachniodes nigrospinosa* | RS-56 | Guangxi,China | S | 2014.4.18 | SAMN03575897 |
| RS-69 | *Cheilanthes chusana* | RS-69 | Guangxi,China | S | 2014.4.23 | SAMN03575930 |
| RS-70 | *Lomagramma matthewii* | RS-70 | Guangxi,China | S, T | 2014.4.23 | SAMN03575899 |
| RS-71 | *Osmolindsaea odorata* | RS-71 | Guangxi,China | S | 2014.4.21 | SAMN03575905 |
| RS-72 | *Aleuritopteris chrysophylla* | RS-72 | Guangxi,China | S | 2014.4.21 | SAMN03575929 |
| RS-77 | *Marsilea quadrifolia* | RS-77 | Shanghai,China | T | 2014.4.30 | SAMN03575910 |
| RS-81 | *Tectaria subpedata* | 13553 | Guangxi,China | S | 2014.4.26 | SAMN03575940 |
| RS-84 | *Ophioglossum vulgatum* | RS-84 | Guangdong,China | S, T | 2014.5.9 | SAMN03575915 |
| RS-85 | *Nephrolepis cordifolia* | 13679 | Guangdong,China | T | 2014.5.9 | SAMN03575911 |
| RS-86 | *Microlepia platyphylla* | RS-86 | Green house | T | 2014.5.11 | SAMN03575888 |
| RS-88 | *Lygodium flexuosum* | RS-88 | Green house | S | 2014.5.15 | SAMN03575908 |
| RS-89 | *Hypodematium crenatum* | RS-89 | Green house | S | 2014.5.15 | SAMN03575904 |
| RS-90 | *Acrostichum aureum* | RS-90 | Green house | S | 2014.5.15 | SAMN03575928 |
| RS-91 | *Adiantum caudatum* | RS-91 | Green house | S | 2014.5.15 | SAMN03575931 |
| RS-92 | *Parahemionitis cordata* | RS-92 | Green house | S, T | 2014.5.15 | SAMN03575933 |
| RS-93 | *Microlepia speluncae* | RS-93 | Green house | S | 2014.5.15 | SAMN03575889 |
| RS-97 | *Stenochlaena palustris* | RS-97 | Green house | S, T | 2014.5.15 | SAMN03575877 |
| RS-98 | *Ceratopteris thalictroides* | RS-98 | Zhejiang,China | S | 2014.5.22 | SAMN03575932 |
| RS-101 | *Oleandra musifolia* | RS-101 | Green house | T | 2014.5.15 | SAMN03575912 |
| RS-103 | *Woodsia polystichoides* | RS-103 | Anhui,China | S | 2014.6.20 | SAMN03575943 |
| RS-107 | *Equisetum diffusum* | RS-107 | Sichuan,China | T | 2014.6.29 | SAMN03575901 |
| RS-108 | *Oreogrammitis dorsipila* | RS-108 | Hunan,China | S | 2014.7.1 | SAMN03575921 |
| RS-111 | *Pleurosoriopsis makinoi* | RS-111 | Hunan,China | S | 2014.7.8 | SAMN03575918 |
| RS-112 | *Azolla pinnata* | RS-112 | Shanghai,China | T | 2014.7.9 | SAMN03575938 |
| RS-114 | *Taenitis blechnoides* | RS-114 | Hainan,China | S | 2014.7.12 | SAMN03575927 |
| RS-115 | *Gymnogrammitis dareiformis* | RS-115 | Hainan,China | S | 2014.7.12 | SAMN03575919 |
| RS-116 | *Schizaea dichotoma* | RS-116 | Hainan,China | S | 2014.7.12 | SAMN03575939 |
| RS-119 | *Botrychium japonicum* | RS-117 | Green house | S, T, Y | 2014.8.1 | SAMN03575914 |
| RS-122 | *Goniophlebium niponicum* | RS-122 | Green house | T | 2014.8.1 | SAMN03575920 |
| RS-123 | *Arthropteris palisotii* | RS-123 | Green house | T | 2014.8.1 | SAMN03575941 |
| RS-124 | *Matteuccia struthiopteris* | RS-124 | Shanghai,China | T | 2014.8.1 | SAMN03575913 |
| RS-127 | *Salvinia natans* | RS-127 | Shanghai,China | T | 2014.8.1 | SAMN03575937 |
| RS-128 | *Woodwardia prolifera* | RS-128 | Green house | S | 2014.9.25 | SAMN03575878 |

S: sporophyll; T: trophophyll; Y: young frond.

**Table S2. The results of BUSCO comparison using transcriptome assemblies of 69 fern species.**

| **Sample ID** | **Species** | **Total gene number in eukaryote** | **Completed number** | **Fragmented number** | **Missing number** | **Completed rate** |
| --- | --- | --- | --- | --- | --- | --- |
| RS-1 | *Pronephrium simplex* | 303 | 283 | 14 | 6 | 93.40% |
| RS-4 | *Microlepia hookeriana* | 303 | 286 | 11 | 0 | 94.40% |
| RS-5 | *Diplaziopsis brunoniana* | 303 | 187 | 90 | 26 | 61.80% |
| RS-7 | *Elaphoglossum mcclurei* | 303 | 280 | 18 | 5 | 92.40% |
| RS-8 | *Humata repens* | 303 | 271 | 23 | 9 | 89.40% |
| RS-10 | *Antrophyum callifolium* | 303 | 279 | 16 | 8 | 92.10% |
| RS-11 | *Vandenboschia striata* | 303 | 251 | 47 | 5 | 82.80% |
| RS-14 | *Diplazium viridescens* | 303 | 285 | 11 | 7 | 94.00% |
| RS-16 | *Bolbitis appendiculata* | 303 | 281 | 21 | 1 | 92.70% |
| RS-17 | *Dryopteris pseudocaenopteris* | 303 | 264 | 27 | 12 | 87.10% |
| RS-18 | *Dicranopteris pedata* | 303 | 272 | 25 | 6 | 89.80% |
| RS-19 | *Haplopteris amboinensis* | 303 | 287 | 12 | 4 | 94.80% |
| RS-21 | *Psilotum nudum* | 303 | 290 | 9 | 4 | 95.70% |
| RS-24 | *Cyclopeltis crenata* | 303 | 72 | 64 | 167 | 23.80% |
| RS-25 | *Asplenium formosae* | 303 | 282 | 8 | 13 | 93.10% |
| RS-27 | *Lomariopsis spectabilis* | 303 | 275 | 20 | 8 | 90.80% |
| RS-28 | *Cheiropleuria bicuspis* | 303 | 293 | 6 | 4 | 96.70% |
| RS-31 | *Plagiogyria japonica* | 303 | 279 | 16 | 8 | 92.10% |
| RS-34 | *Alsophila podophylla* | 303 | 289 | 7 | 7 | 95.40% |
| RS-35 | *Histiopteris incisa* | 303 | 290 | 9 | 4 | 95.70% |
| RS-36 | *Pteris vittata* | 303 | 288 | 11 | 4 | 95.10% |
| RS-37 | *Cibotium barometz* | 303 | 285 | 9 | 9 | 94.10% |
| RS-38 | *Osmunda japonica* | 303 | 289 | 4 | 10 | 95.40% |
| RS-39 | *Loxogramme chinensis* | 303 | 257 | 35 | 11 | 84.80% |
| RS-41 | *Pteridium aquilinum* | 303 | 295 | 5 | 3 | 97.40% |
| RS-42 | *Hypolepis punctata* | 303 | 253 | 38 | 12 | 83.50% |
| RS-43 | *Dicksonia antarctica* | 303 | 279 | 12 | 12 | 92.10% |
| RS-45 | *Rhachidosorus mesosorus* | 303 | 278 | 19 | 6 | 91.80% |
| RS-46 | *Drynaria bonii* | 303 | 257 | 34 | 12 | 84.80% |
| RS-47 | *Platycerium bifurcatum* | 303 | 220 | 57 | 26 | 72.60% |
| RS-48 | *Angiopteris fokiensis* | 303 | 286 | 11 | 6 | 94.40% |
| RS-50 | *Dennstaedtia pilosella* | 303 | 294 | 7 | 2 | 97.10% |
| RS-51 | *Monachosorum henryi* | 303 | 283 | 9 | 11 | 93.40% |
| RS-52 | *Acystopteris japonica* | 303 | 282 | 12 | 9 | 93.10% |
| RS-53 | *Monachosorum maximowiczii* | 303 | 294 | 5 | 4 | 97.00% |
| RS-54 | *Dennstaedtia scabra* | 303 | 287 | 10 | 6 | 94.70% |
| RS-56 | *Arachniodes nigrospinosa* | 303 | 287 | 10 | 6 | 94.70% |
| RS-69 | *Cheilanthes chusana* | 303 | 284 | 12 | 7 | 93.80% |
| RS-70 | *Lomagramma matthewii* | 303 | 279 | 11 | 13 | 92.10% |
| RS-71 | *Osmolindsaea odorata* | 303 | 286 | 9 | 8 | 94.40% |
| RS-72 | *Aleuritopteris chrysophylla* | 303 | 286 | 7 | 10 | 94.30% |
| RS-77 | *Marsilea quadrifolia* | 303 | 277 | 16 | 10 | 91.40% |
| RS-81 | *Tectaria subpedata* | 303 | 266 | 29 | 8 | 87.80% |
| RS-84 | *Ophioglossum vulgatum* | 303 | 283 | 13 | 7 | 93.40% |
| RS-85 | *Nephrolepis cordifolia* | 303 | 272 | 20 | 11 | 89.80% |
| RS-86 | *Microlepia platyphylla* | 303 | 285 | 14 | 4 | 94.00% |
| RS-88 | *Lygodium flexuosum* | 303 | 281 | 18 | 4 | 92.70% |
| RS-89 | *Hypodematium crenatum* | 303 | 277 | 22 | 4 | 91.40% |
| RS-90 | *Acrostichum aureum* | 303 | 284 | 13 | 6 | 93.80% |
| RS-91 | *Adiantum caudatum* | 303 | 287 | 12 | 4 | 94.80% |
| RS-92 | *Parahemionitis cordata* | 303 | 281 | 18 | 4 | 92.70% |
| RS-93 | *Microlepia speluncae* | 303 | 290 | 10 | 3 | 95.70% |
| RS-97 | *Stenochlaena palustris* | 303 | 285 | 12 | 6 | 94.10% |
| RS-98 | *Ceratopteris thalictroides* | 303 | 289 | 7 | 7 | 95.30% |
| RS-101 | *Oleandra musifolia* | 303 | 261 | 27 | 15 | 86.20% |
| RS-103 | *Woodsia polystichoides* | 303 | 257 | 33 | 13 | 84.80% |
| RS-107 | *Equisetum diffusum* | 303 | 284 | 13 | 6 | 93.80% |
| RS-108 | *Oreogrammitis dorsipila* | 303 | 280 | 21 | 2 | 92.40% |
| RS-111 | *Pleurosoriopsis makinoi* | 303 | 276 | 22 | 5 | 91.10% |
| RS-112 | *Azolla pinnata subsp. asiatica* | 303 | 284 | 15 | 4 | 93.70% |
| RS-114 | *Taenitis blechnoides* | 303 | 258 | 33 | 12 | 85.10% |
| RS-115 | *Gymnogrammitis dareiformis* | 303 | 170 | 96 | 37 | 56.10% |
| RS-116 | *Schizaea dichotoma* | 303 | 291 | 9 | 3 | 96.00% |
| RS-119 | *Botrychium japonicum* | 303 | 291 | 8 | 4 | 96.00% |
| RS-122 | *Goniophlebium niponicum* | 303 | 292 | 8 | 3 | 96.30% |
| RS-123 | *Arthropteris palisotii* | 303 | 267 | 25 | 11 | 88.10% |
| RS-124 | *Matteuccia struthiopteris* | 303 | 275 | 21 | 7 | 90.80% |
| RS-127 | *Salvinia natans* | 303 | 276 | 15 | 12 | 91.10% |
| RS-128 | *Woodwardia prolifera* | 303 | 283 | 12 | 8 | 93.40% |

**
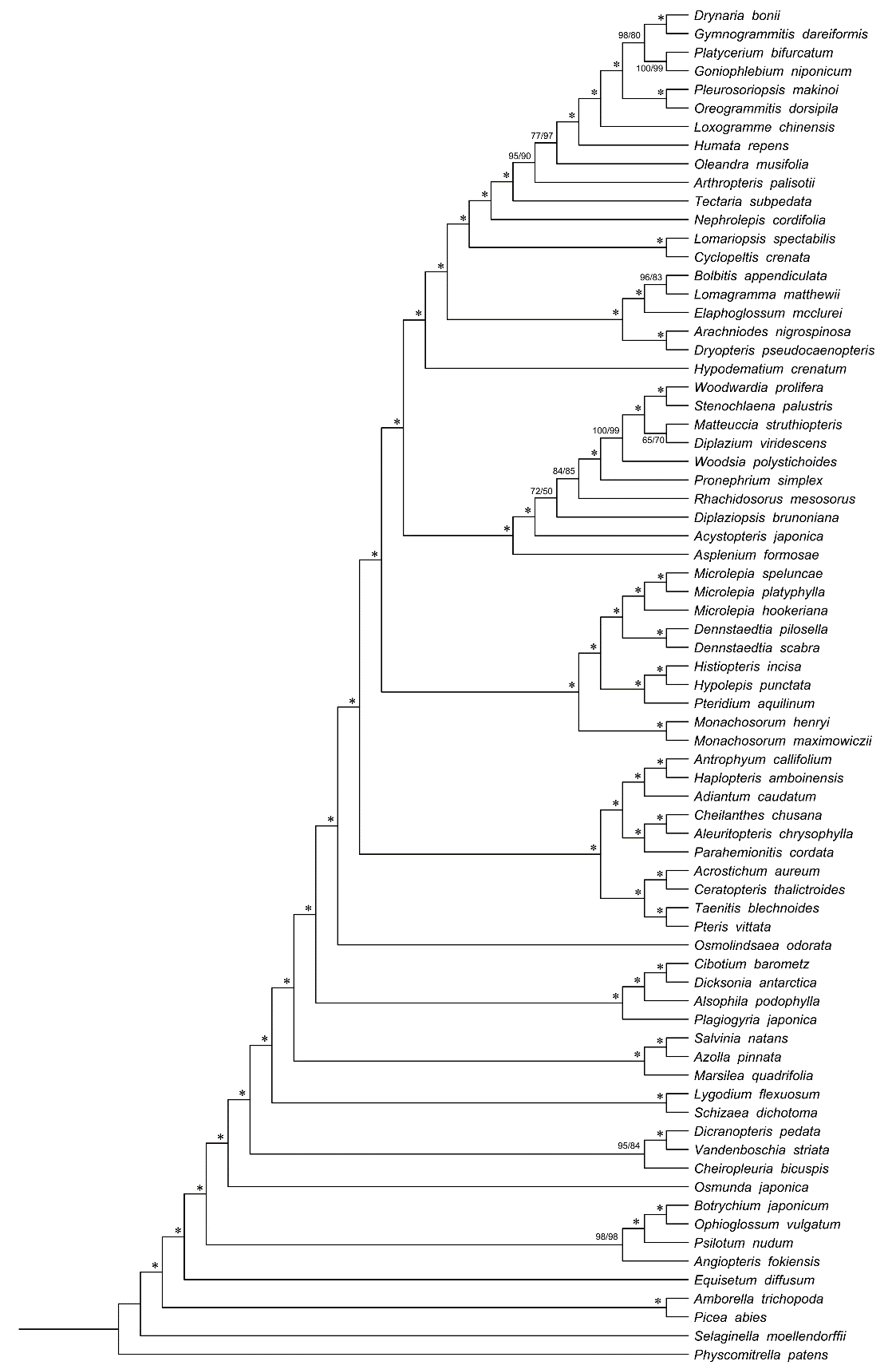
**

**Figure S1. Phylogeny of ferns estimated by applying coalescent-based method to amino-acid sequences.** Node labels as “n1/n2” indicate supporting values of Matrix 1/Matrix 2, * indicates 100%/100%.

**
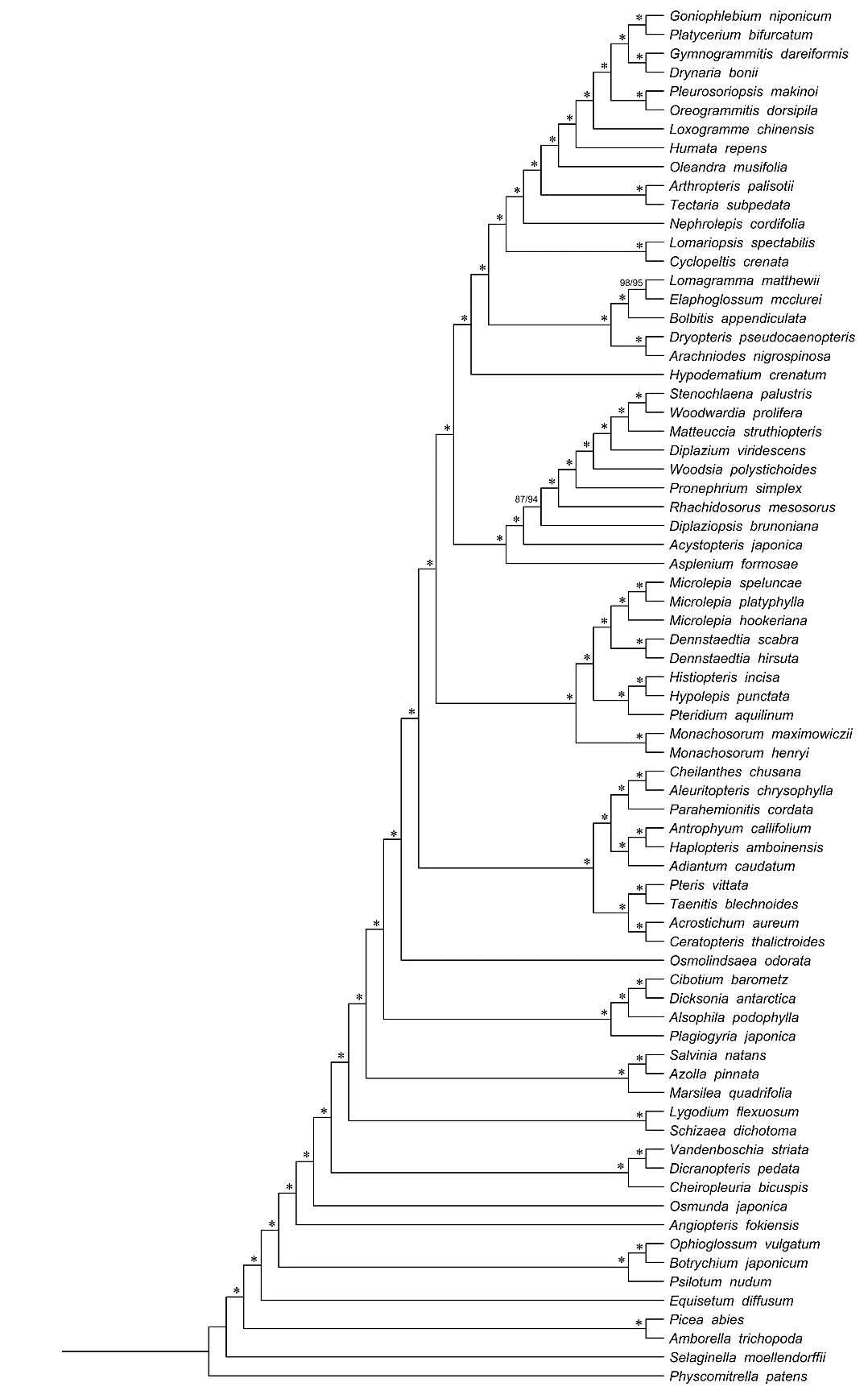
**

**Figure S2. Phylogeny of ferns estimated by applying concatenation-based method to nucleotide sequences.** Node labels as “n1/n2” indicate supporting values of Matrix 1/Matrix 2, * indicates 100%/100%.

**
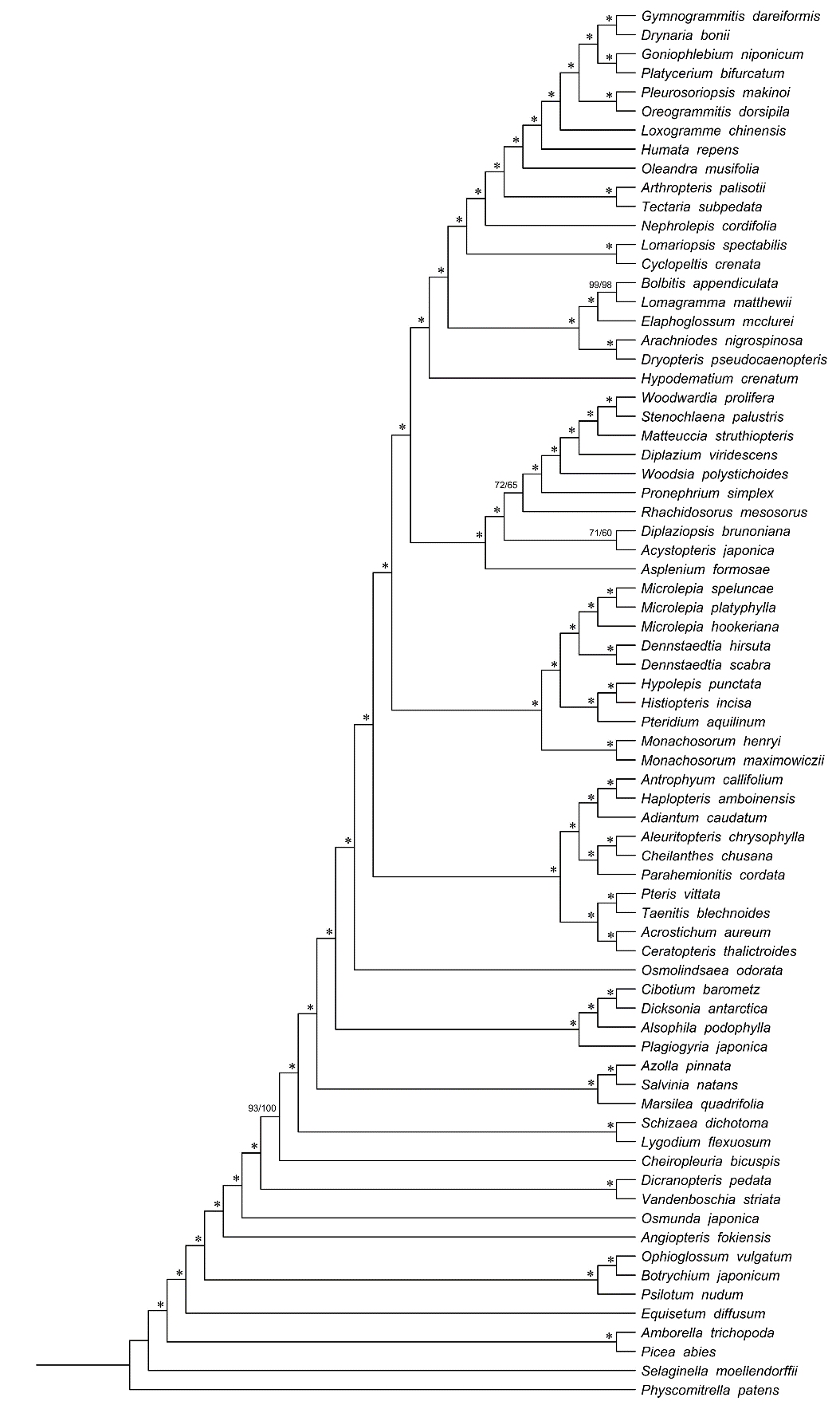
**

**Figure S3. Phylogeny of ferns estimated by applying concatenation-based method to amino-acid sequences.** Node labels as “n1/n2” indicate supporting values of Matrix 1/Matrix 2, * indicates 100%/100%.
