# Supplementary material for: Large-scale phylogenomic analysis resolves a backbone phylogeny in ferns
Source: Gigascience. 2017 Nov 24;7(2):gix116. doi: 10.1093/gigascience/gix116 (PMC5795342; doi:10.1093/gigascience/gix116)
Supplement: Response-to-Reviewer-Comments_Original-Submission.pdf [file gix116_response-to-reviewer-comments_original-submission.pdf]

Author's Response To Reviewer Comments

Reviewer #1:
page 3, line 60, change "basing on" to "based on"
R: "Basing on" has been changed to "based on" as suggested (page 3, line 61).

page 4, line 66, change "basing on" to "based on", and change "analysis" to "analyses"
R: "Basing on" has been changed to "based on" , "analysis" has been changed to "analyses" (page 4, line 67).

page 4, line 74, change "close" to "closely"
R: "Close" has been changed to "closely" (page 4 line 77).

page 5, line 91, change "Schezaeles" to "Schizaeales"
R: "Schezaeles" has been changed to "Schizaeales" (page 5, line 92).

page 5, line 101, change "Genbank" to "NCBI"
R: "Genbank" has been changed to "NCBI" (page 5, line 102).

page 5, line 103, change "filtration" to "filtering"
R: "Filtration" has been changed to "filtering" (page5, line 104).

page 5, line 107, change "lineage" to "lineages"
R: "Lineage" has been changed to "lineages" (page5, line 108).

page 6, line 127-130, suggested wording: "For each combination of reconstruction methods (coalescent-based or concatenation-based) and sequence types (nucleotides or amino acids), Matrix 1 and Matrix 2 always yielded the same topology. In general, the four cladograms (Figure 3, Figure S1, S2, S3) from a combination of methods and sequence types are consistent except six positions (Table 2)."
R: It has been changed as suggested (page 6, line 127-131), thank you.

page 6, line 130-132, what do you mean by "most agreed"?
R: This sentence has been changed as "Among the topologies, the one estimated by applying coalescent-based method to nucleotide sequence (Figure 3) and the one applying concatenation-based method (Figure S2) are most congruent"(page 6, line 131-133).

page 6, line 133, change "evolution" to "evolutionary"
R: "Evolution" has been changed to "evolutionary" (page 6, line 134).

page 7, line 149, change "among close related taxa" to "at shallow phylogenetic scale"
R: It has been changed as suggested (page 6, line 150), thank you.

page 7, line 152-154, suggested wording: "..., and are often the controversial nodes from past studies based on different genes, we suggest such inconsistency might be caused partially by LIS

and reticulate evolution."
R: It has been changed as suggested (page 7, line 153-154), thank you.

page 8, line 158-159, Rothfels et al (2015) is not the first to report Equisetum being sister to the rest. See Rai and Graham (2010, AJB), and Kuo et al (2011, MPE). Also change "basing" to "based"
R: It has been changed as “This topology confirmed the results reported by Rai & Graham [12], and Kuo et al. [33] based on plastid genes, and has been accepted by the PPG I [3] in 2016” (page 8 , line 158-160).

page 8, line 172, change "view of mainstream" to "mainstream"
R: "View of mainstream" has been changed to "mainstream" (page 8, line 175).

page 9, line 179, no need to say "forking ferns" and "filmy ferns" again here.
R: "Forking ferns" and "filmy ferns" have been deleted here, thank you (page 9, line 182).

page 9, line 181, change "Differently" to "On the other hand"
R: "Differently" has been changed to "On the other hand" (page 9, line 184).

page 9, line 185, change "may form a sister lineage to" to "may be sister to"
R: "May form a sister lineage to" has been changed to "may be sister to" (page 9, line 187).

page 9, line 186, change "the Gleicheniales order" to "Gleicheniales"
R: "The Gleicheniales order" has been changed to "Gleicheniales" (page 9, line 189).

page 9, line 195, remove "the disputation of inner"
R: "The disputation of inner" has been removed (page 9, line 197).

page 9, line 200, change "in agree with" to "in agreement with", and references are needed for this sentence.
R: It has been changed as “Our results showed that eupolypods are divided into two major lineages, eupolypods I and eupolypods II in agreement with the consensus opinion [3]” (page 10, line 201-202).

page 10, line 202, change "new" to "different"
R: "New to" has been changed to "different from" (page 10, line 204).

page 10, line 206, both "phylogram" and "cladogram" are used in this manuscript, and in a seemly interchangeable way. I'd prefer "topology".
R: Both "phylogram" and "cladogram" have been changed to “topology” in this manuscript. Thank you. (e.g., page 10, line 208).

page 10, line 208, change "more close to" to "more closely related to"
R: "More close to" has been changed to "more closely related to" (Page 10, line 210).

page 10, line 206-216, NO MORE USE OF "PRIMITIVE"!!! Everthing extant is equally

"advanced".
R: Although extant species are equally “advanced”, their characters can be “primitive”, “original”, or “derived”. This sentence has been changed as “In Pteridaceae, the unstable structure of spherical sporangia, including variable annulus and short sporangial stalk, indicates these characters of sporangia are relatively original and are close to those with oblique annulus in early leptosporangiate. We also noticed that the characters of spherical sporangia with slightly oblique annulus in Monachosorum should be more primitive than the flattened sporangia with typical vertical annulus in other genera of Dennstaedtaceae” (page 10, line 211-216).

page 10-11, "The evolution of sporangia annulus in ferns". I'm still having trouble understanding how the authors deduce the "routes" of annulus evolution. The "two subroutes" is particularly confusing - it would only make sense if Schizaeales and Salviniales are monophyletic, which they are not. And again, the ladderized thinking - with Polypodiales having the advanced, final states while the others being the primitive intermediates - is not correct.
R: Thanks to your advice. In the former manuscript, the "two subroutes" was indeed incongruent with the reconstructed evolutionary history of sporangia annulus in ferns as in Figure 4. We have re- interpreted the evolutionary history and avoided the “ladderized thinking”.
This paragraph has been changed as “By observing the character of sporangial annulus of abundant samples in each fern group, and combining these characters with our well-resolved backbone phylogeny (Figure 3), we reconstructed the evolutionary history of sporangial annulus in ferns (Figure 4). According to the results, we infer that ex-annulus sporangia, as in Equisetaceae, Psilotaceae, and Ophioglossaceae, is the ancient state in ferns; rudimentary multiseriate annulus, which is inverse U-shaped in Marattiaceae, and U-shaped in Osmundaceae; equatorial transverse-oblique uniseriate annulus, as in Gleicheniaceae and Hymenophyllaceae; oblique annulus as in Cyatheales (tree ferns), and vertical annulus as synapomorphy in polypods, have been derived from the ex-annulus state. Both Apical annulus as in Lygodium and Schizaea, and vestige or disappeared annulus as in Salviniales (aquatic ferns) are likely to be specialized in parallel from oblique annulus” (page 11, line 220-231).

page 11, the monophyly of eusporangiate ferns is highlighted in Conclusion, but this is also one of the inconsistent relationship between the ASTRAL and concatenation analyses. I suggest perhaps discuss the incongruence and potential pitfalls in phylotranscriptomics instead.
R: Since the monophyly of eusporangiate ferns are supported by coalescent-based method, but not by concatenation-based method, here we only say that the monophyly of eusporangiate ferns is possible in both the abstract (page 2, line 30-31) and the conclusion (page 11, ling 240-242). In discussion, we say “The incongruence between the results based on coalescent and concatenation methods may be caused by strong ILS effect, which is a main pitfall when using concatenation method [21]”(page 8, line 168-171).

page 14, line 312, there are way more fossils that can be used as calibrations. Why only included two?
R: Fossil ages reflect the minimum times of divergence, which is more recent than the true divergence time. Given the small possibility in finding the earliest fossil for a lineage, and the difficulties in fossil species identification and correct dating, perfect fossils for calibration are rarely available. It is not always better to use more fossil dates as calibrations in estimation the divergence times for a phylogeny. Here we use two fossils (Archaeocalamites Senftenbergia: 354

MY, Grammatopteris: 280 MY) as the minimum ages of monilophytes and leptosporangiate ferns, which are relatively high in quality. When we use 8 fossil dates in calibration, the result did not improved remarkably.

change "sporangia annulus to "sporangial annulus"
R: "sporangia annulus” has been changed to "sporangial annulus" in this manuscript, thank you.

Reviewer #2: The revised manuscript deals with and addresses many of the methodological comments from the previous reviews. Importantly, data and scripts are all deposited on FigShare and can be accessed. They have also added summary methods in addition to their concatenation analyses before.

My remaining concerns about this manuscript are mostly related to individual sentences. These need to be revised for improved accuracy, clarity, or both.

Minor comments:
1- Page 4: " Moreover, due to the fact that amino-acid sequence is more conserved than nucleotide sequence, it may be more suited to estimate relationships among distant taxa. "
- The main issue with nucleotides is the fact that they tend to have compositional bias, especially in the third codon position. The discussions about what form of data is suited for "distant" or "recent" taxa is a bit hand-wavy, and in my view, misplaced. The important distinction between AA and nucleotide data is model fit.
R: We agree with your comment, and thanks for pointing out our misunderstanding. We have deleted this sentence in our revised manuscript, and made an careful interpretation about the advantage and disadvantage for protein and DNA data in phylogeny: “Nucleotide sequence, with higher variability than amino acid sequence, usually brings more useful information in phylogeny reconstruction, especially for closely related taxa. However, the compositional bias in nucleotide sequence, especially in the third codon position, may lead to a deviation from the true phylogeny. Here, both nucleotide and amino-acid sequences are used in phylogeny reconstruction” (page 4, line 73-78).

2. Calling parts of the tree "sites" is rather confusing. Sites typically are used for alignments. I would use another term (e.g., parts, areas, relationships, etc.).
R: “Sites” has been changed to “positions” in the revised manuscript (e.g. page 6, line 131).

3. Why authors keep referring to their estimated phylogenetic trees as cladograms and/or phylogram is unclear and non-standard. Why not just call them phylogenetic trees?
R: “Cladograms” and “phylogram” were used in the former manuscript to infer the phylogenetic trees without time calibration, they have been changed to “topology” instead (e.g. page 6, line 129).

4. Page 6: "Among the cladograms, the one estimated by applying coalescent-based method to nucleotide sequences (Figure 3) is the most agreed."
- I have no idea what this sentence means. What does it mean to say a tree is most agreed? Agreed with what?
- Also, if the authors clarify what they mean, still, whether agreement between different analyses

using the same tool means anything is not clear. That ASTRAL trees are more consistent among different analyses does not indicate higher quality of ASTRAL trees, and the authors should not imply that.
R: Thank you for the comments. “Most agreed” was to say that “the topology estimated by applying coalescent-based method to nucleotide sequences (Figure 3) is more consistent among different analyses”. Since we have no evidence that the topology estimated by applying coalescent-based method to nucleotide sequences (Figure 3) is necessarily more reliable than the one estimated by applying concatenation-based method, we have changed this sentence as “Among the topologies, the one estimated by applying coalescent-based method to nucleotide sequence (Figure 3) and the one applying concatenation-based method (Figure S2) are most congruent” (page 6, line 131-133).

5. Page 13: "To reduce the complexity of each group, we removed all sequences of the species that had more than 10 sequences in this group. "
- Again, it's not clear what is meant by "complexity" here. Phylogenetic analyses greatly benefit from increased taxon sampling, and if the authors have completely removed taxa from their analyses, that is not a good practice. The only justified reason I can think of is that with extra sequences, analyses would be infeasible computationally.
R: The de novo assembly by trinity results in many sequences with high similarity, which includes both paralogs and isoforms (Haas, 2013). The complexity mentioned here meant that if a clustered gene family contains too many sequences (eg. more than 10), the risk of contamination of isoforms instead of true paralogs will be raised. In addition, as commented by the reviewer, it is indeed infeasible computationally when the sequence number grows larger, since phylogeny trees are built for each gene family. Concerning the taxa number, the reviewer worried that if we remove all the sequence from the taxa in the gene family will reduce the taxa number in use. We set the threshold value for taxa cover degree that is 75% and 90%, so this process will not influence taxon sampling.
The de novo assembly by trinity produces many sequences with high similarity, which includes both paralogs and isoforms (Haas, 2013). The complexity mentioned here meant that if a clustered gene family contains too many sequences (eg. more than 10), the risk of contamination of isoforms instead of true paralogs will be raised. In addition, as commented by the reviewer, it is indeed infeasible computationally when the sequence number grows larger, since phylogeny trees are built for each gene family.
Concerning the taxa number, the reviewer worried that if we remove all the sequence from the taxa in the gene family will reduce the taxa number in use. We set the threshold value for taxa coverage that is 75% and 90%, so this process will not influence taxon sampling.
This paragraph has been changed as “As the de novo assembly by Trinity produces many sequences with high similarity, which contain both paralogs and isoforms [47], when a clustered gene family contains too many sequences (eg. more than 10), the risk of contamination of isoforms rises, along with the computational infeasibility. Hence, when a species has more than 10 sequences in a gene family, we remove all sequences in this gene family of this species” (page 13, line 272-277).

6. Page 13: "Out of 69 samples in total, 65 samples (that is 94.2% of total) were defined to have a relatively higher gene coverage degree. "
- Please be more specific. What if any threshold was used?

R: This sentence has been changed as “Out of the 69 samples in total, the gene coverage of 65 samples (94.2%) exceeded 82%, with at least 251 complete genes identified” (page 14, line 299-300).

7. Page 14: "Statistically consistency was estimated from unrooted gene trees under the multi-species coalescent model."
- This sentence has no meaning whatsoever. Authors don't seem to know what "statistical consistency" is and they should avoid making any claim about it. Remove or rephrase, please.
R: This sentence has been removed in the revised manuscript.

Reviewer #3: The authors satisfactory addressed all points raised in the previous round of review. The inclusion of a coalescent-based method to estimate the phylogeny is a very welcome addition and the differences in the obtained phylogenies are succinctly presented.
R: Thank you for the comments.
