# Supplementary material for: Large-scale phylogenomic analysis resolves a backbone phylogeny in ferns
Source: Gigascience. 2017 Nov 24;7(2):gix116. doi: 10.1093/gigascience/gix116 (PMC5795342; doi:10.1093/gigascience/gix116)
Supplement: Response-to-Reviewer-Comments_Revision-1.pdf [file gix116_response-to-reviewer-comments_revision-1.pdf]

Author's Response To Reviewer Comments

Reviewer #1:
The current manuscript is in no doubt much better than the first submission :) I have no major comment, but only a few minor editing suggestions:

page 1, line 5. Shouldn't the affiliation for Xian-Chun Zhang be "4" rather than "3"?
R: The affiliation for Xian-Chun Zhang should be "4", thank you very much! (page 1, line 5)

page 2, line 23-26. This sentence still needs to be clarified. Perhaps you could just say "The resulting topologies are largely congruent with each other, except the placement of [XXX]."
R: This sentence has been revised as "The resulting topologies are largely congruent with each other, except the placement of Angiopteris fokiensis, Cheiropleuria bicuspis, Diplaziopsis brunoniana, Matteuccia struthiopteris, Elaphoglossum mcclurei and Tectaria subpedata" (page 2, line 23-26).

page 2, line 32. "which is" can be dropped, i.e. "Gleicheniaceae and Hymenophyllaceae form a monophyletic clade sister to Dipteridaceae."
R: This sentence has been revised as "Gleicheniaceae and Hymenophyllaceae form a monophyletic clade sister to Dipteridaceae" (page 2, line 31-32).

page 3, line 57. Ophioglossales is misspelled
R: "Ophiglossales" has been changed as "Ophioglossales", thank you. (page 3, line 57)

page 3, line 60. I'd suggest to use "provides" instead of "represents"
R: "Represents" has been changed as "provides", thank you. (page 3, line 60)

page 4, line 72. The sentence was started with "Since"; therefore "so" here needs to be dropped.
R: This sentence has been revised as "Since concatenation-based estimations of species tree usually have good accuracy under low level of ILS, while coalescent-based methods are developed to overcome the effect of ILS, but are sensitive to gene tree estimation error [21], both concatenation-based and coalescent-based estimations are applied" (page 4, line 69-73). "So" in line 72 has been dropped. Thank you.

page 4, line 75. You could also mention here the saturation problem in nucleotide dataset.
R: This sentence has been revised as "However, the substitutional saturation and compositional bias in nucleotide sequence, especially in the third codon position, may lead to a deviation from the true phylogeny" (page 4, line 75-77).

page 6, line 112. Phytozome, not Phytozone
R: "Phytozone" has been changed to "Phytozome" (page 6, line 112).

page 7, line 149. The paper by Schuettpelz and Pryer (PNAS 2009) should be cited here to support "radiation in Cenozoic".
R: The paper by Schuettpelz and Pryer (PNAS 2009) has been cited to support "radiation in Cenozoic", this sentence has been changed as "Since eupolypods have experienced rapid

evolutionary radiation in Cenozoic [7], and nucleotide sequences usually provide more information to reconstruct relationships at shallow phylogenetic scale, we consider the topology yielded from nucleotide sequence maybe more reliable” (page 7, line 148-151).

page 7, line 153. Add "the", i.e. "are often the controversial nodes"
R: “The” has been added here as “are often the controversial nodes”(page 7, line 153).

page 8, line 160. "evidence" is not countable.
R: This sentence has been changed as “based on molecular evidence” (page 8, line 160).

page 10, line 215. I think "ancestral" is a better word than "primitive". Primitive has a bad connotation.
R: “Primitive” has been changed as “ancestral” (page 10, line 215).

page 11, line 224. Change "ancient" to "ancestral".
R: "Ancient" has been changed to "ancestral" (page 11, line 224).

page 11, line 229. Change "Apical" to "apical".
R: "Apical" has been changed to "apical" (page 11, line 229).

page 11, line 233. Again I'd suggest using "ancestral" instead of "primitive".
R: “Primitive” has been changed as “ancestral” (page 11, line 233).

page 19, line 449. Phytozome, not Phytozone
R: “Phytozone” has been changed to “Phytozome” (page 19, line 448).
