# Supplementary material for: Large-scale phylogenomic analysis resolves a backbone phylogeny in ferns
Source: Gigascience. 2017 Nov 24;7(2):gix116. doi: 10.1093/gigascience/gix116 (PMC5795342; doi:10.1093/gigascience/gix116)
Supplement: Reviewer-1-Report-(Original-Submission) -- Fay-Wei Li — 31 Jul 2017 Reviewed [file gix116_reviewer-1-report-(original-submission).pdf]

Reviewer Report

Title: Large scale phylogenomic analysis resolves a backbone phylogeny in ferns

Version: Original Submission **Date:** 7/31/2017

Reviewer name: Fay-Wei Li

Reviewer Comments to Author:

page 3, line 60, change "basing on" to "based on"
page 4, line 66, change "basing on" to "based on", and change "analysis" to "analyses"
page 4, line 74, change "close" to "closely"
page 5, line 91, change "Schezaeles" to "Schizaeales"
page 5, line 101, change "Genbank" to "NCBI"
page 5, line 103, change "filtration" to "filtering"
page 5, line 107, change "lineage" to "lineages"
page 6, line 127-130, suggested wording: "For each combination of reconstruction methods (coalescent-based or concatenation-based) and sequence types (nucleotides or amino acids), Matrix 1 and Matrix 2 always yielded the same topology. In general, the four cladograms (Figure 3, Figure S1, S2, S3) from a combination of methods and sequence types are consistent except six positions (Table 2)."
page 6, line 130-132, what do you mean by "most agreed"?
page 6, line 133, change "evolution" to "evolutionary"
page 7, line 149, change "among close related taxa" to "at shallow phylogenetic scale"
page 7, line 152-154, suggested wording: "..., and are often the controversial nodes from past studies based on different genes, we suggest such inconsistency might be caused partially by LIS and reticulate evolution."
page 8, line 158-159, Rothfels et al (2015) is not the first to report Equisetum being sister to the rest. See Rai and Graham (2010, AJB), and Kuo et al (2011, MPE). Also change "basing" to "based"
page 8, line 172, change "view of mainstream" to "mainstream"
page 9, line 179, no need to say "forking ferns" and "filmy ferns" again here.
page 9, line 181, change "Differently" to "On the other hand"
page 9, line 185, change "may form a sister lineage to" to "may be sister to"
page 9, line 186, change "the Gleicheniales order" to "Gleicheniales"
page 9, line 195, remove "the disputation of inner"
page 9, line 200, change "in agree with" to "in agreement with", and references are needed for this sentence.
page 10, line 202, change "new" to "different"
page 10, line 206, both "phylogram" and "cladogram" are used in this manuscript, and in a seemly interchangeable way. I'd prefer "topology".
page 10, line 208, change "more close to" to "more closely related to"
page 10, line 206-216, NO MORE USE OF "PRIMITIVE"!!! Everthing extant is equally "advanced".
page 10-11, "The evolution of sporangia annulus in ferns". I'm still having trouble understanding how

the authors deduce the "routes" of annulus evolution. The "two subroutes" is particularly confusing - it would only make sense if Schizaeales and Salviniales are monophyletic, which they are not. And again, the ladderized thinking - with Polypodiales having the advanced, final states while the others being the primitive intermediates - is not correct.
page 11, the monophyly of eusporangiate ferns is highlighted in Conclusion, but this is also one of the inconsistent relationship between the ASTRAL and concatenation analyses. I suggest perhaps discuss the incongruence and potential pitfalls in phylotranscriptomics instead.
page 14, line 312, there are way more fossils that can be used as calibrations. Why only included two?

change "sporangia annulus to "sporangial annulus"

Methods

Are the methods appropriate to the aims of the study, are they well described, and are necessary controls included? Yes

Conclusions

Are the conclusions adequately supported by the data shown? No

Reporting Standards

Does the manuscript adhere to the journal's guidelines on minimum standards of reporting? YesChoose an item.

Statistics

Are you able to assess all statistics in the manuscript, including the appropriateness of statistical tests used? Yes, and I have assessed the statistics in my report.

Quality of Written English

Please indicate the quality of language in the manuscript: Needs some language corrections before being published

Declaration of Competing Interests

Please complete a declaration of competing interests, considering the following questions:

- Have you in the past five years received reimbursements, fees, funding, or salary from an organisation that may in any way gain or lose financially from the publication of this manuscript, either now or in the future?
- Do you hold any stocks or shares in an organisation that may in any way gain or lose financially from the publication of this manuscript, either now or in the future?

- Do you hold or are you currently applying for any patents relating to the content of the manuscript?
- Have you received reimbursements, fees, funding, or salary from an organization that holds or has applied for patents relating to the content of the manuscript?
- Do you have any other financial competing interests?
- Do you have any non-financial competing interests in relation to this paper?

If you can answer no to all of the above, write 'I declare that I have no competing interests' below. If your reply is yes to any, please give details below.

I declare that I have no competing interests

I agree to the open peer review policy of the journal. I understand that my name will be included on my report to the authors and, if the manuscript is accepted for publication, my named report including any attachments I upload will be posted on the website along with the authors' responses. I agree for my report to be made available under an Open Access Creative Commons CC-BY license (http://creativecommons.org/licenses/by/4.0/). I understand that any comments which I do not wish to be included in my named report can be included as confidential comments to the editors, which will not be published.

I agree to the open peer review policy of the journal

To further support our reviewers, we have joined with Publons, where you can gain additional credit to further highlight your hard work (see: https://publons.com/journal/530/gigascience). On publication of this paper, your review will be automatically added to Publons, you can then choose whether or not to claim your Publons credit. I understand this statement.

Yes
