# Supplementary material for: Large-scale phylogenomic analysis resolves a backbone phylogeny in ferns
Source: Gigascience. 2017 Nov 24;7(2):gix116. doi: 10.1093/gigascience/gix116 (PMC5795342; doi:10.1093/gigascience/gix116)
Supplement: Reviewer-1-Report-(Revision-1) -- Fay-Wei Li — 06 Sep 2017 Reviewed [file gix116_reviewer-1-report-(revision-1).pdf]

Reviewer Report

Title: Large scale phylogenomic analysis resolves a backbone phylogeny in ferns

Version: Revision 1 **Date:** 9/6/2017

Reviewer name: Fay-Wei Li

Reviewer Comments to Author:

The current manuscript is in no doubt much better than the first submission :)

I have no major comment, but only a few minor editing suggestions:

page 1, line 5. Shouldn't the affiliation for Xian-Chun Zhang be "4" rather than "3"?
page 2, line 23-26. This sentence still needs to be clarified. Perhaps you could just say "The resulting topologies are largely congruent with each other, except the placement of [XXX]."
page 2, line 32. "which is" can be dropped, i.e. "Gleicheniaceae and Hymenophyllaceae form a monophyletic clade sister to Dipteridaceae."
page 3, line 57. Ophioglossales is misspelled
page 3, line 60. I'd suggest to use "provides" instead of "represents"
page 4, line 72. The sentence was started with "Since"; therefore "so" here needs to be dropped.
page 4, line 75. You could also mention here the saturation problem in nucleotide dataset.
page 6, line 112. Phytozome, not Phytozone
page 7, line 149. The paper by Schuettpelz and Pryer (PNAS 2009) should be cited here to support "radiation in Cenozoic".
page 7, line 153. Add "the", i.e. "are often the controversial nodes"
page 8, line 160. "evidence" is not countable.
page 10, line 215. I think "ancestral" is a better word than "primitive". Primitive has a bad connotation.
page 11, line 224. Change "ancient" to "ancestral".
page 11, line 229. Change "Apical" to "apical".
page 11, line 233. Again I'd suggest using "ancestral" instead of "primitive".
page 19, line 449. Phytozome, not Phytozone

Methods

Are the methods appropriate to the aims of the study, are they well described, and are necessary controls included? Yes

Conclusions

Are the conclusions adequately supported by the data shown? Yes

Reporting Standards

Does the manuscript adhere to the journal's guidelines on minimum standards of reporting? YesChoose an item.

Statistics

Are you able to assess all statistics in the manuscript, including the appropriateness of statistical tests used? Yes, and I have assessed the statistics in my report.

Quality of Written English

Please indicate the quality of language in the manuscript: Acceptable

Declaration of Competing Interests

Please complete a declaration of competing interests, considering the following questions:

- Have you in the past five years received reimbursements, fees, funding, or salary from an organisation that may in any way gain or lose financially from the publication of this manuscript, either now or in the future?
- Do you hold any stocks or shares in an organisation that may in any way gain or lose financially from the publication of this manuscript, either now or in the future?
- Do you hold or are you currently applying for any patents relating to the content of the manuscript?
- Have you received reimbursements, fees, funding, or salary from an organization that holds or has applied for patents relating to the content of the manuscript?
- Do you have any other financial competing interests?
- Do you have any non-financial competing interests in relation to this paper?

If you can answer no to all of the above, write 'I declare that I have no competing interests' below. If your reply is yes to any, please give details below.

I declare that I have no competing interests

I agree to the open peer review policy of the journal. I understand that my name will be included on my report to the authors and, if the manuscript is accepted for publication, my named report including any attachments I upload will be posted on the website along with the authors' responses. I agree for my report to be made available under an Open Access Creative Commons CC-BY license (http://creativecommons.org/licenses/by/4.0/). I understand that any comments which I do not wish to be included in my named report can be included as confidential comments to the editors, which will not be published.

I agree to the open peer review policy of the journal

To further support our reviewers, we have joined with Publons, where you can gain additional credit to further highlight your hard work (see: https://publons.com/journal/530/gigascience). On publication of this paper, your review will be automatically added to Publons, you can then choose whether or not to claim your Publons credit. I understand this statement.

Yes
