# Supplementary material for: Large-scale phylogenomic analysis resolves a backbone phylogeny in ferns
Source: Gigascience. 2017 Nov 24;7(2):gix116. doi: 10.1093/gigascience/gix116 (PMC5795342; doi:10.1093/gigascience/gix116)
Supplement: Reviewer-2-Report-(Original-Submission) -- Siavash Mirarab — 01 Aug 2017 Reviewed [file gix116_reviewer-2-report-(original-submission).pdf]

Reviewer Report

Title: Large scale phylogenomic analysis resolves a backbone phylogeny in ferns

Version: Original Submission **Date:** 8/1/2017

Reviewer name: Siavash Mirarab

Reviewer Comments to Author:

The revised manuscript deals with and addresses many of the methodological comments from the previous reviews. Importantly, data and scripts are all deposited on FigShare and can be accessed. They have also added summary methods in addition to their concatenation analyses before.

My remaining concerns about this manuscript are mostly related to individual sentences. These need to be revised for improved accuracy, clarity, or both.

Minor comments:

1- Page 4: " Moreover, due to the fact that amino-acid sequence is more conserved than nucleotide sequence, it may be more suited to estimate relationships among distant taxa. "
- The main issue with nucleotides is the fact that they tend to have compositional bias, especially in the third codon position. The discussions about what form of data is suited for "distant" or "recent" taxa is a bit hand-wavy, and in my view, misplaced. The important distinction between AA and nucleotide data is model fit.

2. Calling parts of the tree "sites" is rather confusing. Sites typically are used for alignments. I would use another term (e.g., parts, areas, relationships, etc.).

3. Why authors keep referring to their estimated phylogenetic trees as cladograms and/or phylogram is unclear and non-standard. Why not just call them phylogenetic trees?

4. Page 6: "Among the cladograms, the one estimated by applying coalescent-based method to nucleotide sequences (Figure 3) is the most agreed."
- I have no idea what this sentence means. What does it mean to say a tree is most agreed? Agreed with what?
- Also, if the authors clarify what they mean, still, whether agreement between different analyses using the same tool means anything is not clear. That ASTRAL trees are more consistent among different analyses does not indicate higher quality of ASTRAL trees, and the authors should not imply that.

5. Page 13: "To reduce the complexity of each group, we removed all sequences of the species that had more than 10 sequences in this group. "
- Again, it's not clear what is meant by "complexity" here. Phylogenetic analyses greatly benefit from

increased taxon sampling, and if the authors have completely removed taxa from their analyses, that is not a good practice. The only justified reason I can think of is that with extra sequences, analyses would be infeasible computationally.

6. Page 13: "Out of 69 samples in total, 65 samples (that is 94.2% of total) were defined to have a relatively higher gene coverage degree. "
- Please be more specific. What if any threshold was used?

6. Page 14: "Statistically consistency was estimated from unrooted gene trees under the multi-species coalescent model."
- This sentence has no meaning whatsoever. Authors don't seem to know what "statistical consistency" is and they should avoid making any claim about it. Remove or rephrase, please.

Methods

Are the methods appropriate to the aims of the study, are they well described, and are necessary controls included? Yes

Conclusions

Are the conclusions adequately supported by the data shown? Yes

Reporting Standards

Does the manuscript adhere to the journal's guidelines on minimum standards of reporting? YesChoose an item.

Statistics

Are you able to assess all statistics in the manuscript, including the appropriateness of statistical tests used? There are no statistics in the manuscript.

Quality of Written English

Please indicate the quality of language in the manuscript: Needs some language corrections before being published

Declaration of Competing Interests

Please complete a declaration of competing interests, considering the following questions:

- Have you in the past five years received reimbursements, fees, funding, or salary from an organisation that may in any way gain or lose financially from the publication of this manuscript, either now or in the future?

- Do you hold any stocks or shares in an organisation that may in any way gain or lose financially from the publication of this manuscript, either now or in the future?
- Do you hold or are you currently applying for any patents relating to the content of the manuscript?
- Have you received reimbursements, fees, funding, or salary from an organization that holds or has applied for patents relating to the content of the manuscript?
- Do you have any other financial competing interests?
- Do you have any non-financial competing interests in relation to this paper?

If you can answer no to all of the above, write 'I declare that I have no competing interests' below. If your reply is yes to any, please give details below.

I declare that I have no competing interests

I agree to the open peer review policy of the journal. I understand that my name will be included on my report to the authors and, if the manuscript is accepted for publication, my named report including any attachments I upload will be posted on the website along with the authors' responses. I agree for my report to be made available under an Open Access Creative Commons CC-BY license (http://creativecommons.org/licenses/by/4.0/). I understand that any comments which I do not wish to be included in my named report can be included as confidential comments to the editors, which will not be published.

I agree to the open peer review policy of the journal

To further support our reviewers, we have joined with Publons, where you can gain additional credit to further highlight your hard work (see: https://publons.com/journal/530/gigascience). On publication of this paper, your review will be automatically added to Publons, you can then choose whether or not to claim your Publons credit. I understand this statement.

Yes
